# Supplementary figures and images for: Concatemer-assisted stoichiometry analysis: targeted mass spectrometry for protein quantification
Source: Life Sci Alliance. 2024 Dec 31;8(3):e202403007. doi: 10.26508/lsa.202403007 (PMC11707388; doi:10.26508/lsa.202403007)

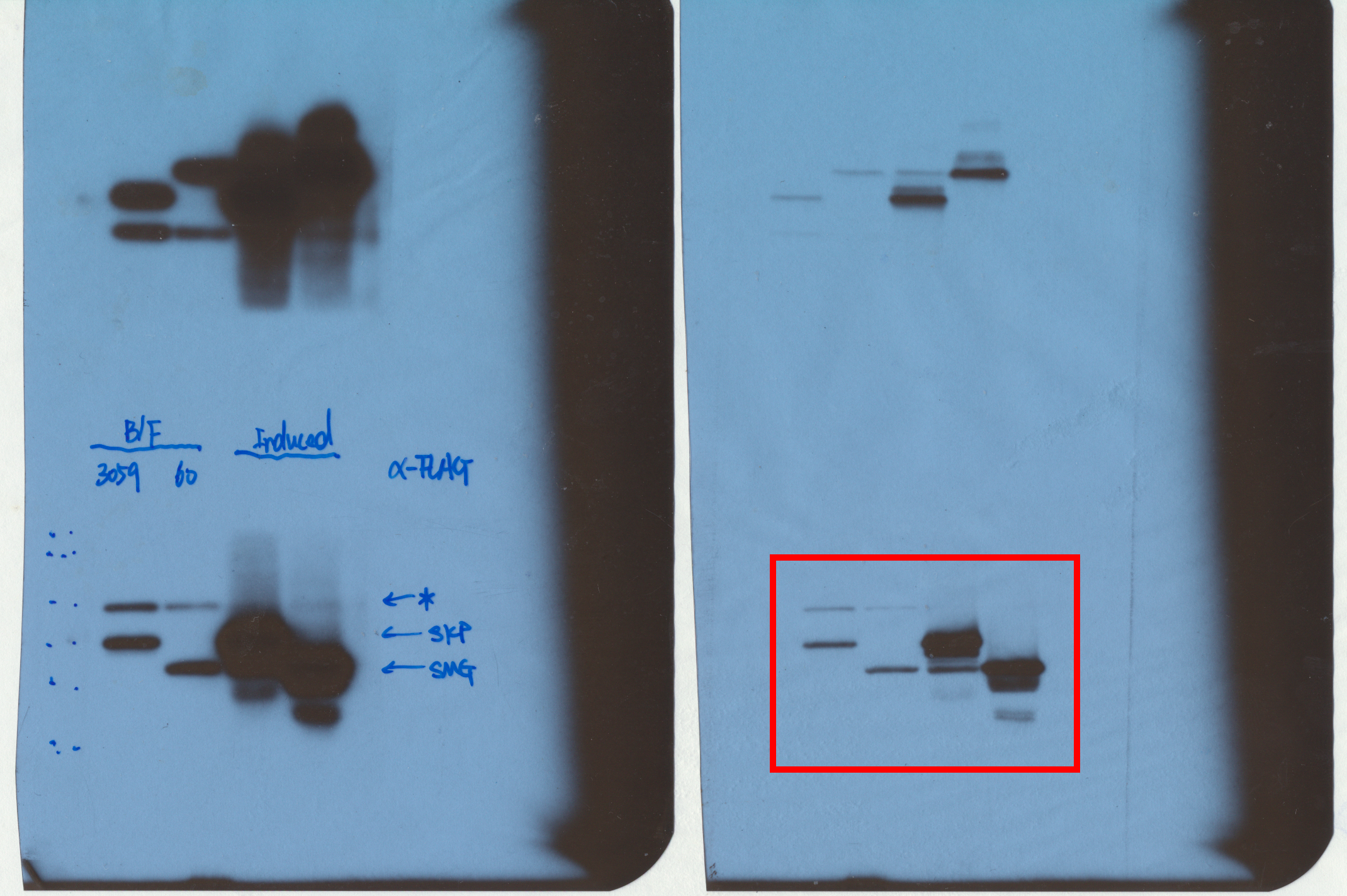

Supplement: Supplementary file 1 [file LSA-2024-03007_SdataFS1.1.tif]

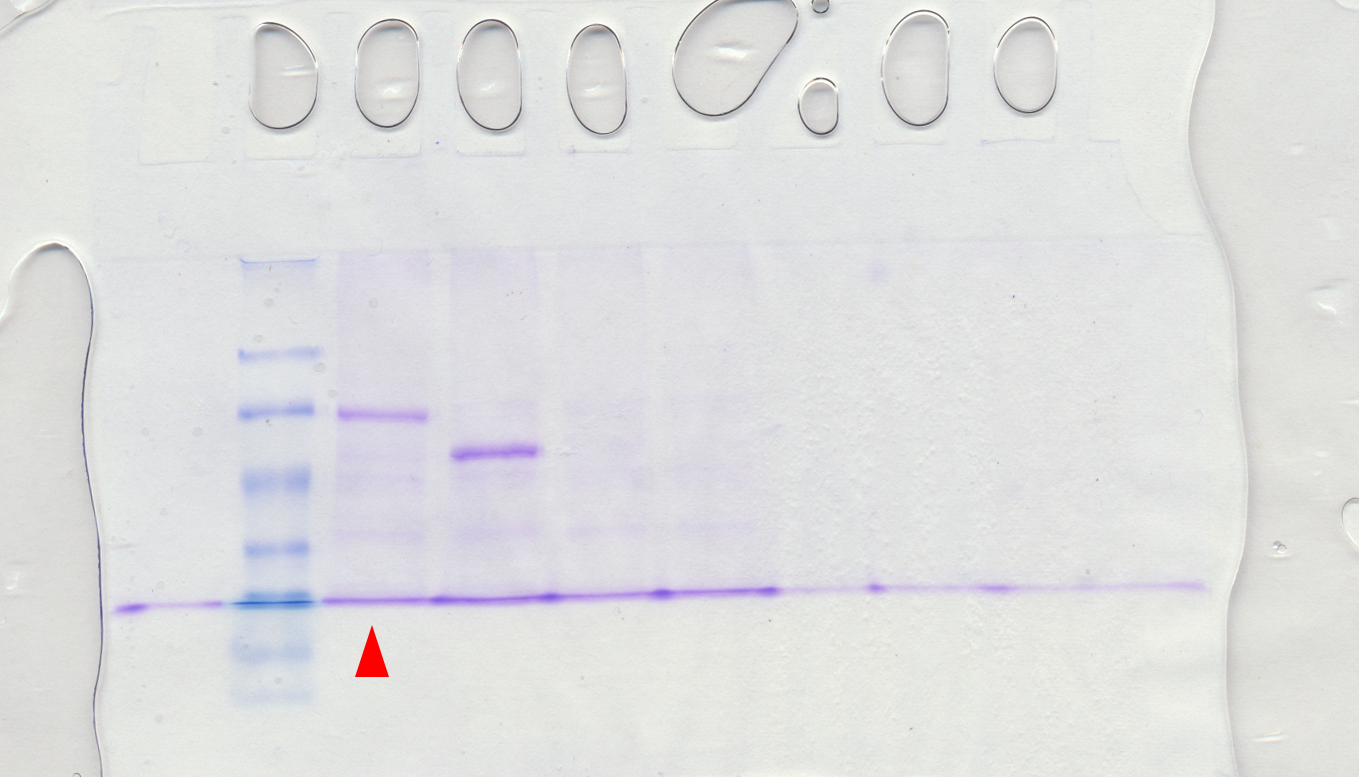

Supplement: Supplementary file 2 [file LSA-2024-03007_SdataFS1.2.tif]

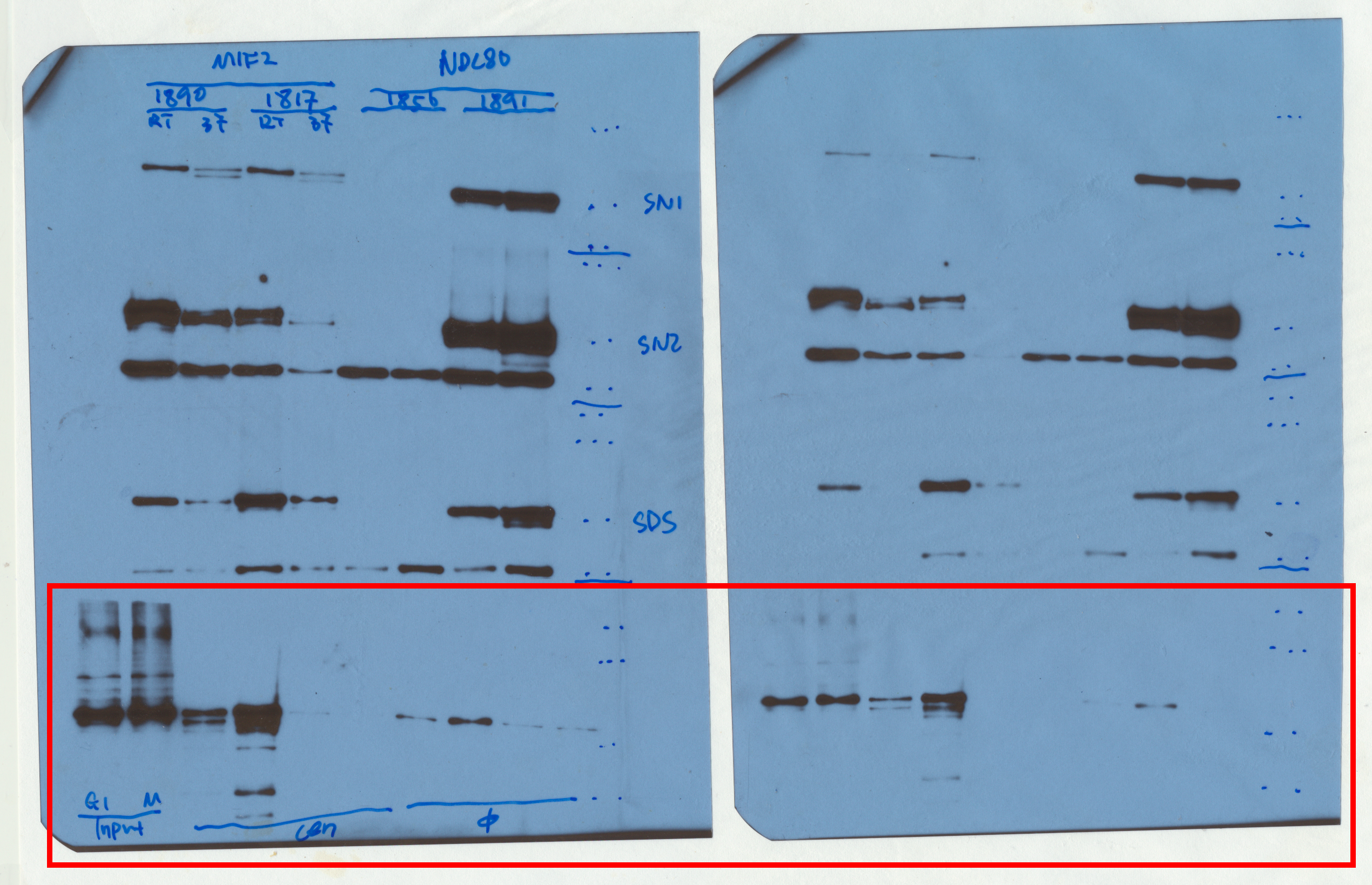

Supplement: Supplementary file 17 [file LSA-2024-03007_SdataFS3.tif]

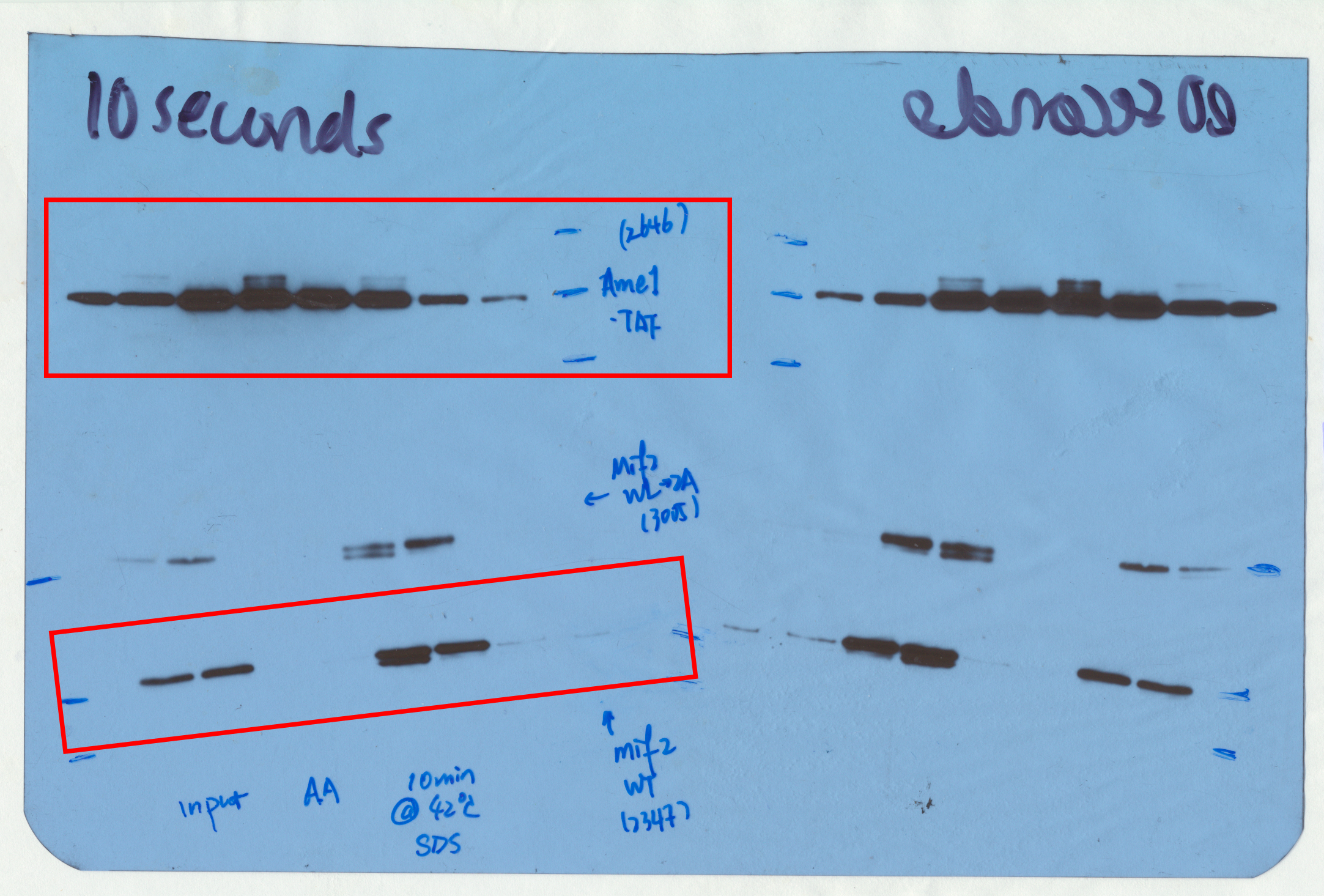

Supplement: Supplementary file 24 [file LSA-2024-03007_SdataFS8.1.tif]

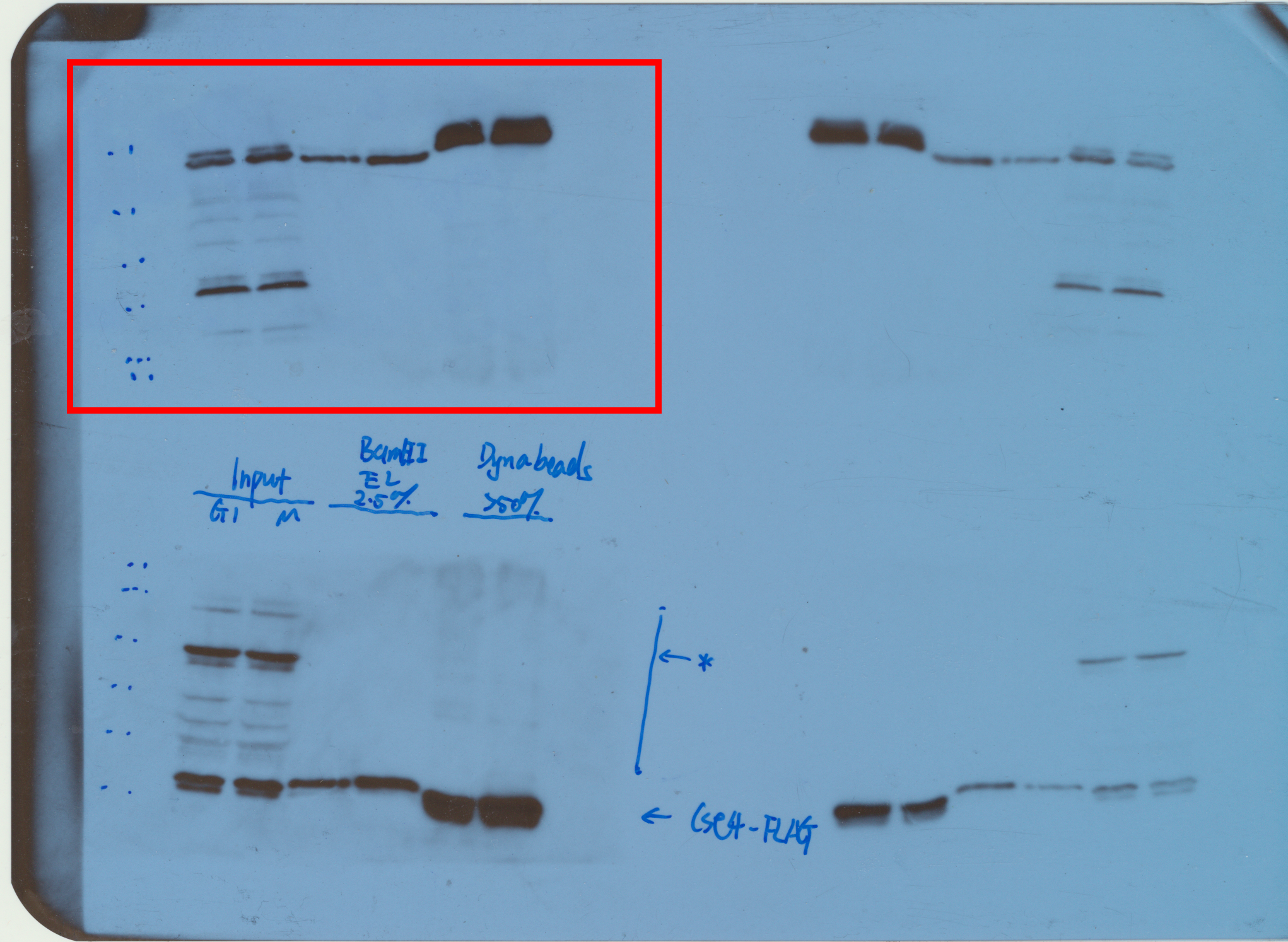

Supplement: Supplementary file 25 [file LSA-2024-03007_SdataFS8.2.tif]
