# Supplementary material for: Concatemer-assisted stoichiometry analysis: targeted mass spectrometry for protein quantification
Source: Life Sci Alliance. 2024 Dec 31;8(3):e202403007. doi: 10.26508/lsa.202403007 (PMC11707388; doi:10.26508/lsa.202403007)
Supplement: Supplementary file 4 [file LSA-2024-03007_TableS1.docx]

## Table S1. Isotope incorporation efficiencies of H-CKP.

The peak areas shown are the sum of the peak areas of the top 12 transitions.

| **Peptide** | **Heavy area** | **Light area** | **% Heavy of total area** |
| --- | --- | --- | --- |
| **Hta2** | 17078343 | 0 | 100.00 |
| **Ctf3** | 20109657 | 0 | 100.00 |
| **Cbf2** | 19143204 | 1570 | 99.99 |
| **Nkp1** | 22143101 | 20836 | 99.91 |
| **Iml3** | 20163754 | 0 | 100.00 |
| **Htb2** | 8778000 | 10597 | 99.88 |
| **Mcm21** | 32181955 | 1491 | 100.00 |
| **Dsn1** | 38273619 | 0 | 100.00 |
| **Mtw1** | 40463111 | 0 | 100.00 |
| **Hhf1** | 24330620 | 0 | 100.00 |
| **Cbf1** | 67708609 | 57839 | 99.91 |
| **Cep3** | 22579733 | 0 | 100.00 |
| **Ame1** | 14451112 | 0 | 100.00 |
| **Chl4** | 44155600 | 62964 | 99.86 |
| **Ctf19** | 27480906 | 1417 | 99.99 |
| **Ndc80** | 26923566 | 0 | 100.00 |
| **Cnn1** | 13304552 | 1245 | 99.99 |
| **Hht1** | 29087574 | 0 | 100.00 |
| **Ctf13** | 28351430 | 0 | 100.00 |
| **Mif2-2** | 30827943 | 105111 | 99.66 |
| **Spc105** | 16052332 | 0 | 100.00 |
| **Okp1** | 10856738 | 0 | 100.00 |
| **Nkp2*** | 18603646 | 391167* | 97.94* |
| **Mif2-1** | 41905182 | 0 | 100.00 |
| **Cse4** | 56761875 | 2634 | 100.00 |

* Most abundant transition (b2) is interfered with by a contaminant.
