# Supplementary material for: Concatemer-assisted stoichiometry analysis: targeted mass spectrometry for protein quantification
Source: Life Sci Alliance. 2024 Dec 31;8(3):e202403007. doi: 10.26508/lsa.202403007 (PMC11707388; doi:10.26508/lsa.202403007)
Supplement: Supplementary file 10 [file LSA-2024-03007_TableS3.docx]

## Table S3. Accuracy of calibrators (Tolerance: ± 20%).

Gray cells are outside the AMR (Tolerance: ± 20%). Cells with yellow highlights have %biases between -20% and -15% or 15% and 20%. The rest of the cells have %biases within -15% and 15%.

| **Peptide** | **Calibrator %Bias** | | | | | | | | | |
| --- | --- | --- | --- | --- | --- | --- | --- | --- | --- | --- |
| **Ame1** | 18.0 | 5.2 | -1.2 | 1.9 | -10.0 | -4.6 | -3.5 | -3.9 | -5.0 | 3.0 |
| **Cbf1** | 7.7 | 9.2 | -1.6 | -0.7 | -2.5 | -0.6 | -0.3 | -6.9 | -8.7 | 4.1 |
| **Cbf2** | -189.6 | -79.6 | -42.6 | -3.3 | -6.0 | 7.2 | 8.9 | 3.3 | -13.0 | 2.9 |
| **Cep3** | 3.3 | 3.1 | -5.9 | 8.4 | -1.2 | -2.0 | 1.7 | 3.3 | -15.1 | 4.4 |
| **Chl4** | -16.3 | -5.8 | -4.7 | 3.3 | 5.2 | 10.5 | 11.5 | 0.6 | -4.1 | -0.2 |
| **Cnn1** | 2423.7 | 1168.7 | 545.7 | 234.3 | 84.7 | 15.9 | -14.4 | -6.2 | 4.7 | 29.8 |
| **Cse4** | 137.4 | 49.8 | 11.0 | -7.3 | -8.4 | 4.6 | 35.8 | 63.7 | 76.6 | 100.5 |
| **Ctf13** | -22.7 | 0.8 | -8.0 | 2.1 | 0.2 | 1.0 | 5.8 | -0.7 | -1.2 | 16.3 |
| **Ctf19** | 37.6 | 6.5 | 2.6 | 0.5 | 2.3 | -12.5 | 3.9 | -4.1 | -21.9 | 0.8 |
| **Ctf3** | 2.2 | -1.1 | -8.6 | 4.1 | -1.8 | -0.1 | 6.5 | -0.9 | -21.4 | -0.4 |
| **Dsn1** | 8.7 | -1.9 | -2.6 | -2.5 | -7.3 | -1.5 | 6.2 | 1.5 | -24.2 | -0.5 |
| **Hhf1** | 544.7 | 238.2 | 106.0 | 40.0 | 13.4 | -7.4 | -0.5 | -6.7 | -20.3 | 1.2 |
| **Hht1** | 8.3 | -11.9 | 0.8 | 3.2 | 2.7 | -2.6 | -3.1 | -1.0 | -0.6 | 0.6 |
| **Hta2** | 0.7 | 0.7 | -2.9 | 8.4 | -0.1 | -4.0 | 1.5 | -5.1 | -21.5 | 0.8 |
| **Htb2** | 14.0 | -10.2 | -6.2 | 2.8 | -1.6 | 3.1 | -1.5 | -2.3 | -1.4 | 0.8 |
| **Iml3** | 0.4 | 0.1 | -4.9 | 11.0 | -3.5 | 2.6 | -1.4 | -0.3 | -6.1 | 2.1 |
| **Mcm21** | -3.9 | -3.5 | 3.4 | 5.8 | -1.7 | -1.1 | 5.5 | -2.0 | -3.5 | 1.0 |
| **Mif2-1** | -232.3 | -107.8 | -46.6 | -9.1 | -1.1 | 4.2 | 1.8 | 5.3 | -13.8 | -1.1 |
| **Mif2-2** | 14.3 | -1.5 | 7.6 | 4.8 | -4.3 | -14.1 | -2.0 | -8.7 | -25.7 | 2.0 |
| **Mtw1** | 190.8 | 63.5 | 13.7 | -13.1 | -4.3 | 3.7 | 38.3 | 54.7 | 64.0 | 89.9 |
| **Ndc80** | 1.9 | 19.2 | -9.8 | -1.0 | -1.9 | -5.3 | 0.1 | -4.0 | -27.2 | 0.9 |
| **Nkp1** | -63.2 | -45.2 | -39.5 | -1.8 | -6.1 | 9.1 | 5.5 | 2.8 | -12.6 | 3.0 |
| **Nkp2** | -98.6 | -43.7 | -8.8 | -3.9 | 1.0 | 6.5 | 4.0 | 3.7 | -2.2 | -0.4 |
| **Okp1** | -36.2 | -11.5 | 2.6 | 8.5 | 0.2 | 4.7 | 0.4 | -0.8 | -5.6 | 1.7 |
| **Spc105** | 7.2 | -6.3 | 1.6 | -4.8 | -1.3 | -0.5 | 3.6 | 2.0 | -21.2 | -0.5 |
| **Cse4 - High range** | 1876.7 | 903.9 | 419.9 | 178.5 | 61.8 | 10.1 | -3.4 | -4.0 | -4.9 | 2.2 |
| **Mtw1 - High range** | 1345.2 | 633.1 | 285.1 | 110.0 | 35.3 | 0.1 | 0.1 | -0.3 | 0.1 | 11.8 |
| **Analyte conc. (pM)** | **78** | **156** | **313** | **625** | **1250** | **2500** | **5000** | **10000** | **20000** | **60000** |
