# Supplementary material for: Concatemer-assisted stoichiometry analysis: targeted mass spectrometry for protein quantification
Source: Life Sci Alliance. 2024 Dec 31;8(3):e202403007. doi: 10.26508/lsa.202403007 (PMC11707388; doi:10.26508/lsa.202403007)
Supplement: Supplementary file 11 [file LSA-2024-03007_TableS4.docx]

## Table S4. Inter-day accuracy of calibrators (Tolerance: ± 20%).

Gray cells are outside the AMR (Tolerance: ± 20%). Cells with yellow highlights have average %biases between -20% and -15% or 15% and 20%. The rest of the cells have average %biases within -15% and 15%.

| **Peptide** | **Calibrator Avg. %Bias (n = 3)** | | | | | | | | | |
| --- | --- | --- | --- | --- | --- | --- | --- | --- | --- | --- |
| **Ame1** | 19.5 | 6.8 | 0.3 | 1.8 | -9.9 | -1.6 | -1.7 | -1.6 | -3.5 | 3.5 |
| **Cbf1** | 10.2 | 7.7 | -0.7 | 3.1 | -3.0 | -5.9 | -19.1 | 0.5 | -4.1 | 2.0 |
| **Cbf2** | -194.2 | -97.6 | -44.3 | -7.3 | -5.4 | 3.3 | 8.2 | 4.8 | -7.6 | -0.5 |
| **Cep3** | 2.4 | 8.5 | 2.9 | 7.1 | 1.4 | -1.4 | -2.5 | 1.7 | -12.0 | 6.6 |
| **Chl4** | -12.7 | -2.1 | 1.6 | 10.5 | 7.1 | 12.8 | 9.4 | 3.5 | -5.8 | 1.4 |
| **Cnn1** | 2483.7 | 1178.3 | 532.7 | 227.9 | 78.8 | 16.6 | -14.2 | -4.3 | 12.6 | 27.8 |
| **Cse4** | 155.6 | 50.9 | 11.9 | -12.1 | -5.9 | 5.3 | 36.0 | 59.7 | 82.8 | 95.0 |
| **Ctf13** | -30.2 | -4.1 | 5.7 | 8.8 | -0.5 | 6.9 | 1.9 | 0.8 | -2.7 | 9.8 |
| **Ctf19** | 43.8 | 9.9 | 4.3 | -6.9 | -1.5 | -11.9 | 1.0 | 0.3 | -26.2 | 2.3 |
| **Ctf3** | 9.1 | 2.6 | -0.2 | -1.0 | -0.8 | -1.4 | 4.2 | 0.7 | -24.7 | -2.0 |
| **Dsn1** | 2.7 | -6.8 | -2.6 | -7.8 | -3.1 | -11.4 | 7.6 | 0.0 | -26.9 | 4.0 |
| **Hhf1** | 543.0 | 243.7 | 123.6 | 34.4 | 15.8 | -7.8 | -3.3 | -2.3 | -24.0 | 1.5 |
| **Hht1** | -28.6 | -10.8 | -9.4 | 10.5 | -0.1 | -2.3 | -1.7 | -3.9 | -3.8 | 3.7 |
| **Hta2** | 2.3 | 6.5 | 0.4 | 4.6 | 0.3 | -1.0 | -1.3 | -2.1 | -18.8 | -0.7 |
| **Htb2** | 301.2 | 64.0 | 12.6 | 7.4 | -3.2 | 1.8 | 2.2 | 2.0 | -2.1 | -1.2 |
| **Iml3** | -10.2 | -1.7 | -0.3 | 4.5 | -3.6 | -0.4 | -2.4 | -8.3 | -7.5 | -0.3 |
| **Mcm21** | -5.6 | -2.2 | 1.7 | 5.1 | -5.7 | 1.0 | 0.0 | -5.7 | -5.4 | 6.6 |
| **Mif2-1** | -243.1 | -125.9 | -49.0 | -16.2 | -5.3 | -3.6 | 5.3 | 5.3 | -22.9 | 3.0 |
| **Mif2-2** | 7.9 | -2.8 | 7.8 | -7.0 | -0.5 | -13.7 | 4.9 | -5.4 | -25.4 | -5.2 |
| **Mtw1** | 203.4 | 65.2 | 15.1 | -15.3 | -10.4 | -0.8 | 41.8 | 65.7 | 82.1 | 90.9 |
| **Ndc80** | -15.0 | -6.6 | -3.9 | -5.2 | -0.8 | -9.1 | 6.1 | -5.9 | -22.1 | -0.4 |
| **Nkp1** | -94.3 | -51.3 | -20.9 | -11.0 | 1.8 | 1.2 | 6.4 | 1.4 | -10.3 | 5.0 |
| **Nkp2** | -68.5 | -26.8 | -4.1 | 10.2 | -0.8 | 3.2 | 5.5 | 4.7 | 5.2 | 11.0 |
| **Okp1** | -40.2 | -18.0 | 0.5 | 11.3 | 1.2 | 3.8 | 2.5 | -3.1 | 0.7 | 2.4 |
| **Spc105** | 116.9 | 18.6 | -0.3 | 1.0 | -4.1 | -3.1 | 7.2 | -3.1 | -22.5 | 4.8 |
| **Cse4 - High range** | 1885.8 | 904.4 | 420.3 | 176.1 | 63.0 | 10.5 | -3.3 | -6.0 | -1.8 | -0.6 |
| **Mtw1 - High range** | 1352.6 | 634.1 | 285.9 | 108.7 | 31.8 | -2.5 | 2.2 | 6.1 | 10.7 | 12.4 |
| **Analyte conc. (pM)** | **78** | **156** | **313** | **625** | **1250** | **2500** | **5000** | **10000** | **20000** | **60000** |
