# Supplementary material for: Concatemer-assisted stoichiometry analysis: targeted mass spectrometry for protein quantification
Source: Life Sci Alliance. 2024 Dec 31;8(3):e202403007. doi: 10.26508/lsa.202403007 (PMC11707388; doi:10.26508/lsa.202403007)
Supplement: Supplementary file 12 [file LSA-2024-03007_TableS5.docx]

## Table S5. Inter-day precision of calibrators (Tolerance: ± 15%).

Gray cells are outside the AMR (Tolerance: ± 15%). Cells with yellow highlights have %CVs between -15% and -10% or 10% and 15%. The rest of the cells have %CVs within -10% and 10%.

| **Peptide** | **Calibrator %CV (n = 3)** | | | | | | | | | |
| --- | --- | --- | --- | --- | --- | --- | --- | --- | --- | --- |
| **Ame1** | 3.6 | 2.9 | 1.7 | 5.8 | 2.6 | 3.2 | 0.7 | 2.4 | 1.0 | 0.6 |
| **Cbf1** | 4.6 | 5.5 | 5.0 | 2.2 | 0.7 | 5.0 | 16.1 | 4.0 | 2.7 | 6.6 |
| **Cbf2** | -4.6 | 301.9 | 7.4 | 6.5 | 5.0 | 4.8 | 5.3 | 1.8 | 2.2 | 1.7 |
| **Cep3** | 11.2 | 0.6 | 1.1 | 3.2 | 1.8 | 9.9 | 1.9 | 0.1 | 0.8 | 9.9 |
| **Chl4** | 8.0 | 6.5 | 7.9 | 3.9 | 5.1 | 6.0 | 2.0 | 1.7 | 1.7 | 1.1 |
| **Cnn1** | 2.1 | 1.5 | 0.5 | 3.3 | 4.4 | 1.3 | 1.4 | 5.1 | 7.7 | 1.4 |
| **Cse4** | 4.8 | 3.6 | 2.6 | 0.9 | 2.4 | 4.3 | 1.8 | 1.6 | 1.0 | 0.9 |
| **Ctf13** | 10.5 | 1.9 | 5.4 | 2.6 | 0.2 | 4.2 | 2.3 | 6.8 | 3.3 | 3.5 |
| **Ctf19** | 3.0 | 3.1 | 3.2 | 2.3 | 2.5 | 1.3 | 4.7 | 2.4 | 4.1 | 0.5 |
| **Ctf3** | 14.5 | 4.2 | 2.0 | 6.4 | 5.2 | 2.4 | 4.3 | 3.1 | 2.5 | 4.3 |
| **Dsn1** | 14.1 | 5.0 | 0.8 | 5.2 | 4.7 | 4.1 | 5.9 | 4.2 | 2.8 | 2.6 |
| **Hhf1** | 2.4 | 1.7 | 4.1 | 7.0 | 2.5 | 1.0 | 2.3 | 1.3 | 4.5 | 2.6 |
| **Hht1** | 10.8 | 17.4 | 5.3 | 10.6 | 3.7 | 1.7 | 2.1 | 6.2 | 7.2 | 4.5 |
| **Hta2** | 3.4 | 6.0 | 3.7 | 6.8 | 4.7 | 1.9 | 2.4 | 1.6 | 3.1 | 1.0 |
| **Htb2** | 3.9 | 5.6 | 4.8 | 2.3 | 2.6 | 4.3 | 2.6 | 2.9 | 2.9 | 5.3 |
| **Iml3** | 8.5 | 4.5 | 1.4 | 4.7 | 9.5 | 2.5 | 2.9 | 4.1 | 3.2 | 0.9 |
| **Mcm21** | 10.8 | 2.2 | 1.4 | 0.1 | 0.4 | 4.1 | 2.7 | 1.2 | 0.5 | 2.6 |
| **Mif2-1** | -2.6 | -13.7 | 7.3 | 6.8 | 5.3 | 3.6 | 4.2 | 7.6 | 2.7 | 3.8 |
| **Mif2-2** | 8.6 | 7.5 | 2.1 | 5.9 | 7.4 | 5.5 | 16.1 | 5.5 | 7.0 | 3.5 |
| **Mtw1** | 2.4 | 1.7 | 1.9 | 2.2 | 1.0 | 1.5 | 1.2 | 4.6 | 5.3 | 3.8 |
| **Ndc80** | 7.6 | 10.2 | 9.4 | 5.0 | 4.9 | 2.8 | 5.3 | 10.0 | 3.7 | 3.4 |
| **Nkp1** | 227.1 | 16.6 | 11.7 | 2.6 | 4.0 | 3.2 | 7.3 | 8.1 | 8.4 | 6.5 |
| **Nkp2** | 71.8 | 7.5 | 5.3 | 6.1 | 1.7 | 7.8 | 4.4 | 5.8 | 3.7 | 5.3 |
| **Okp1** | 13.4 | 5.8 | 5.2 | 0.9 | 1.7 | 0.5 | 2.3 | 5.5 | 2.8 | 2.8 |
| **Spc105** | 4.9 | 14.1 | 6.4 | 6.1 | 12.9 | 3.5 | 0.6 | 2.8 | 2.2 | 4.6 |
| **Cse4 - High range** | 0.3 | 0.3 | 0.3 | 0.1 | 0.7 | 2.0 | 1.2 | 1.4 | 1.0 | 0.9 |
| **Mtw1 - High range** | 0.3 | 0.2 | 0.3 | 0.5 | 0.4 | 0.9 | 1.0 | 4.2 | 5.1 | 3.7 |
| **Analyte conc. (pM)** | **78** | **156** | **313** | **625** | **1250** | **2500** | **5000** | **10000** | **20000** | **60000** |
