# Supplementary material for: Concatemer-assisted stoichiometry analysis: targeted mass spectrometry for protein quantification
Source: Life Sci Alliance. 2024 Dec 31;8(3):e202403007. doi: 10.26508/lsa.202403007 (PMC11707388; doi:10.26508/lsa.202403007)
Supplement: Supplementary file 13 [file LSA-2024-03007_TableS6.docx]

## Table S6. Quantifier and qualifier ions (L-CKP).

Quantifier and qualifier transition ions for the L-CKP and corresponding ion ratios as determined from the average of the calibrators.

| **Peptide** | **Transitions** | | | | **Quant/Qual ion ratio** |
| --- | --- | --- | --- | --- | --- |
|  | **Quantifier** | **Quantifier m/z** | **Qualifier** | **Qualifier m/z** |  |
| **Cbf1** | T - y10++ | 593.7704 | S - y11++ | 637.2864 | 1.33 |
| **Cep3** | Y - y5+ | 681.3566 | T - y3+ | 405.2092 | 5.62 |
| **Ctf13** | D - y4+ | 538.2620 | L - b3+ | 272.1605 | 1.10 |
| **Cse4** | A - y6+ | 764.4301 | L - y5+ | 693.3930 | 1.45 |
| **Htb2** | A - b2+ | 209.1033 | V - y6+ | 648.3311 | 1.94 |
| **Hta2** | P - y4+ | 428.2616 | F - y5+ | 575.3300 | 1.19 |
| **Hhf1** | Y - y5+ | 695.3359 | I - y6+ | 808.4199 | 1.19 |
| **Hht1** | E - y5+ | 643.4137 | L - y3+ | 401.2871 | 1.18 |
| **Mif2-1** | S - y5+ | 545.3042 | S - y7+ | 761.3788 | 1.43 |
| **Mif2-2** | L - y5+ | 765.4042 | Q - y4+ | 652.3202 | 0.58 |
| **Cbf2** | N - y9+ | 989.4283 | V - y10+ | 1088.4967 | 1.17 |
| **Mcm21** | D - b3+ | 344.1452 | D - y7+ | 793.3686 | 2.09 |
| **Ctf19** | D - y6+ | 747.3268 | S - y9+ | 1060.5269 | 2.01 |
| **Ctf3** | G - y9+ | 958.5680 | I - y4+ | 529.3457 | 0.98 |
| **Iml3** | T - y4+ | 464.2463 | V - y5+ | 563.3148 | 1.89 |
| **Chl4** | P - y6+ | 734.4196 | D - y10+ | 1122.5426 | 2.74 |
| **Mtw1** | P - y11++ | 676.8277 | L - y7+ | 834.4468 | 1.68 |
| **Cnn1** | A - y2+ | 246.1561 | L - b10+ | 1131.6045 | 2.64 |
| **Nkp1** | E - y3+ | 417.2456 | D - y7+ | 862.3901 | 0.32 |
| **Nkp2** | S - y6+ | 704.3573 | L - y4+ | 488.2827 | 1.72 |
| **Ndc80** | D - y9+ | 1033.5273 | S - y7+ | 831.4683 | 1.47 |
| **Dsn1** | E - y9+ | 1154.4960 | Y - y7+ | 911.4105 | 0.97 |
| **Spc105** | Y - y5+ | 609.2991 | S - y4+ | 446.2358 | 0.78 |
| **Okp1** | Q - y5+ | 666.3206 | A - y4+ | 538.2620 | 1.20 |
| **Ame1** | D - y5+ | 605.3253 | E - y6+ | 734.3679 | 0.81 |
