# Supplementary material for: Concatemer-assisted stoichiometry analysis: targeted mass spectrometry for protein quantification
Source: Life Sci Alliance. 2024 Dec 31;8(3):e202403007. doi: 10.26508/lsa.202403007 (PMC11707388; doi:10.26508/lsa.202403007)
Supplement: Supplementary file 14 [file LSA-2024-03007_TableS7.docx]

## Table S7. Quantifier and qualifier ions (H-CKP).

| **Peptide** | **Transitions** | | | | **Quant/Qual ion ratio** |
| --- | --- | --- | --- | --- | --- |
|  | **Quantifier** | **Quantifier m/z** | **Qualifier** | **Qualifier m/z** |  |
| **Cbf1** | T - y10++ | 598.7745 | S - y11++ | 642.2905 | 1.33 |
| **Cep3** | Y - y5+ | 691.3649 | T - y3+ | 415.2175 | 5.62 |
| **Ctf13** | D - y4+ | 548.2703 | L - b3+ | 272.1605 | 1.10 |
| **Cse4** | A - y6+ | 774.4384 | L - y5+ | 703.4013 | 1.45 |
| **Htb2** | A - b2+ | 209.1033 | V - y6+ | 658.3394 | 1.94 |
| **Hta2** | P - y4+ | 438.2699 | F - y5+ | 585.3383 | 1.19 |
| **Hhf1** | Y - y5+ | 705.3442 | I - y6+ | 818.4282 | 1.19 |
| **Hht1** | E - y5+ | 653.4220 | L - y3+ | 411.2953 | 1.18 |
| **Mif2-1** | S - y5+ | 555.3125 | S - y7+ | 771.3871 | 1.43 |
| **Mif2-2** | L - y5+ | 775.4125 | Q - y4+ | 662.3284 | 0.58 |
| **Cbf2** | N - y9+ | 999.4365 | V - y10+ | 1098.505 | 1.17 |
| **Mcm21** | D - b3+ | 344.1452 | D - y7+ | 803.3769 | 2.09 |
| **Ctf19** | D - y6+ | 757.3350 | S - y9+ | 1070.535 | 2.01 |
| **Ctf3** | G - y9+ | 968.5763 | I - y4+ | 539.3539 | 0.98 |
| **Iml3** | T - y4+ | 474.2546 | V - y5+ | 573.323 | 1.89 |
| **Chl4** | P - y6+ | 744.4278 | D - y10+ | 1132.551 | 2.74 |
| **Mtw1** | P - y11++ | 681.8318 | L - y7+ | 844.4551 | 1.68 |
| **Cnn1** | A - y2+ | 256.1643 | L - b10+ | 1131.604 | 2.64 |
| **Nkp1** | E - y3+ | 427.2539 | D - y7+ | 872.3984 | 0.32 |
| **Nkp2** | S - y6+ | 714.3656 | L - y4+ | 498.291 | 1.72 |
| **Ndc80** | D - y9+ | 1043.5355 | S - y7+ | 841.4766 | 1.47 |
| **Dsn1** | E - y9+ | 1164.5043 | Y - y7+ | 921.4188 | 0.97 |
| **Spc105** | Y - y5+ | 619.3074 | S - y4+ | 456.244 | 0.78 |
| **Okp1** | Q - y5+ | 676.3288 | A - y4+ | 548.2703 | 1.20 |
| **Ame1** | D - y5+ | 615.3336 | E - y6+ | 744.3762 | 0.81 |
