# Supplementary material for: Concatemer-assisted stoichiometry analysis: targeted mass spectrometry for protein quantification
Source: Life Sci Alliance. 2024 Dec 31;8(3):e202403007. doi: 10.26508/lsa.202403007 (PMC11707388; doi:10.26508/lsa.202403007)
Supplement: Supplementary file 18 [file LSA-2024-03007_TableS8.docx]

## Table S8. Precision (%CV) of process replicates (n = 3) of *ex vivo* kinetochore reconstitutions.

|  | **%CV** | | | | |
| --- | --- | --- | --- | --- | --- |
|  | **M** | | | **G1** | |
| **Peptide** | **ARS** | **CEN** | **MUT** | **CEN** | **MUT** |
| **Ame1** | 19.8 | 3.7 | 21.3 | 24.4 | 30.2 |
| **Cbf1** | 78.0 | 13.3 | 10.4 | 11.7 | 23.4 |
| **Cbf2** | 6.2 | 4.4 | 20.4 | 34.4 |  |
| **Cep3** | 15.4 | 19.4 | 10.3 | 26.8 | 50.5 |
| **Chl4** |  | 6.2 |  | 42.2 |  |
| **Cnn1** |  | 4.4 |  |  |  |
| **Cse4** | 5.2 | 7.2 | 6.3 | 43.5 |  |
| **Ctf13** | 12.8 | 8.8 | 30.4 | 37.1 |  |
| **Ctf19** | 9.6 | 12.9 | 12.0 | 8.9 | 18.9 |
| **Ctf3** |  | 13.8 | 20.9 | 53.9 | 31.4 |
| **Dsn1** | 4.0 | 0.7 | 4.7 | 5.6 | 9.5 |
| **Hhf1** | 10.8 | 10.6 | 17.9 | 28.8 | 44.3 |
| **Hht1** | 12.5 | 6.3 | 16.9 | 28.1 | 34.1 |
| **Hta2** | 5.7 | 3.1 | 10.4 | 34.9 | 14.4 |
| **Htb2** | 6.7 | 7.3 | 19.9 | 37.1 | 22.5 |
| **Iml3** |  | 15.5 | 23.6 | 36.6 | 41.2 |
| **Mcm21** |  | 5.0 | 26.9 | 37.2 | 29.0 |
| **Mif2-1** |  | 8.6 |  |  |  |
| **Mif2-2** | 16.5 | 12.5 |  | 36.8 |  |
| **Mtw1** | 10.4 | 4.2 | 11.4 | 18.4 | 36.1 |
| **Ndc80** |  | 17.9 |  |  |  |
| **Nkp1** |  | 1.4 |  |  |  |
| **Nkp2** |  | 40.8 |  |  |  |
| **Okp1** |  | 9.1 | 9.8 | 31.9 | 24.2 |
| **Spc105** |  |  |  |  |  |

%CVs were not calculated for measurements < lower limit of quantification, which are shown as empty cells in the table above.
