# Supplementary material for: Concatemer-assisted stoichiometry analysis: targeted mass spectrometry for protein quantification
Source: Life Sci Alliance. 2024 Dec 31;8(3):e202403007. doi: 10.26508/lsa.202403007 (PMC11707388; doi:10.26508/lsa.202403007)
Supplement: Supplementary file 21 [file LSA-2024-03007_TableS9.docx]

## Table S9. Average XPRESS value (Light/Heavy), %CV of XPRESS values, and identification (ID) counts of analytical replicates (n = 3) for *ex vivo* kinetochore reconstitutions as evaluated by DDA-MS.

| **Peptide** | **Mean XPRESS** | | **%CV of XPRESS** | | **Light ID count** | | **Heavy ID count** | |
| --- | --- | --- | --- | --- | --- | --- | --- | --- |
|  | **CEN** | **ARS** | **CEN** | **ARS** | **CEN** | **ARS** | **CEN** | **ARS** |
| **Ame1** |  |  |  |  |  |  | 3 | 3 |
| **Cbf1** | 17.37 |  | 38.07 |  | 6 |  | 3 | 3 |
| **Cep3** | 8.85 |  | 26.31 |  | 3 |  | 1 |  |
| **Chl4** |  |  |  |  |  |  | 3 | 3 |
| **Cnn1** | 0.32 |  | 49.06 |  | 2 |  | 6 | 3 |
| **Cse4** |  |  |  |  |  |  | 5 | 3 |
| **Ctf13** |  |  |  |  |  |  | 2 | 3 |
| **Ctf19** |  |  |  |  |  |  | 3 | 3 |
| **Ctf3** |  |  |  |  |  |  | 3 |  |
| **Dsn1** |  |  |  |  |  |  |  |  |
| **Hhf1** | 22.74 |  | 26.59 |  | 5 |  | 2 | 2 |
| **Hht1** | 26.41 |  | 23.37 |  | 2 |  | 3 | 3 |
| **Hta2** |  |  |  |  |  |  | 3 | 3 |
| **Htb2** | 34.03 |  | 25.67 |  | 3 |  | 2 | 2 |
| **Iml3** |  |  |  |  |  |  | 1 | 3 |
| **Mcm21** | 0.48 |  | 27.69 |  | 3 |  | 3 | 3 |
| **Mif2-1** |  |  |  |  |  |  | 3 | 3 |
| **Mif2-2** |  |  |  |  |  |  | 3 | 3 |
| **Mtw1** |  |  |  |  |  |  | 1 | 3 |
| **Ndc80** |  |  |  |  |  |  | 3 | 3 |
| **Nkp1** | 0.34 |  | 25.17 |  | 3 |  | 3 | 3 |
| **Nkp2** |  |  |  |  |  |  | 3 | 3 |
| **Okp1** |  |  |  |  |  |  | 1 | 1 |
| **Spc105** |  |  |  |  |  |  |  |  |

%CVs were not calculated for peptides with no light peptide IDs or total ID counts (# of light IDs + # of heavy IDs) < 3, shown as empty cells.

Mean XPRESS values were not calculated for peptides with no light peptide IDs, shown as empty cells.
