# Supplementary material for: Concatemer-assisted stoichiometry analysis: targeted mass spectrometry for protein quantification
Source: Life Sci Alliance. 2024 Dec 31;8(3):e202403007. doi: 10.26508/lsa.202403007 (PMC11707388; doi:10.26508/lsa.202403007)
Supplement: Supplementary file 23 [file LSA-2024-03007_TableS10.docx]

## Table S10. Plasmids and yeast strains.

| **Plasmid** | **Description/source** |
| --- | --- |
| HZE2029 | Lic-2GT from Corbett Lab |
| HZE3236 | pRS423-pGAL-GST-tev-HF |
| HZE3240 | Addgene 30116, pFastBacCEN3e2_Plus60_tetO2, from Hinshaw: pSMH1717 |
| HZE3241 | Addgene 30116, pFastBac LIC cloning vector, CEN3e2_Plus60-TetO2-CCGmut, from Hinshaw: pSMH1726 |
| HZE3246 | CEN3-8xLacO-TRP1, from Biggins: pSB964 |
| HZE3361 | pRS423-pGAL-GST-CKP-HF |

| **Yeast Strain** | **Genotype** |
| --- | --- |
| SCY249 | *ura3-52 leu2-1 trp1-63 his3-200 lys2-Bgl hom3-10 ade2-1 ade8 sml1Δ::TRP arg4Δ MAT a* |
| HZY1029 | *bar1Δ::URA MAT a*, W303 in HZY1077 |
| HZY1077 | *ade2-1 can1-100 his3-11,15 leu2-3,112 trp1-1 ura3-1 RAD5+*, *MAT a*, W303 |
| HZY2347 | *Cdc20-HA-AID::G418 pGPD1-OsTir1::LEU2 bar1Δ::URA Mif2-TAF::HisMX sml1Δ::TRP, MAT a* in SCY249 |
| HZY2461 | *Cdc20-HA-AID::G418 pGPD1-OsTir1::LEU2 bar1Δ::URA Ndc80-TAF::G418 sml1Δ::TRP*, *MAT a* in SCY249 |
| HZY2646 | *Ame1-TAF::HisMX Cdc20-HA-AID::G418 pGPD1-OsTir1::LEU2 bar1Δ::URA sml1Δ::TRP, MAT a* in SCY249 |
| HZY2777 | *G418::3xFlag-Cse4 Cdc20-HA-AID::HisMX pGPD1-OsTir1::LEU2 bar1Δ::URA, sml1Δ::TRP*, *MAT a* in SCY249 |
| HZY3059 | HZE3361 in SCY249 |
