# Supplementary material for: Concatemer-assisted stoichiometry analysis: targeted mass spectrometry for protein quantification
Source: Life Sci Alliance. 2024 Dec 31;8(3):e202403007. doi: 10.26508/lsa.202403007 (PMC11707388; doi:10.26508/lsa.202403007)
Supplement: Supplementary file 28 [file LSA-2024-03007_TableS12.docx]

## Table S12. Quantification of light and heavy CKP concentrations using a GST peptide standard.

| **Samples Analyzed** | **Synthetic GST peptide: YGVSR** | | | | | | **Light CKP** | | | **Heavy CKP** | | |
| --- | --- | --- | --- | --- | --- | --- | --- | --- | --- | --- | --- | --- |
| **Cal. Levels/**  **Repeat** | **1** | **2** | **3** | **4** | **5** | **6** | **1** | **2** | **3** | **1** | **2** | **3** |
| **Conc. (pM)** | 625 | 1250 | 2500 | 5000 | 10000 | 20000 | 14491 | 13881 | 13564 | 3073.3 | 3302.9 | 3427.6 |
| **Peak area (10^3^)** | 2567 | 5746 | 10155 | 23086 | 38492 | 82090 | 59331 | 56842 | 55550 | 12804 | 13740 | 14247 |
| **Avg. conc. (pM)** |  |  |  |  |  |  | 13978.67 | | | 3267.93 | | |
| **Avg. peak area (10^3^)** |  |  |  |  |  |  | 57241 | | | 13597 | | |
| **Std. dev conc.** |  |  |  |  |  |  | 471.15 | | | 179.72 | | |
| **Std. dev peak area (10^3^)** |  |  |  |  |  |  | 1921 | | | 732 | | |
