# Supplementary material for: Concatemer-assisted stoichiometry analysis: targeted mass spectrometry for protein quantification
Source: Life Sci Alliance. 2024 Dec 31;8(3):e202403007. doi: 10.26508/lsa.202403007 (PMC11707388; doi:10.26508/lsa.202403007)

CKP CE/NCE Optimization

CE/NCE Steps Legend

Mass [m/z]	CS [z]	Comment	CE_1	CE_2	CE_3	CE_4	CE_5	CE_6	CE_7	NCE_1	NCE_2	NCE_3	NCE_4	NCE_5	NCE_6	NCE_7	Set
482.222528	2	AME1_NDEDLTTR_light	10	12	14	16	18	20	22	10	12	14	16	18	20	22	1102
787.358073	2	CBF2_EENIVNEDGPNTSR_light	19	21	23	25	27	29	31	10	12	14	16	18	20	22	1102
683.317688	2	CHL4_NEDSGEPVYISR_light	14	16	18	20	22	24	26	10	12	14	16	18	20	22	1102
688.880258	2	CNN1_SFLQDLSQVLAR_light	10	12	14	16	18	20	22	10	12	14	16	18	20	22	1102
727.879924	2	CSE4_YTPSELALYEIR_light	17	19	21	23	25	27	29	21	23	25	27	29	31	33	1102
428.717216	2	HTB2_HAVSEGTR_light	12	14	16	18	20	22	24	18	20	22	24	26	28	30	1102
511.24346	2	MCM21_IDDISTSDR_light	10	12	14	16	18	20	22	10	12	14	16	18	20	22	1102
516.784901	2	MIF2_VAPLQYWR_light	10	12	14	16	18	20	22	10	12	14	16	18	20	22	1102
733.369723	2	MTW1_IPEEYLDANVFR_light	17	19	21	23	25	27	29	10	12	14	16	18	20	22	1102
634.293681	2	NKP1_EIYDNESELR_light	10	12	14	16	18	20	22	10	12	14	16	18	20	22	1102
452.740356	2	NKP2_VTSELEAR_light	10	12	14	16	18	20	22	10	12	14	16	18	20	22	1102
506.582949	3	SPC105_VHISTQQDYSPSR_light	10	12	14	16	18	20	22	16	18	20	22	24	26	28	1102
855.894488	2	DSN1_ILDNTENYDDTELR_light	20	22	24	26	28	30	32	16	18	20	22	24	26	28	1107
291.1557	2	GST_YGVSR_light	10	12	14	16	18	20	22	10	12	14	16	18	20	22	1107
446.740356	2	IML3_ESIVTSTR_light	10	12	14	16	18	20	22	10	12	14	16	18	20	22	1107
662.828223	2	NDC80_QYDSSIQNLTR_light	19	21	23	25	27	29	31	11	13	15	17	19	21	23	1107
462.888038	3	CBF1_LSTEDEEIHSAR_light	10	12	14	16	18	20	22	11	13	15	17	19	21	23	1108
447.258185	2	CEP3_LVYLTER_light	10	12	14	16	18	20	22	10	12	14	16	18	20	22	1108
440.729792	2	CTF13_TGLADFTR_light	10	12	14	16	18	20	22	10	12	14	16	18	20	22	1108
715.367712	2	CTF19_QQLSLLDDDQVR_light	18	20	22	24	26	28	30	10	12	14	16	18	20	22	1108
621.346051	2	CTF3_DAPGSATLILQR_light	10	12	14	16	18	20	22	10	12	14	16	18	20	22	1108
385.7296	2	GST_GLVQPTR_light	10	12	14	16	18	20	22	10	12	14	16	18	20	22	1108
589.82442	2	HHF1_ISGLIYEEVR_light	10	12	14	16	18	20	22	10	12	14	16	18	20	22	1108
416.25036	2	HHT1_STELLIR_light	10	12	14	16	18	20	22	10	12	14	16	18	20	22	1108
459.263802	2	HTA2_AGLTFPVGR_light	10	12	14	16	18	20	22	10	12	14	16	18	20	22	1108
670.820063	2	MIF2_YSLDTSESPSVR_light	18	20	22	24	26	28	30	16	18	20	22	24	26	28	1108
439.740159	2	OKP1_VIQAEYR_light	10	12	14	16	18	20	22	10	12	14	16	18	20	22	1108

CSE4: YTPSELALYEIR

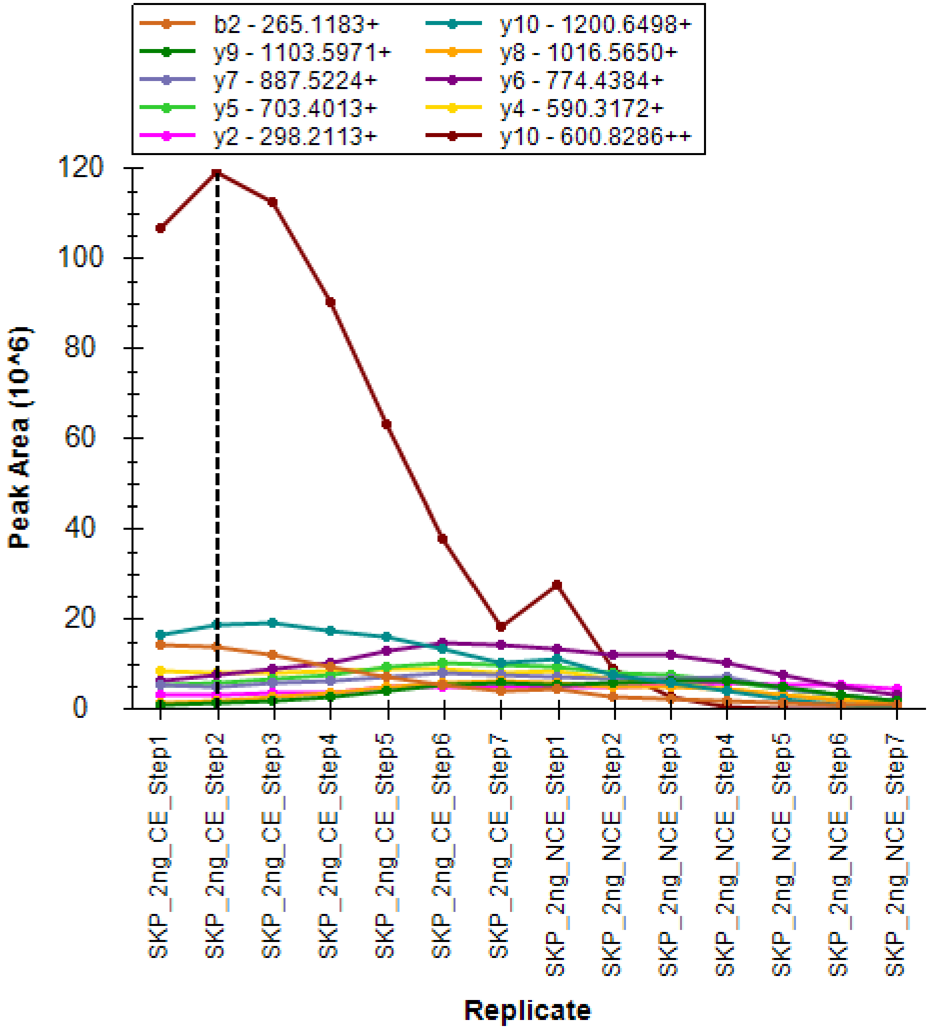

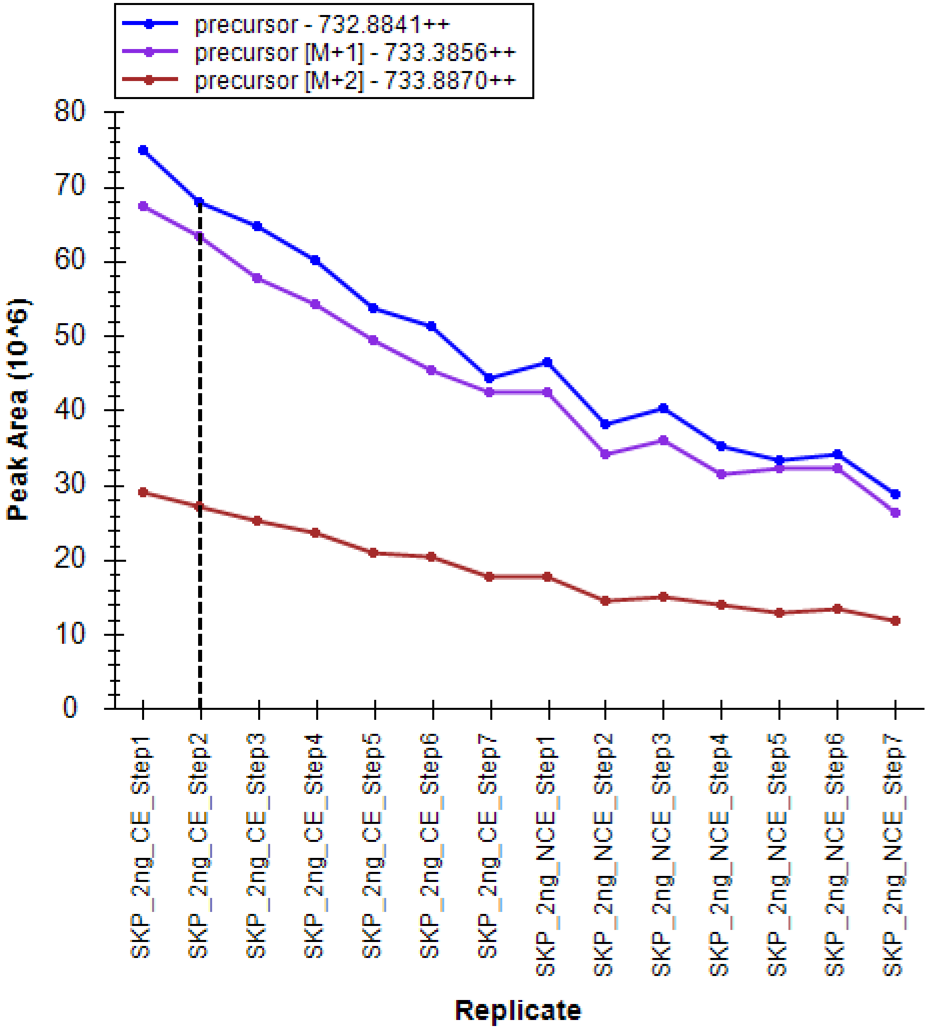

HTB2: HAVSEGTR

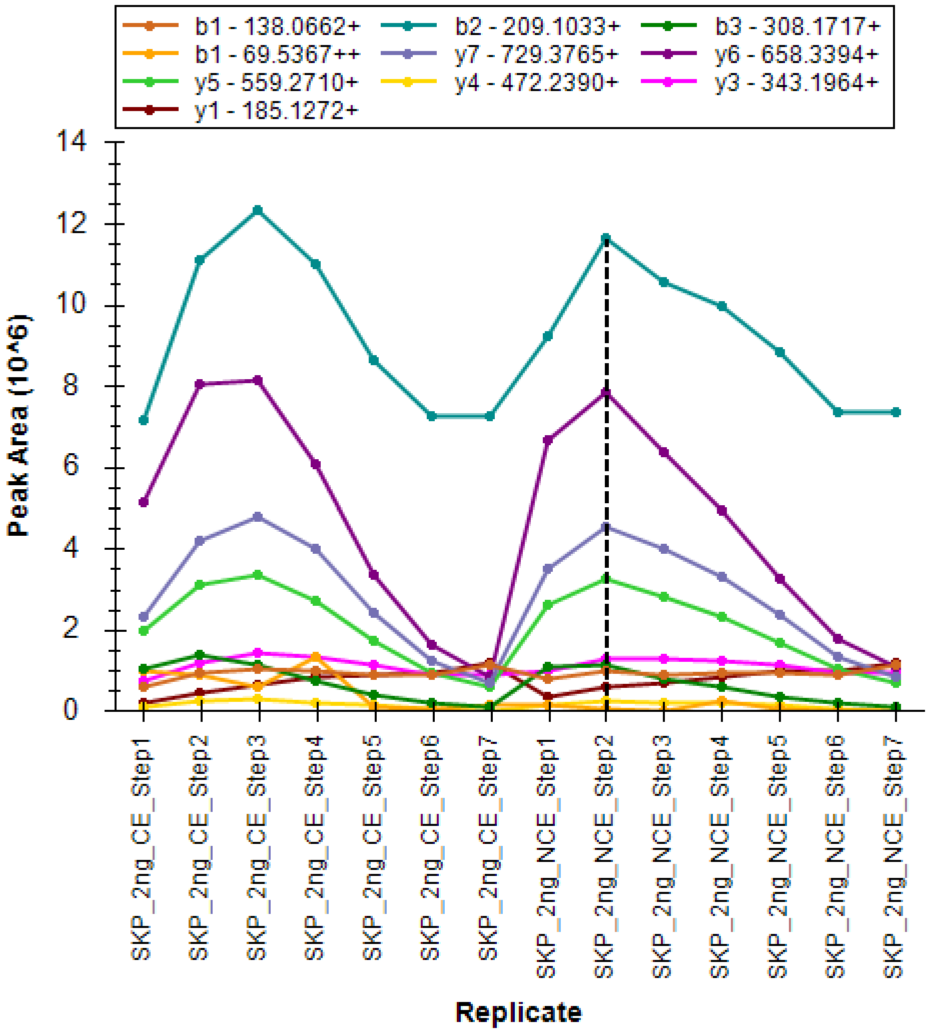

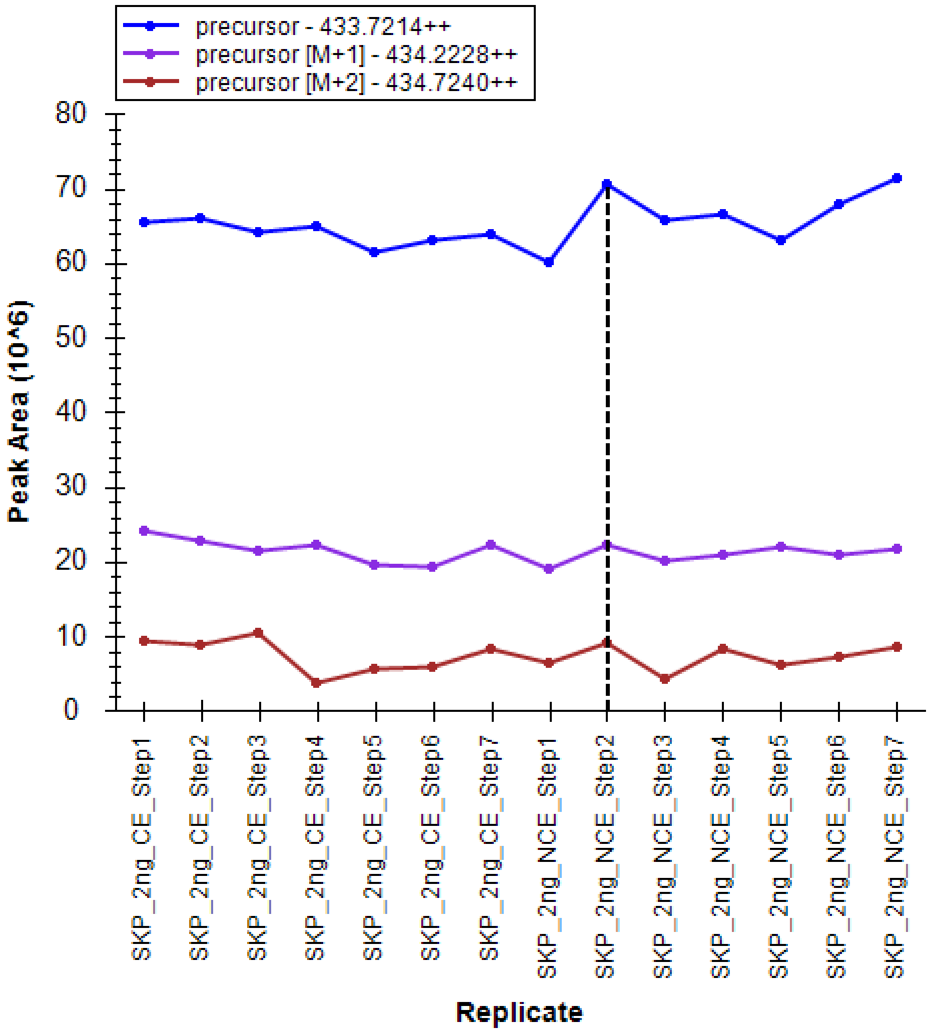

MIF2: VAPLQYWR

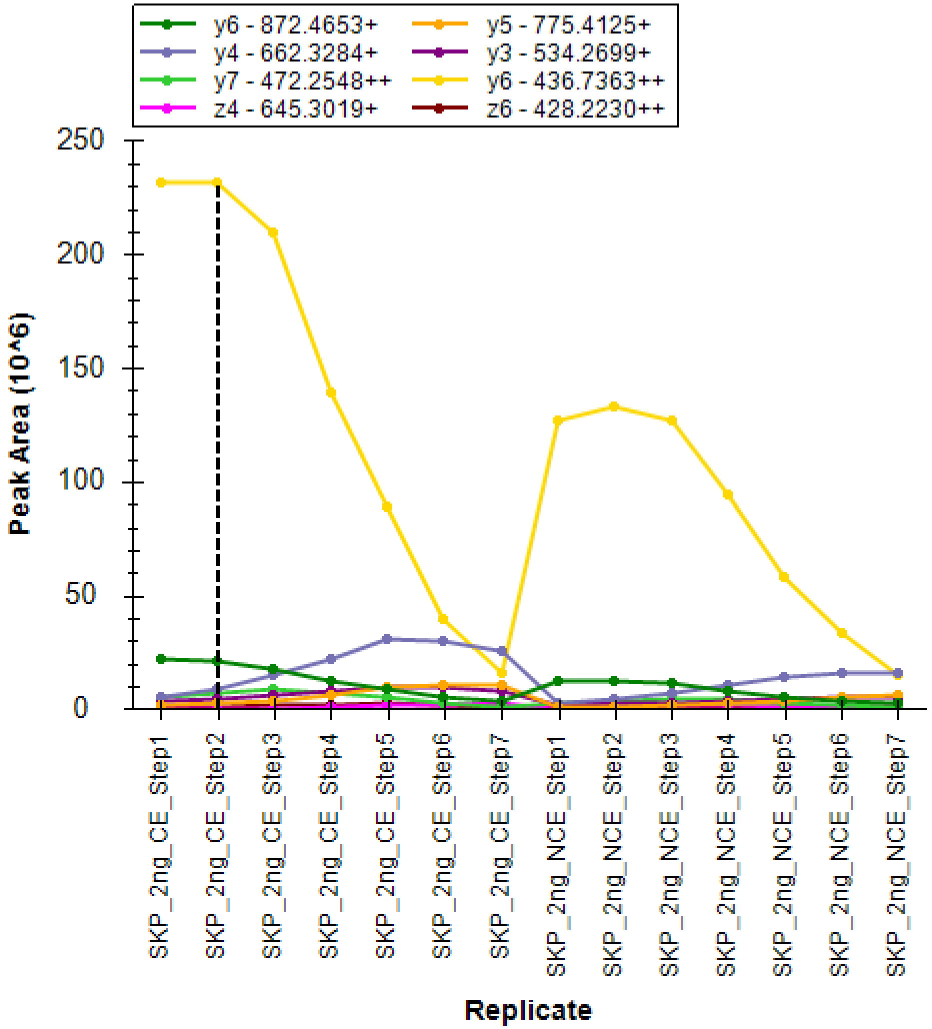

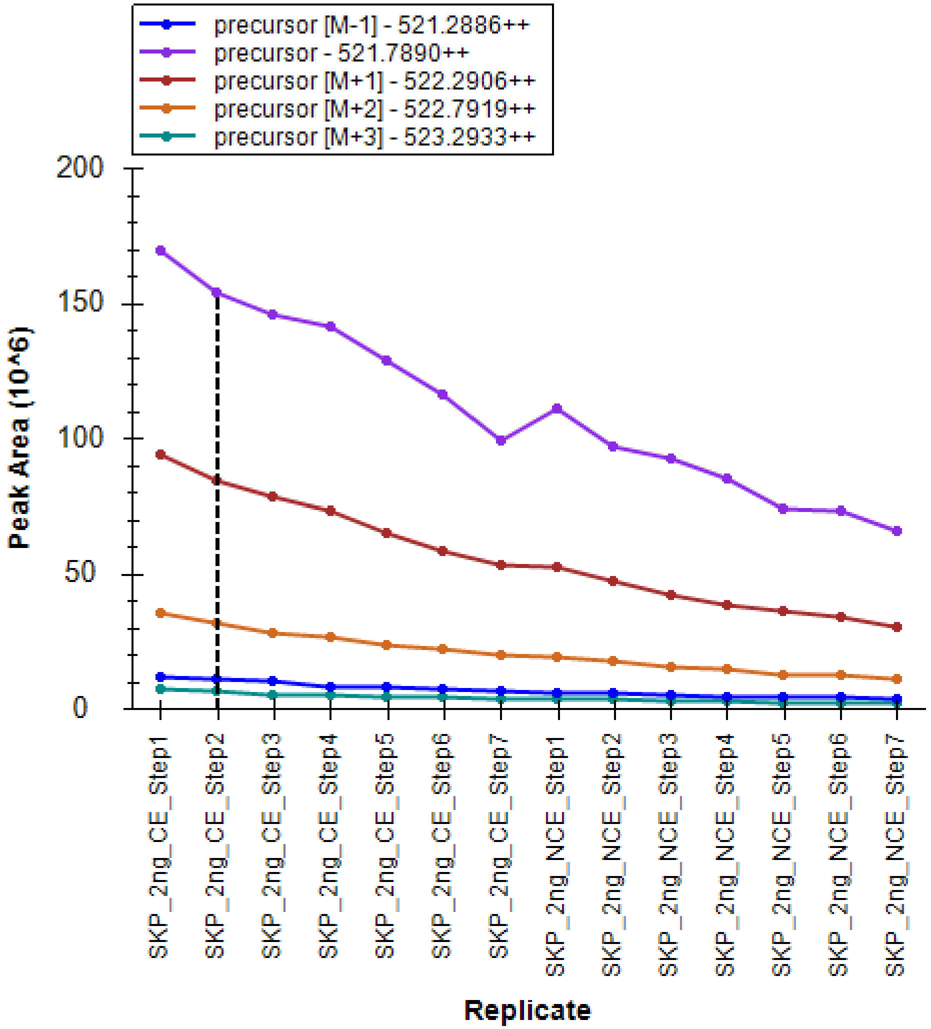

CBF2: EENIVNEDGPNTSR

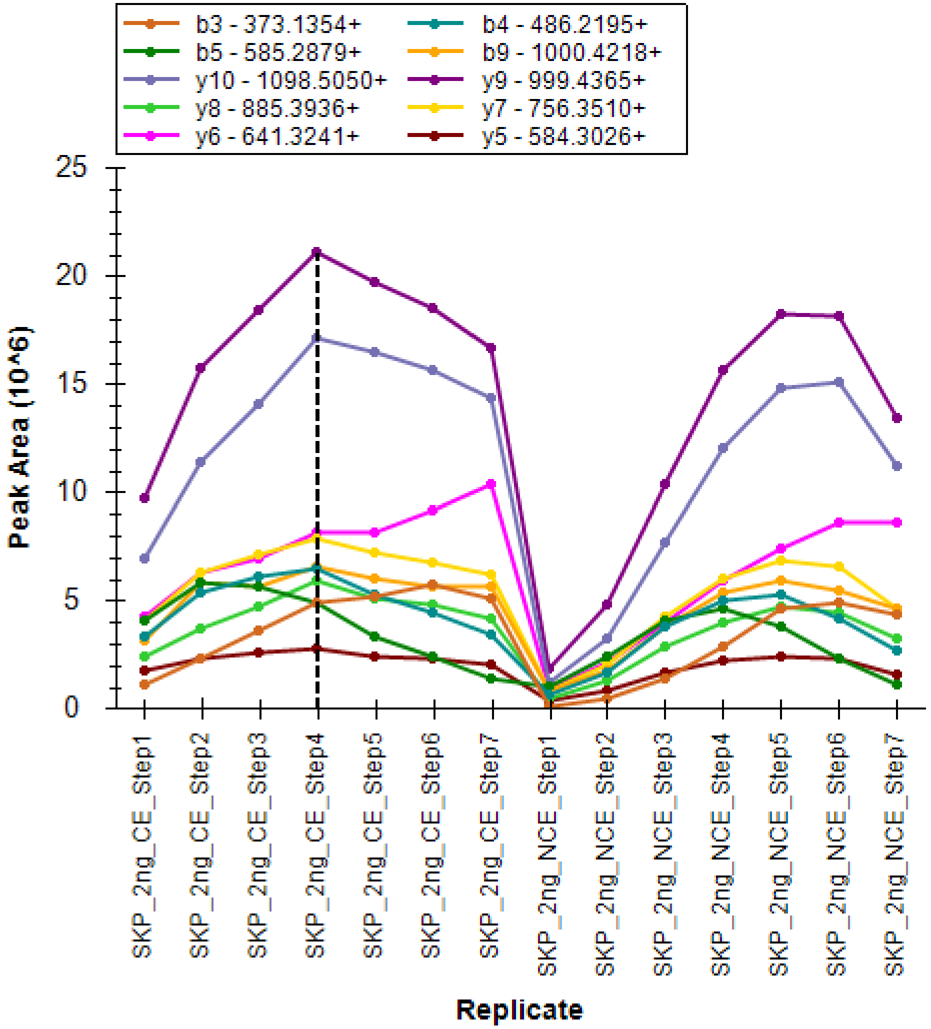

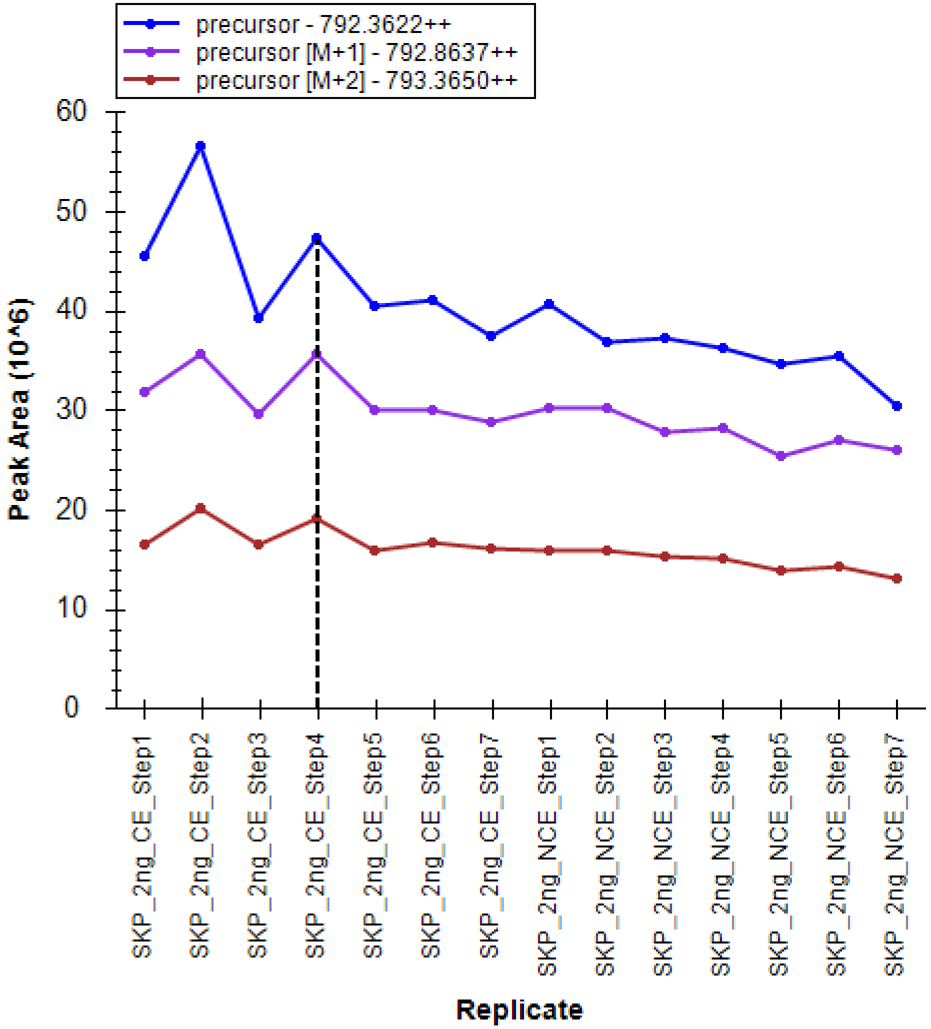

MCM21: IDDISTSDR

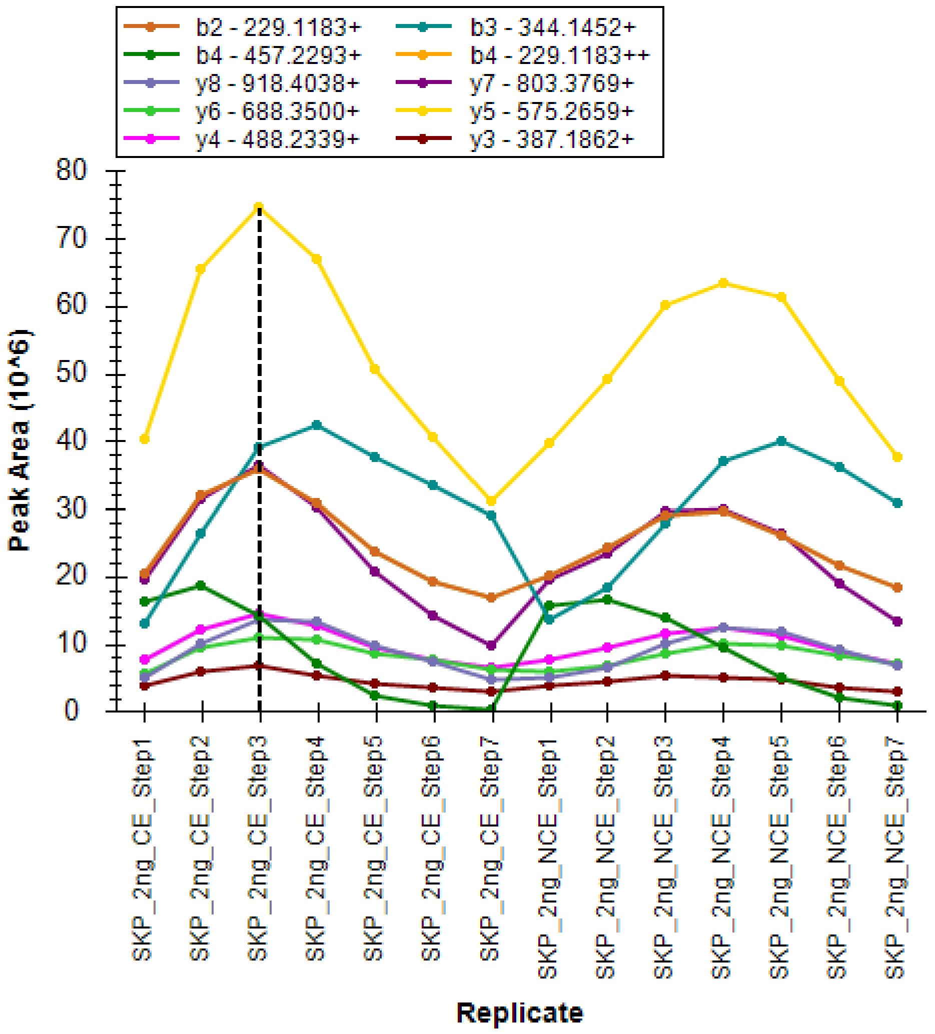

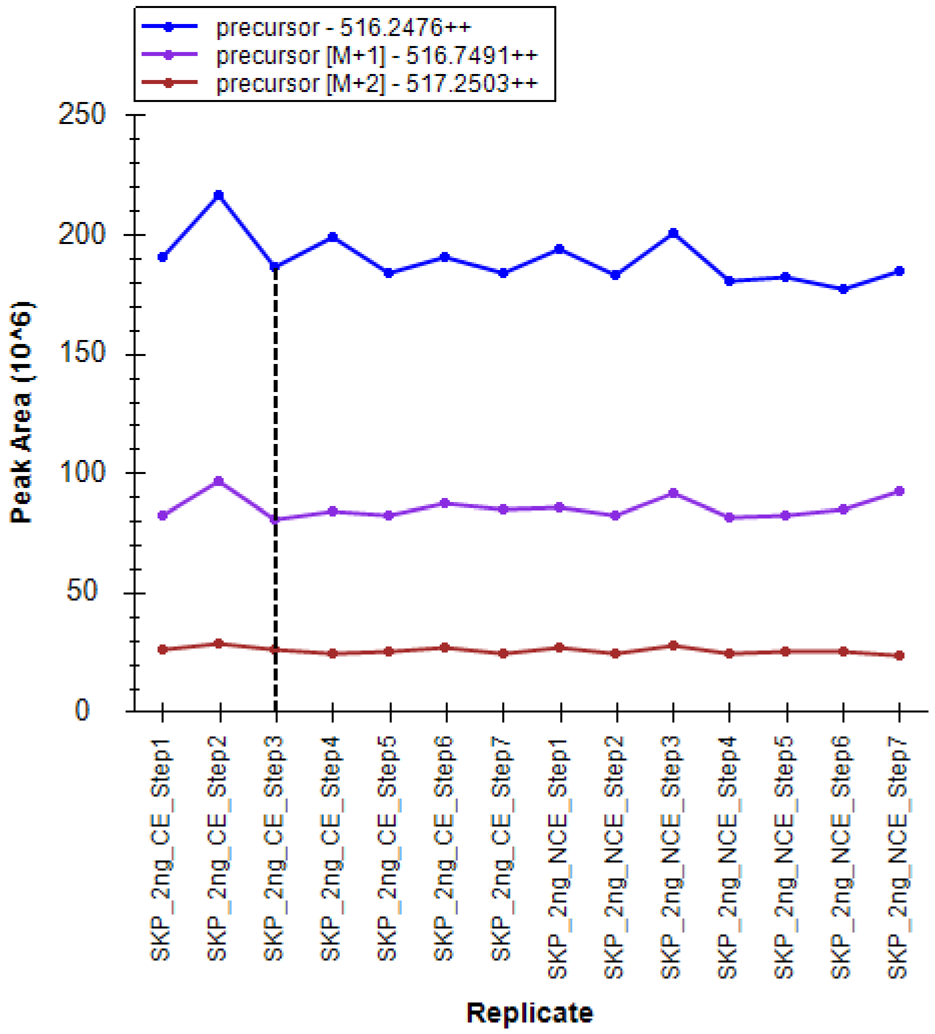

CHL4: NEDSGEPVYISR

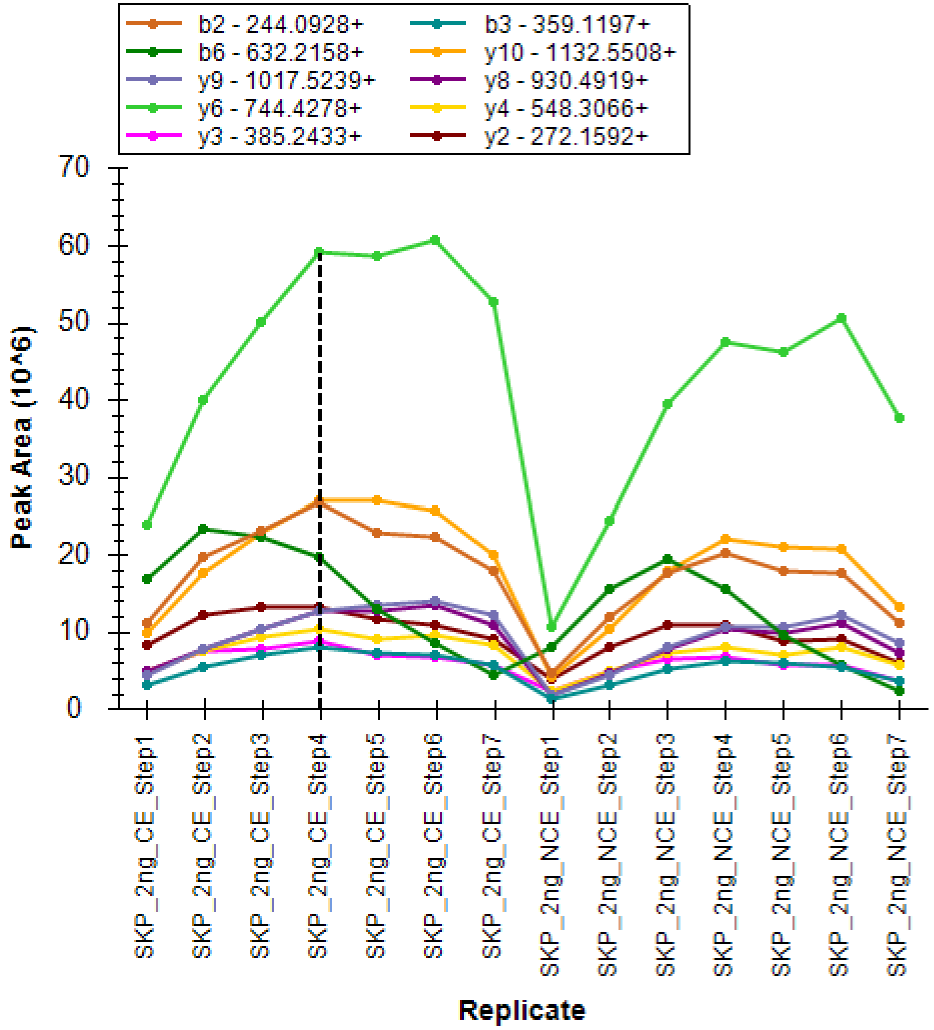

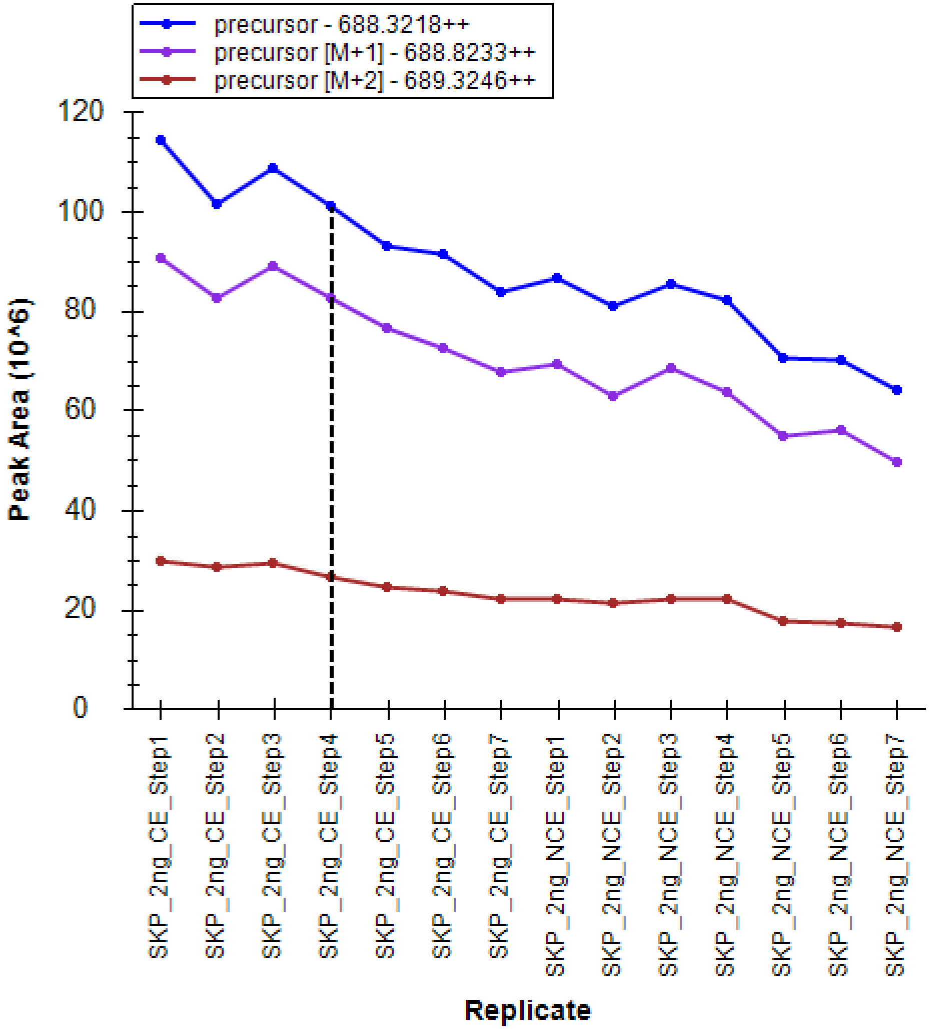

MTW1: IPEEYLDANVFR

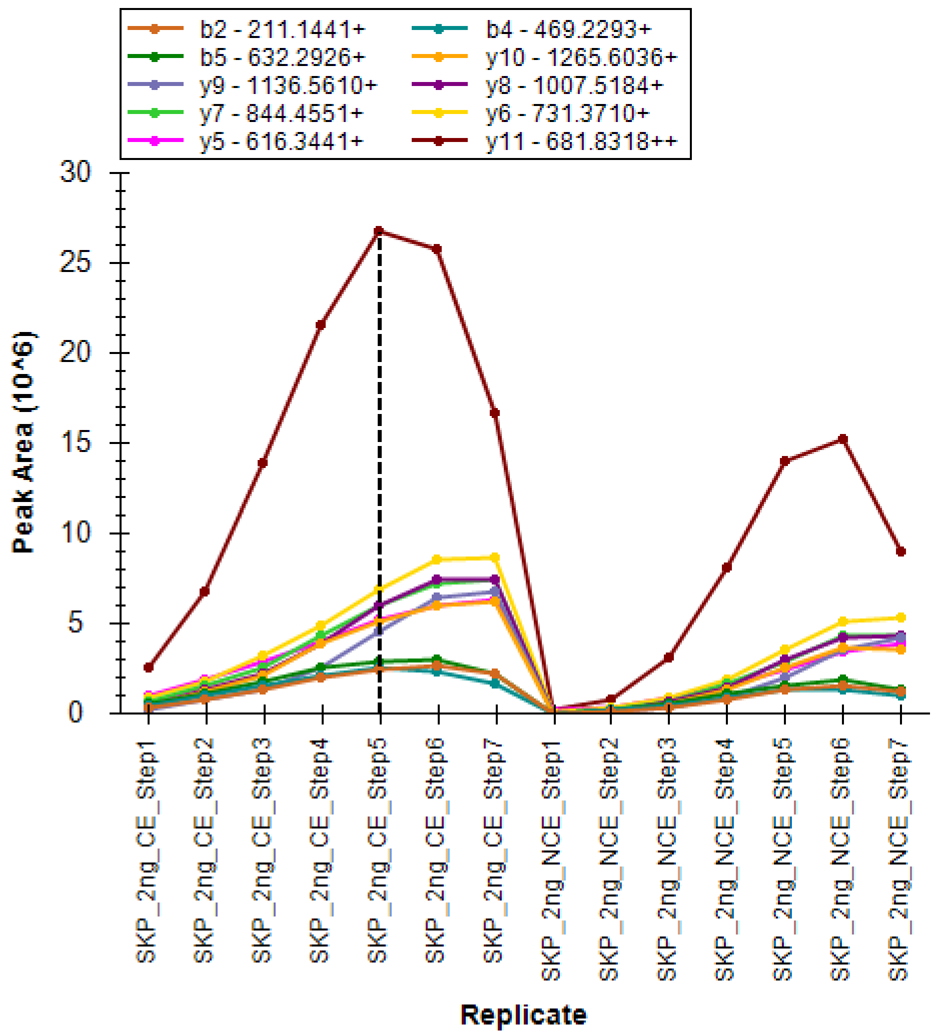

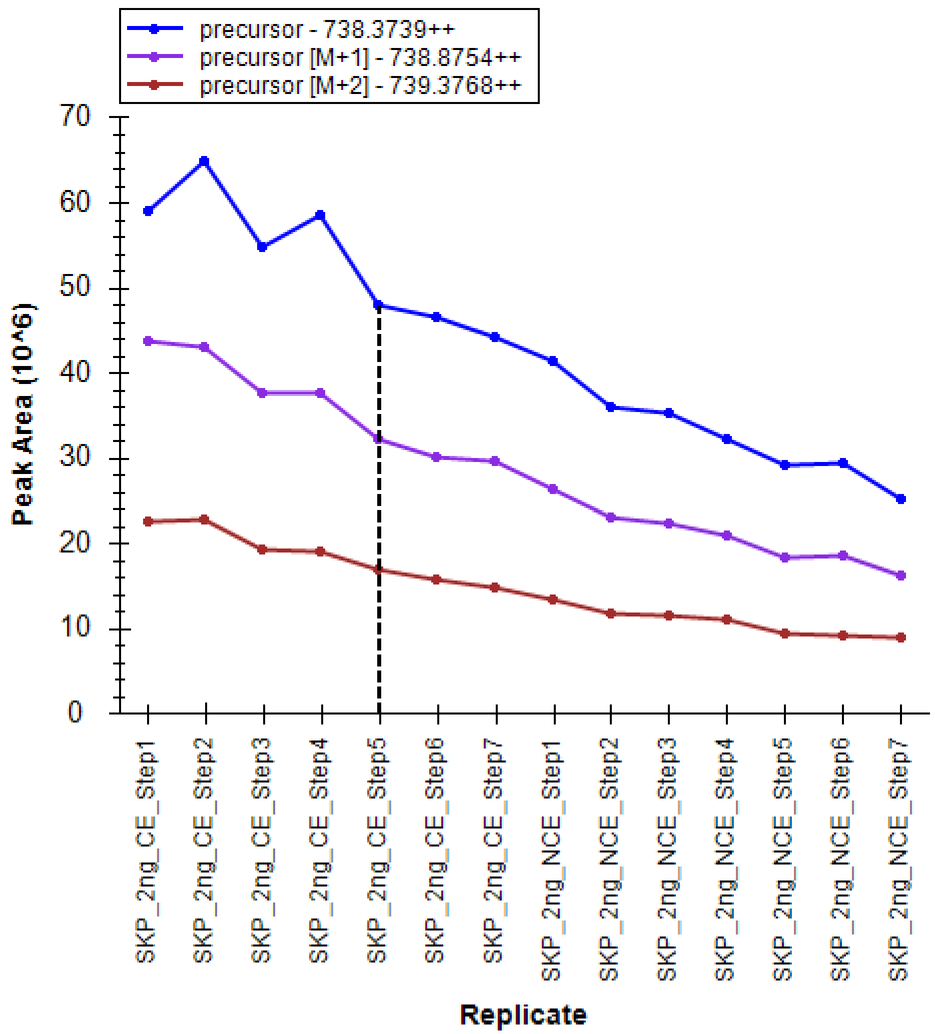

CNN1: SFLQDLSQVLR

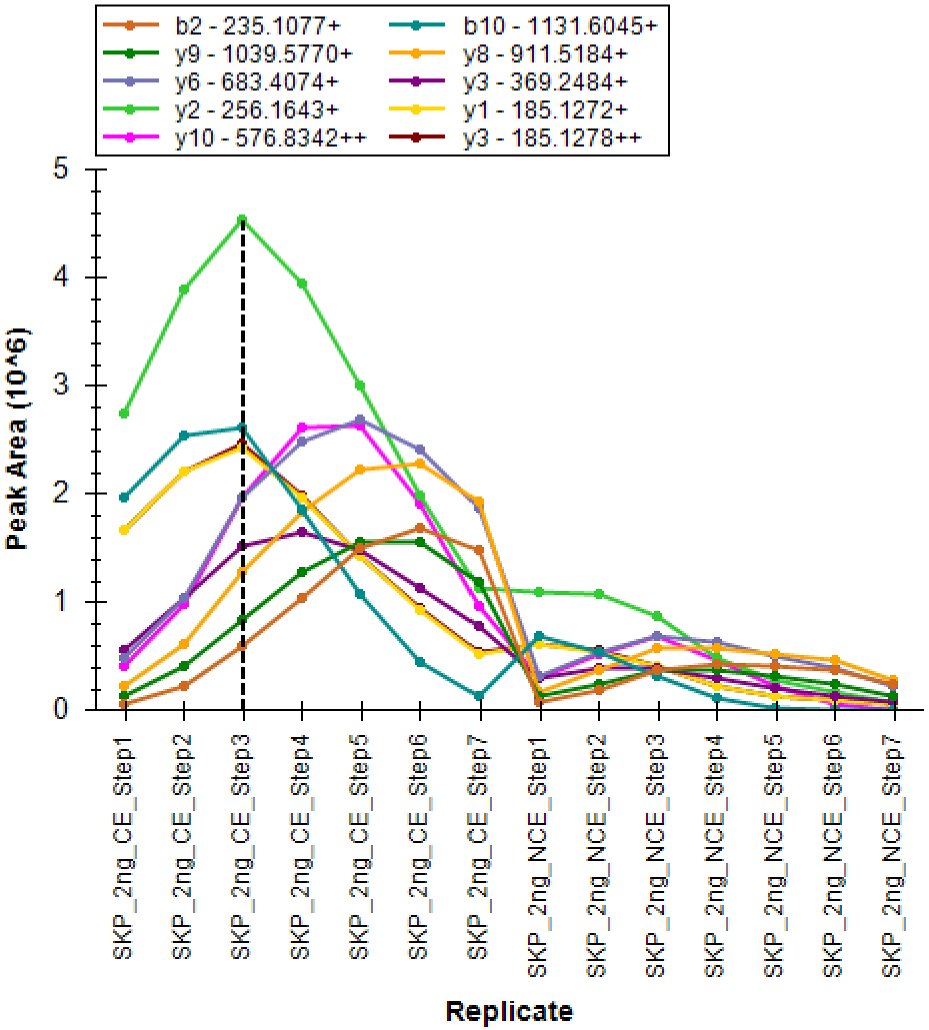

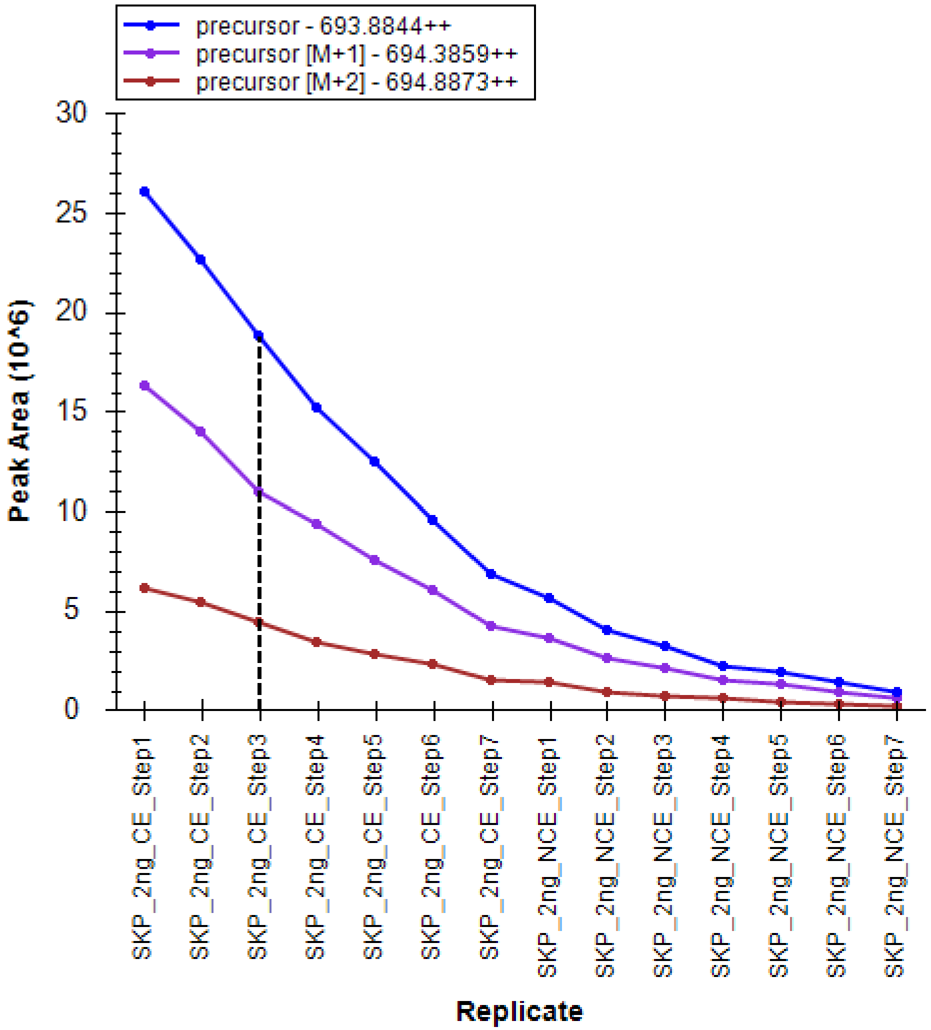

NKP1: EIYDNESELR

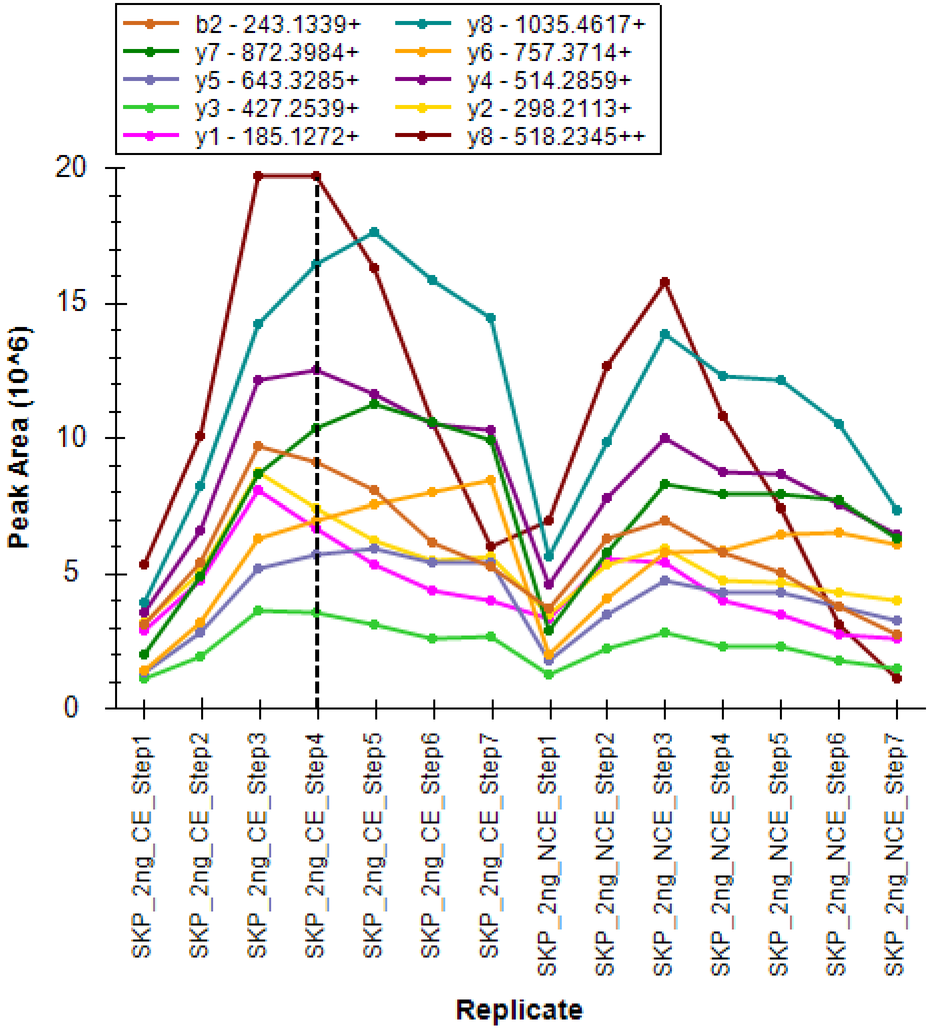

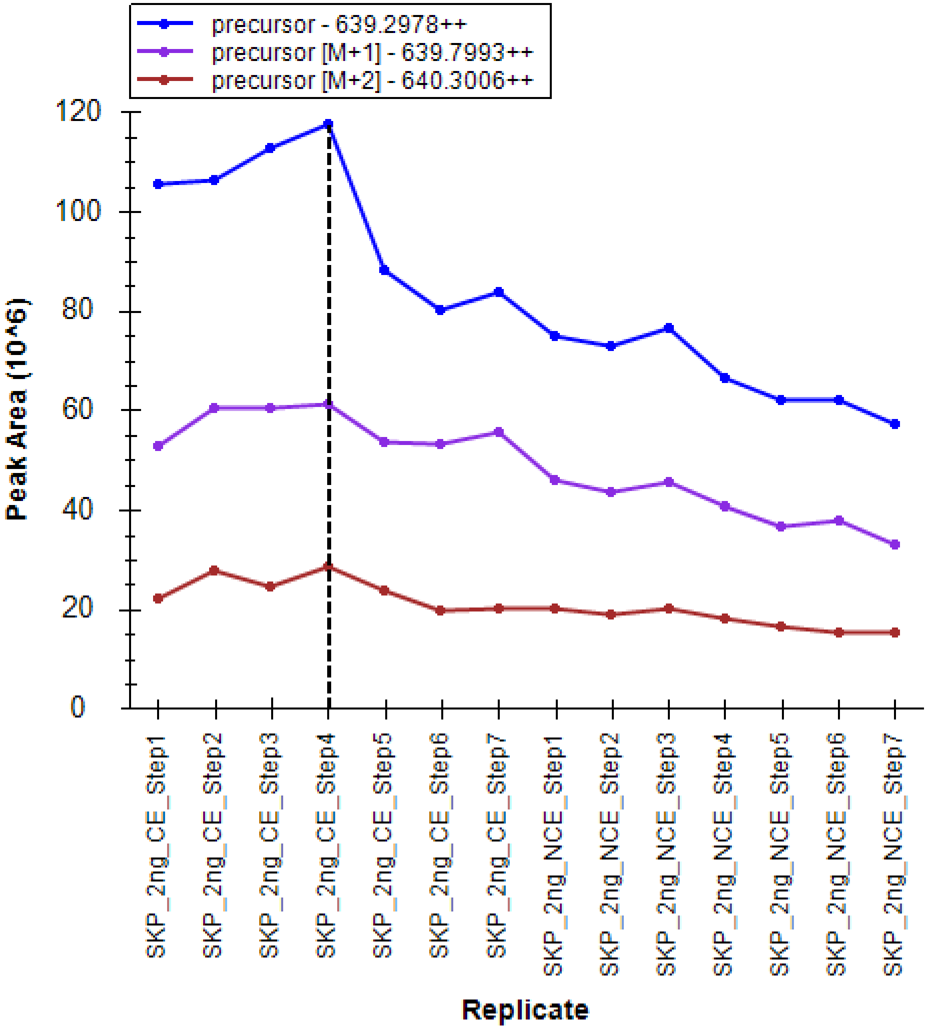

NKP2: VTSELEAR

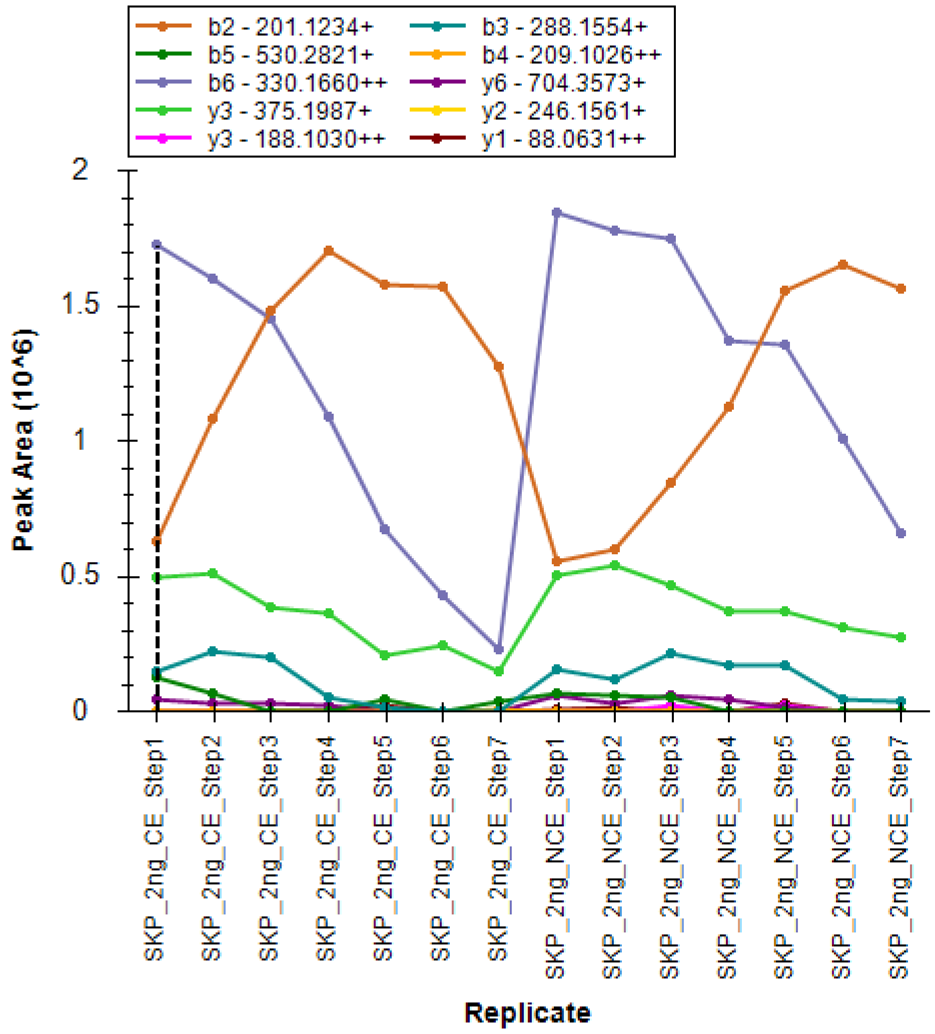

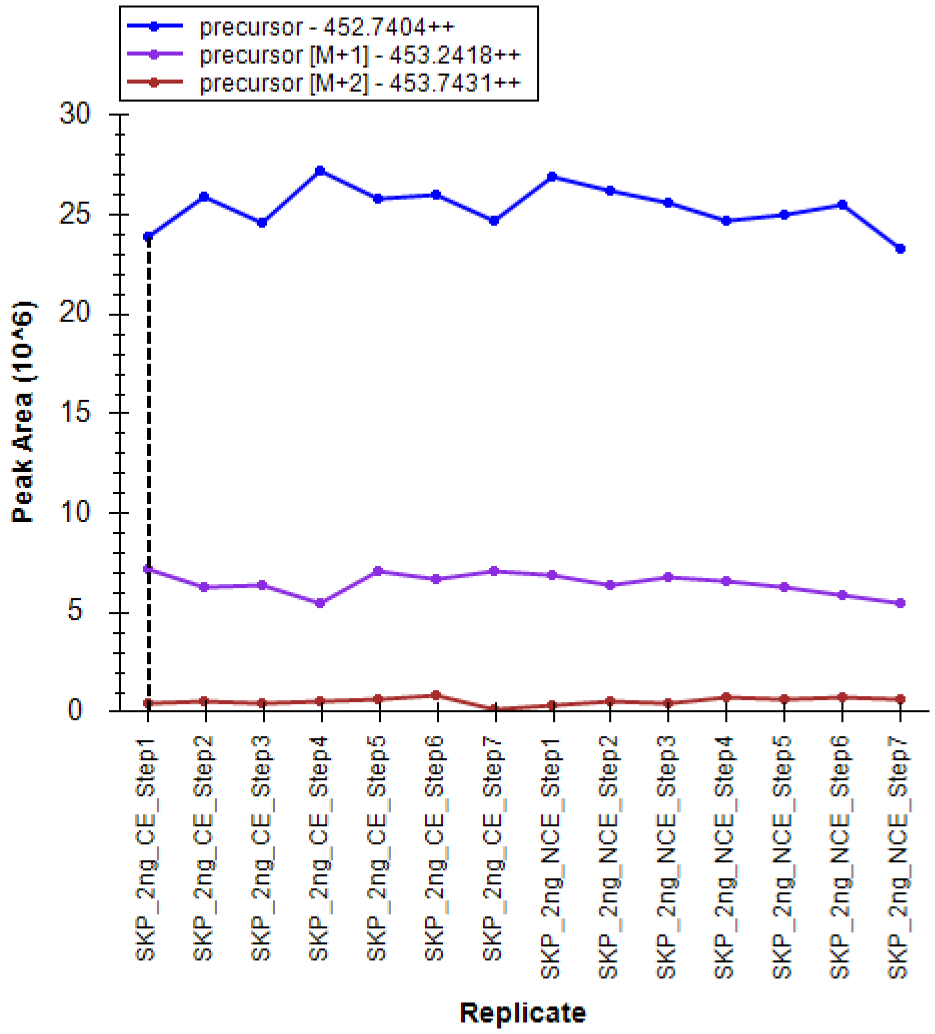

SPC105: VHISTQQDYSPSR

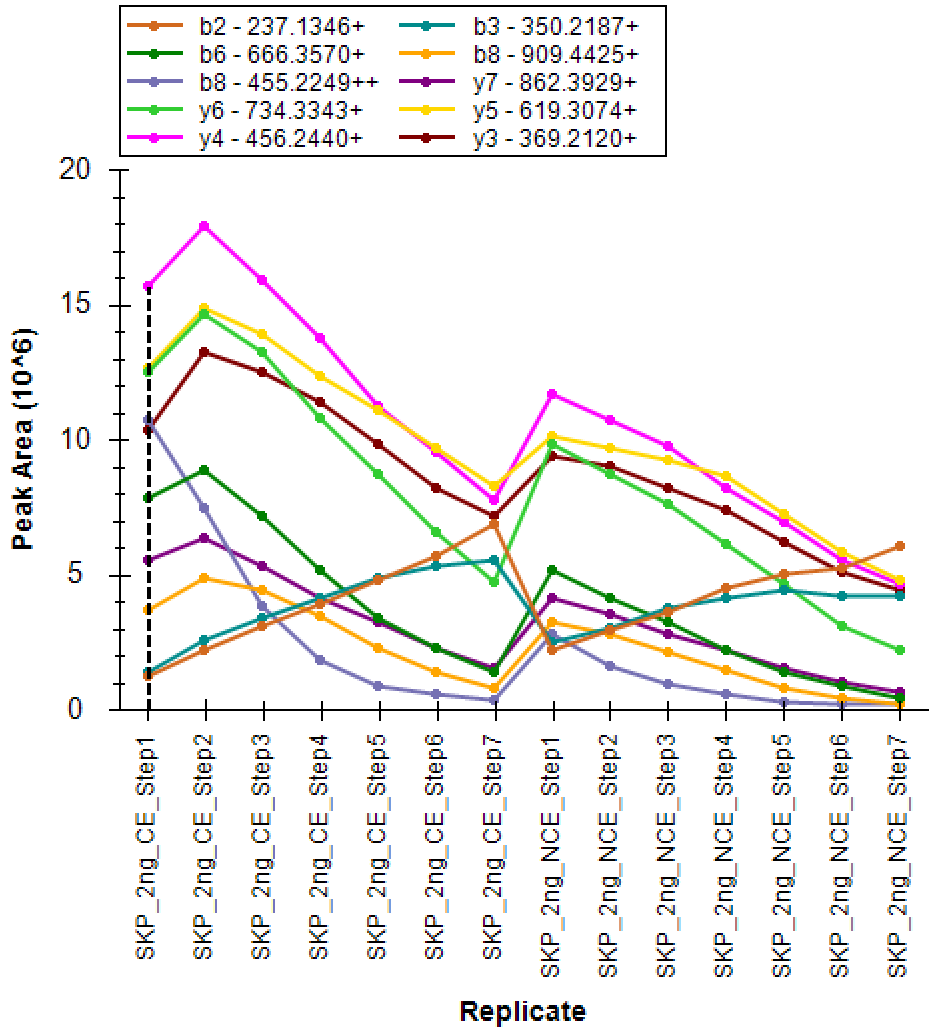

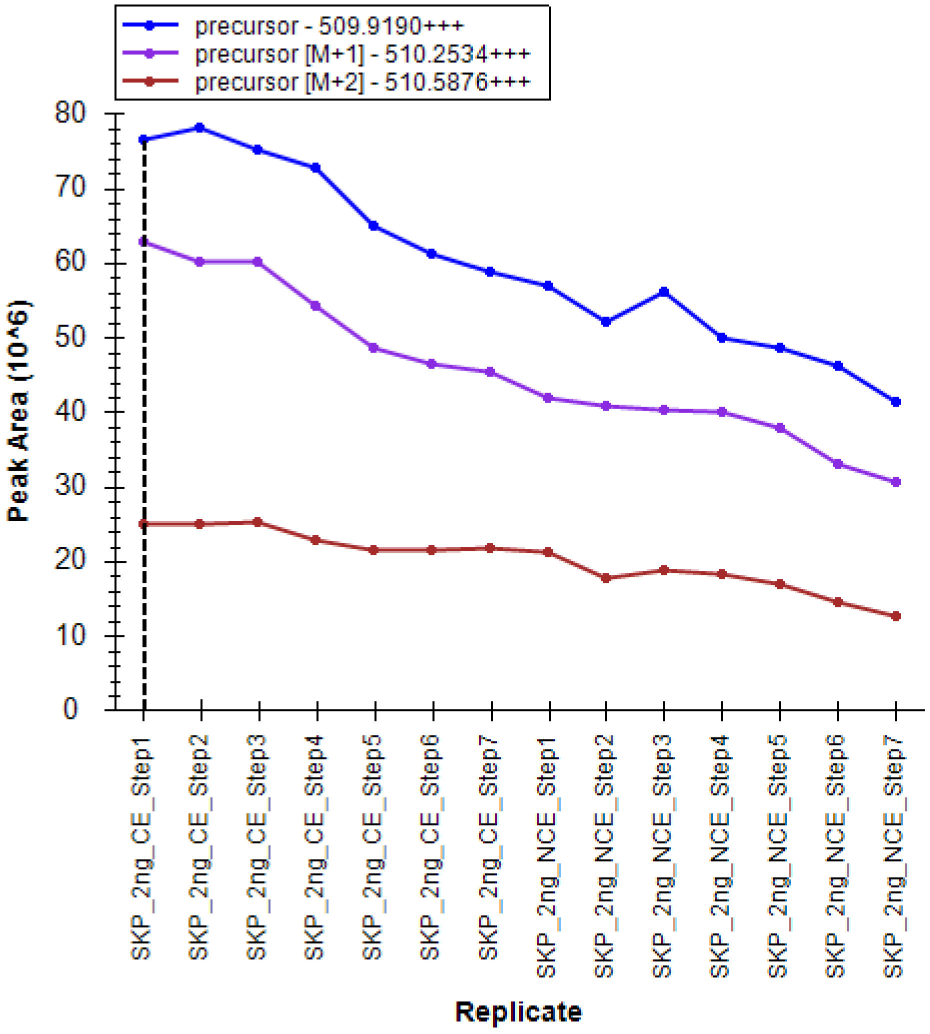

AME1: NDEDLTTR

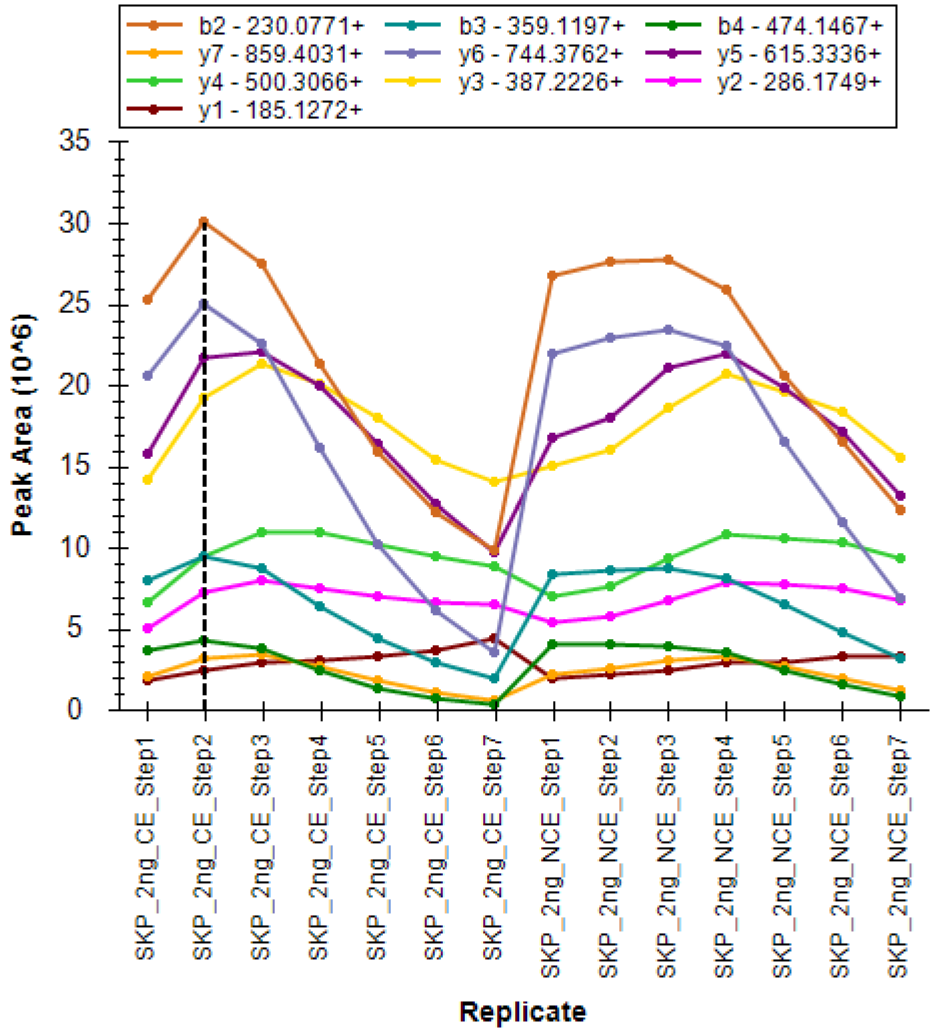

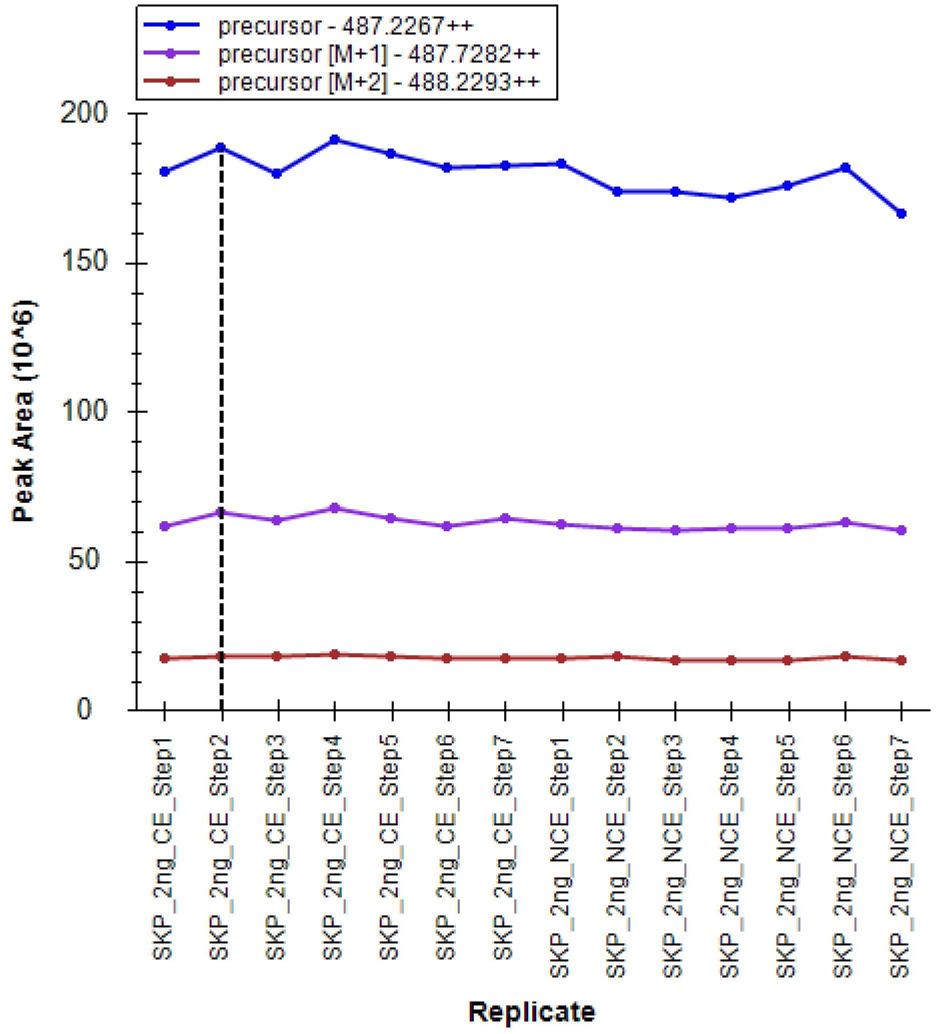

GST-1: YGVSR

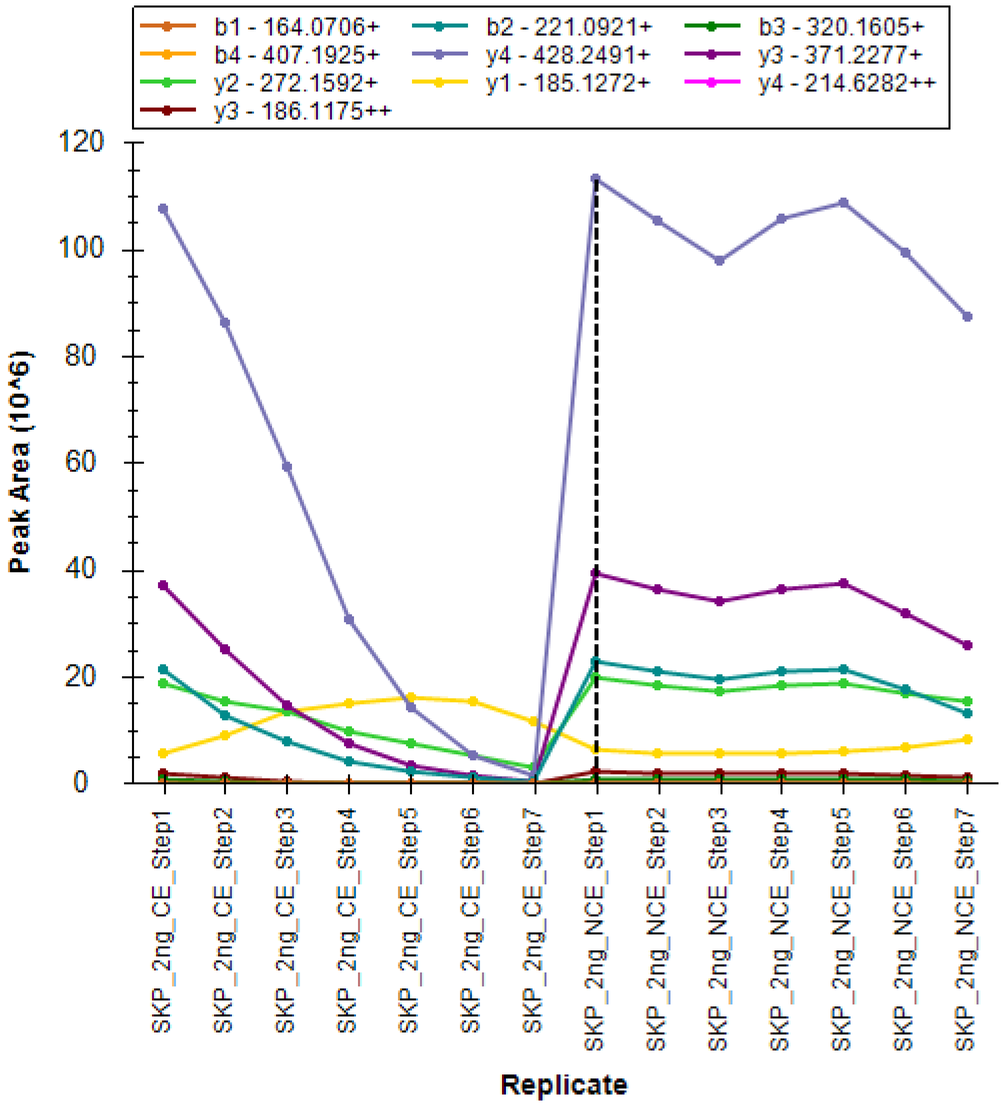
b1 - 164.0706+
b2 - 221.0921+
b3 - 320.1605+
b4 - 407.1925+
y4 - 428.2491+
y3 - 371.2277+
y2 - 272.1592+
y1 - 185.1272+
y4 - 214.6282++
y3 - 186.1175++
Peak Area (10^6)
120
100
80
60
40
20
0
SKP_2ng_CE_Step1
SKP_2ng_CE_Step2
SKP_2ng_CE_Step3
SKP_2ng_CE_Step4
SKP_2ng_CE_Step5
SKP_2ng_CE_Step6
SKP_2ng_CE_Step7
SKP_2ng_NCE_Step1
SKP_2ng_NCE_Step2
SKP_2ng_NCE_Step3
SKP_2ng_NCE_Step4
SKP_2ng_NCE_Step5
SKP_2ng_NCE_Step6
SKP_2ng_NCE_Step7
Replicate

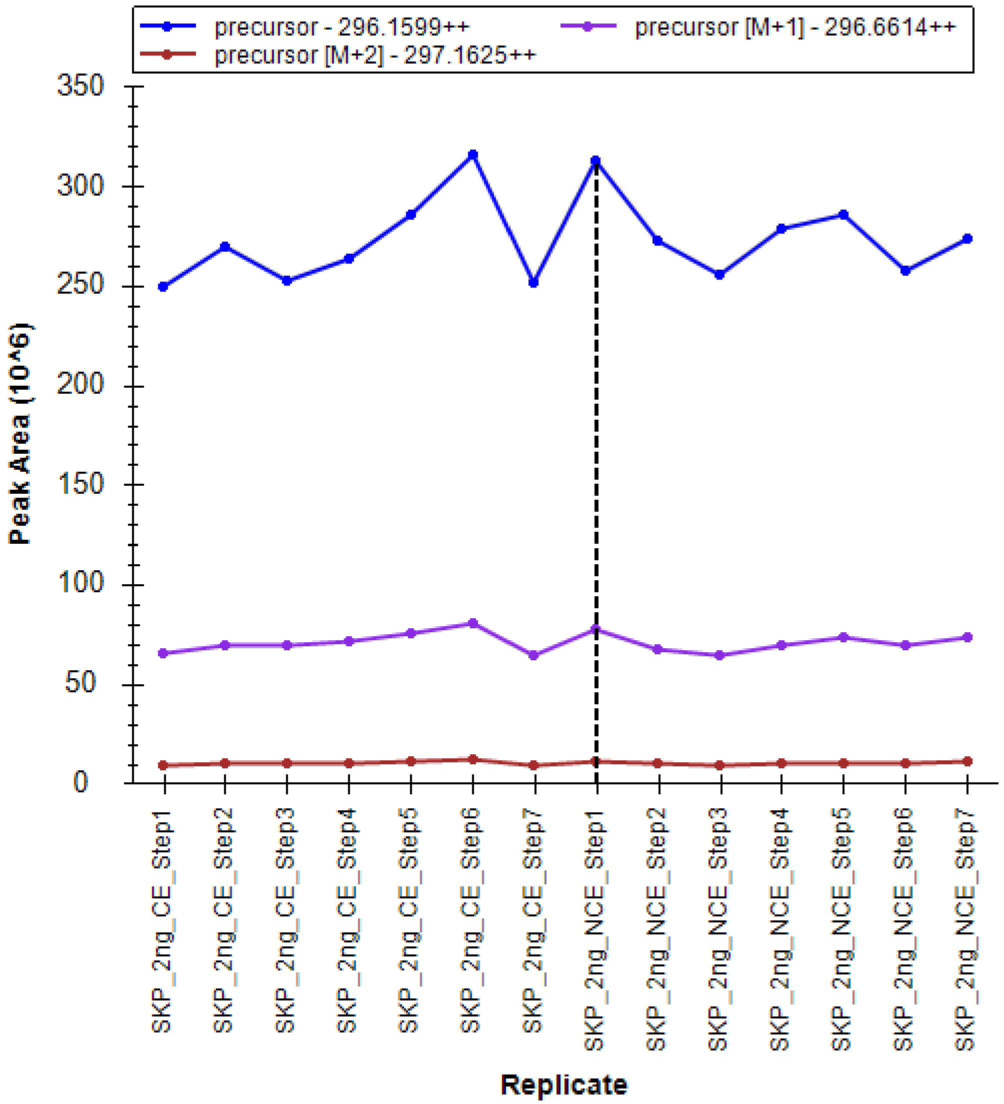
precursor - 296.1599++
precursor [M+1] - 296.6614++
precursor [M+2] - 297.1625++
Peak Area (10^6)
350
300
250
200
150
100
50
0
SKP_2ng_CE_Step1
SKP_2ng_CE_Step2
SKP_2ng_CE_Step3
SKP_2ng_CE_Step4
SKP_2ng_CE_Step5
SKP_2ng_CE_Step6
SKP_2ng_CE_Step7
SKP_2ng_NCE_Step1
SKP_2ng_NCE_Step2
SKP_2ng_NCE_Step3
SKP_2ng_NCE_Step4
SKP_2ng_NCE_Step5
SKP_2ng_NCE_Step6
SKP_2ng_NCE_Step7
Replicate

IML3: ESIVTSTR

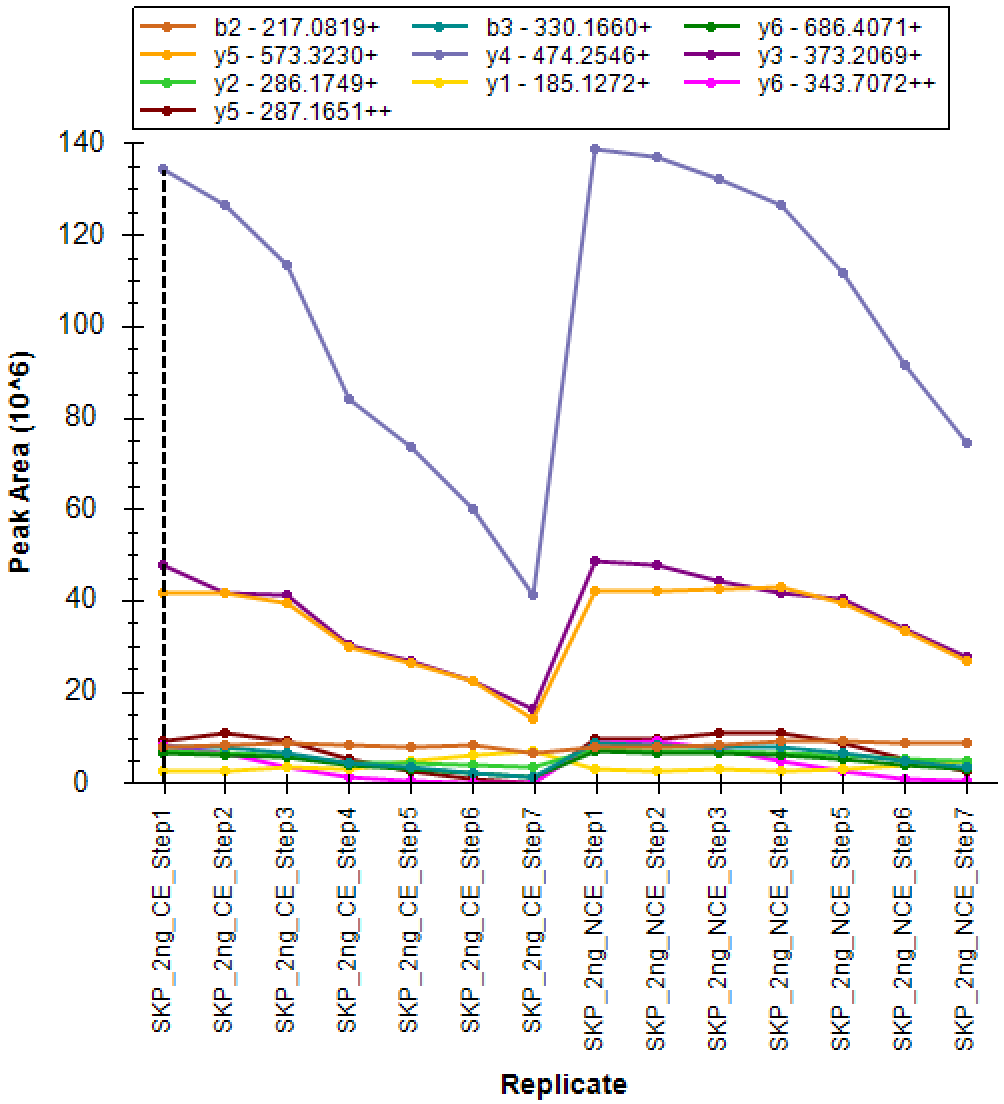

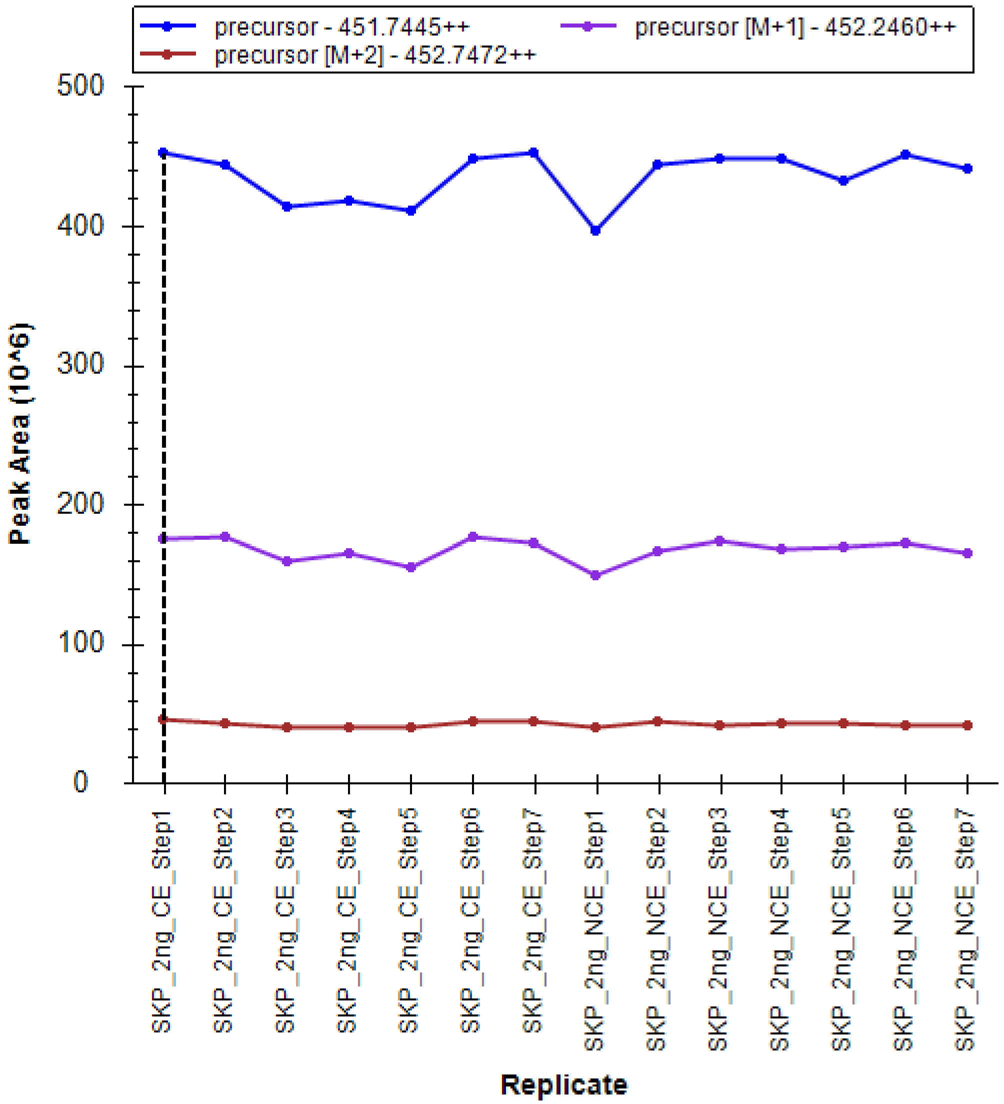

NDC80: QYDSSIQNLTR

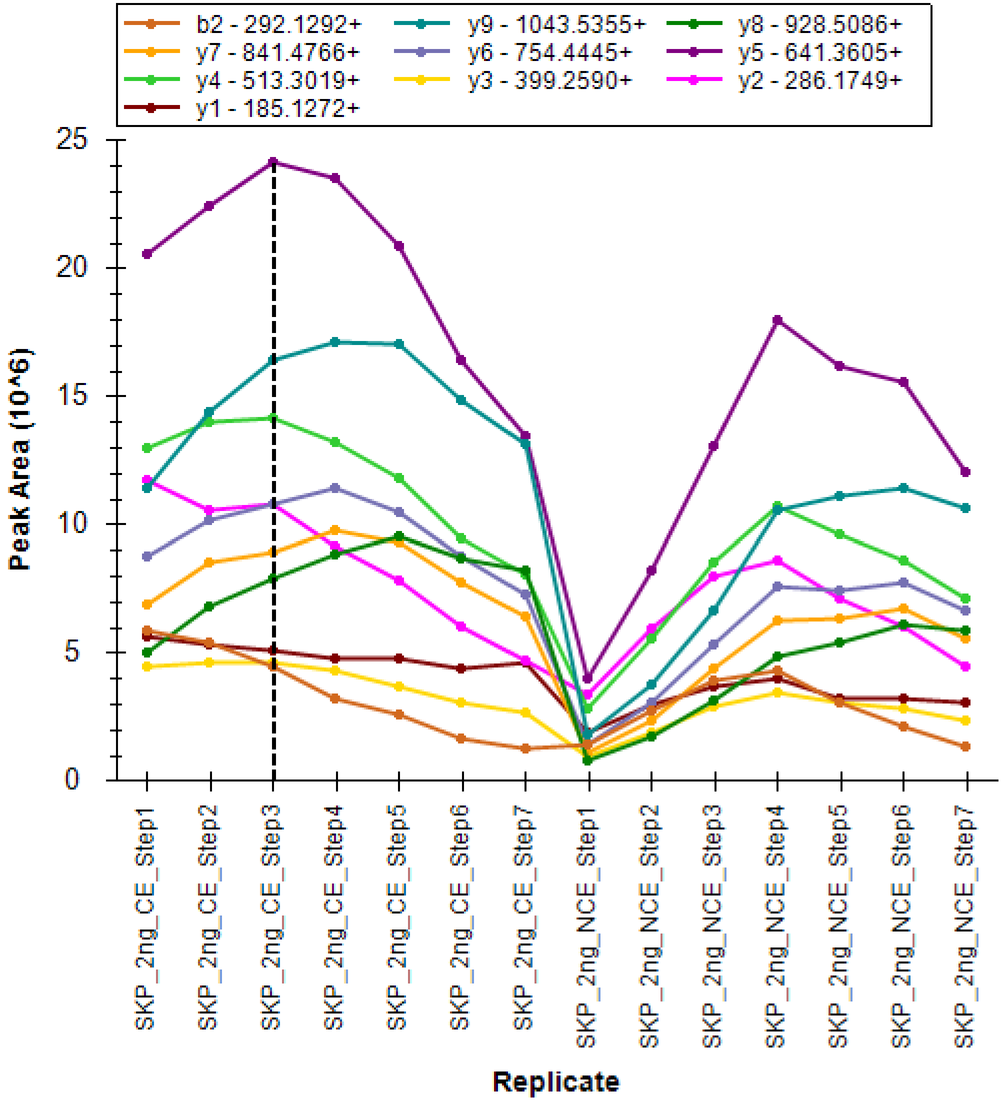

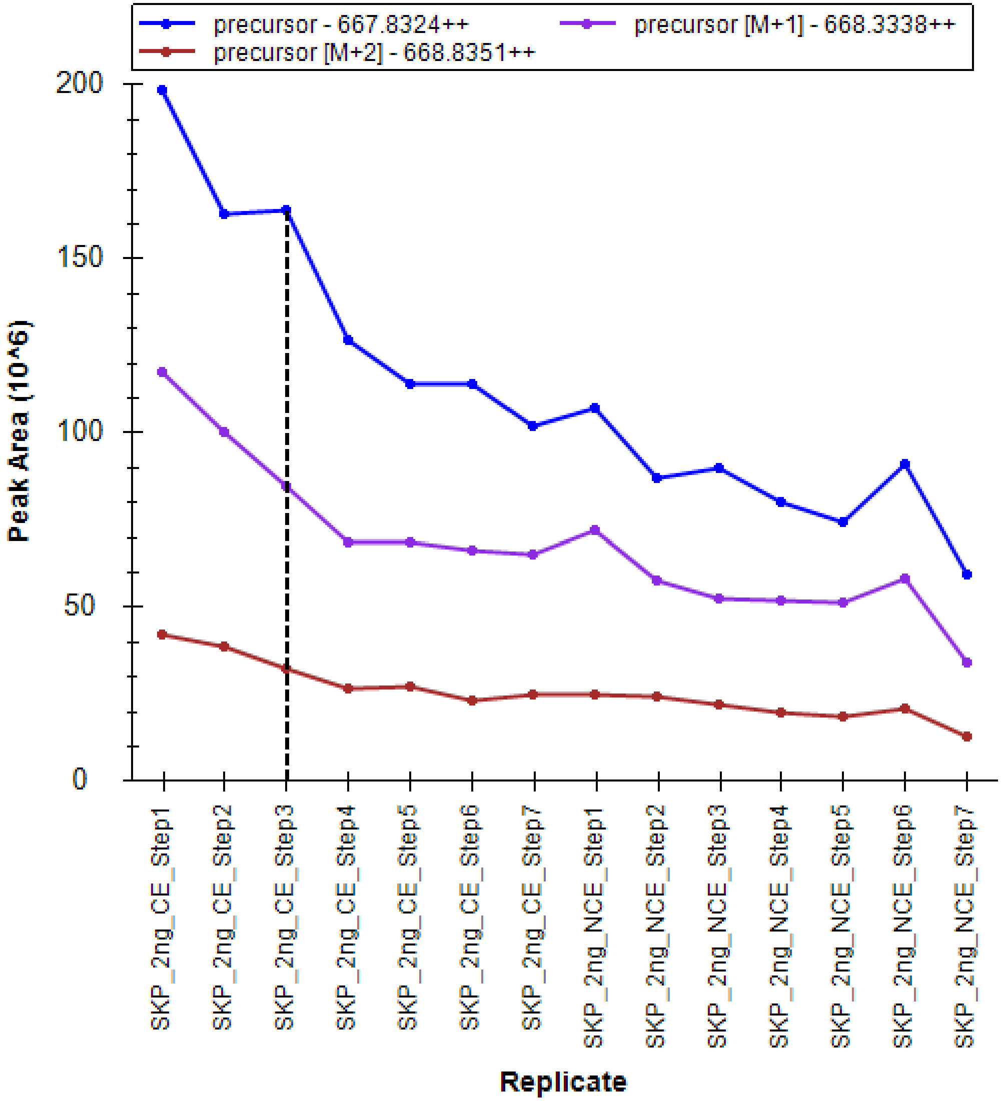

DSN1: ILDNTENYDDTELR

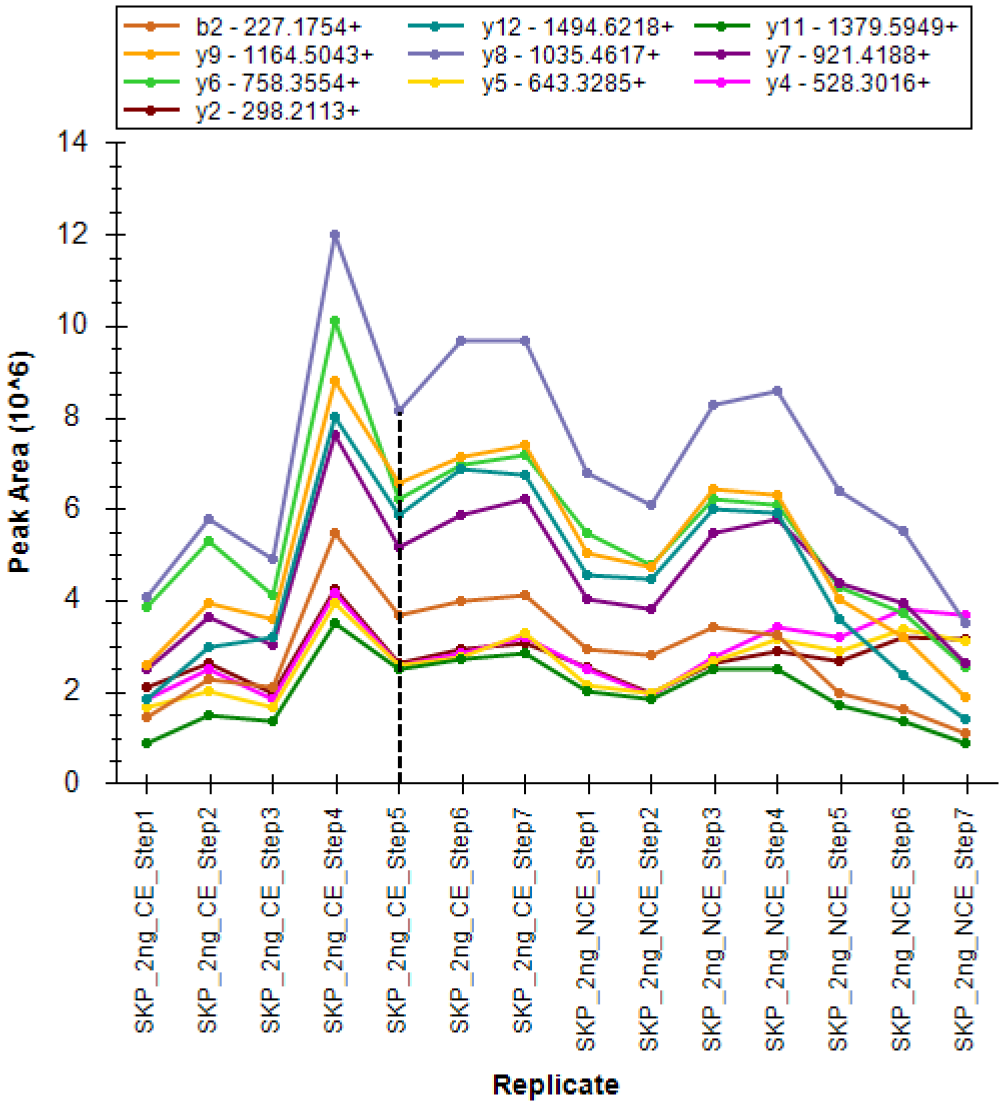

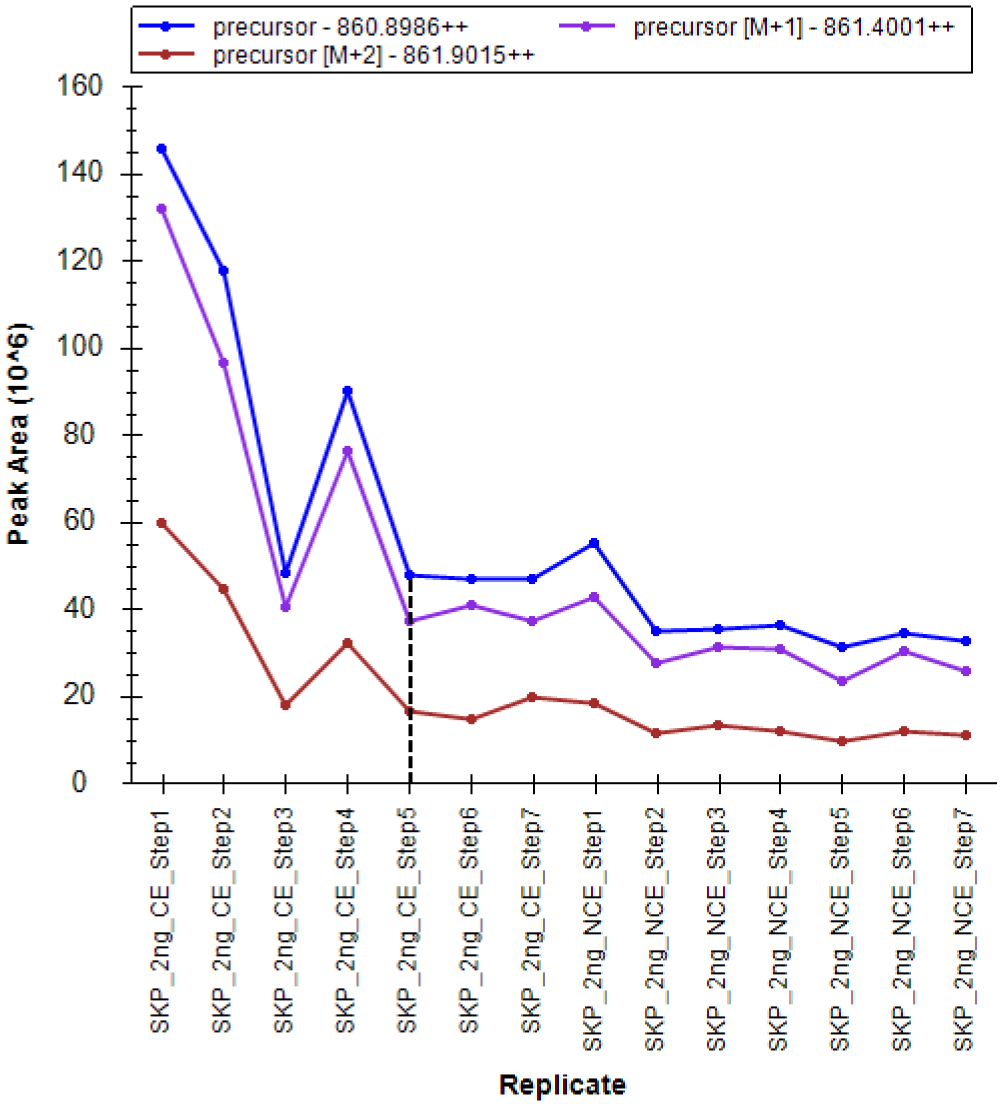

GST: GLVQPTR

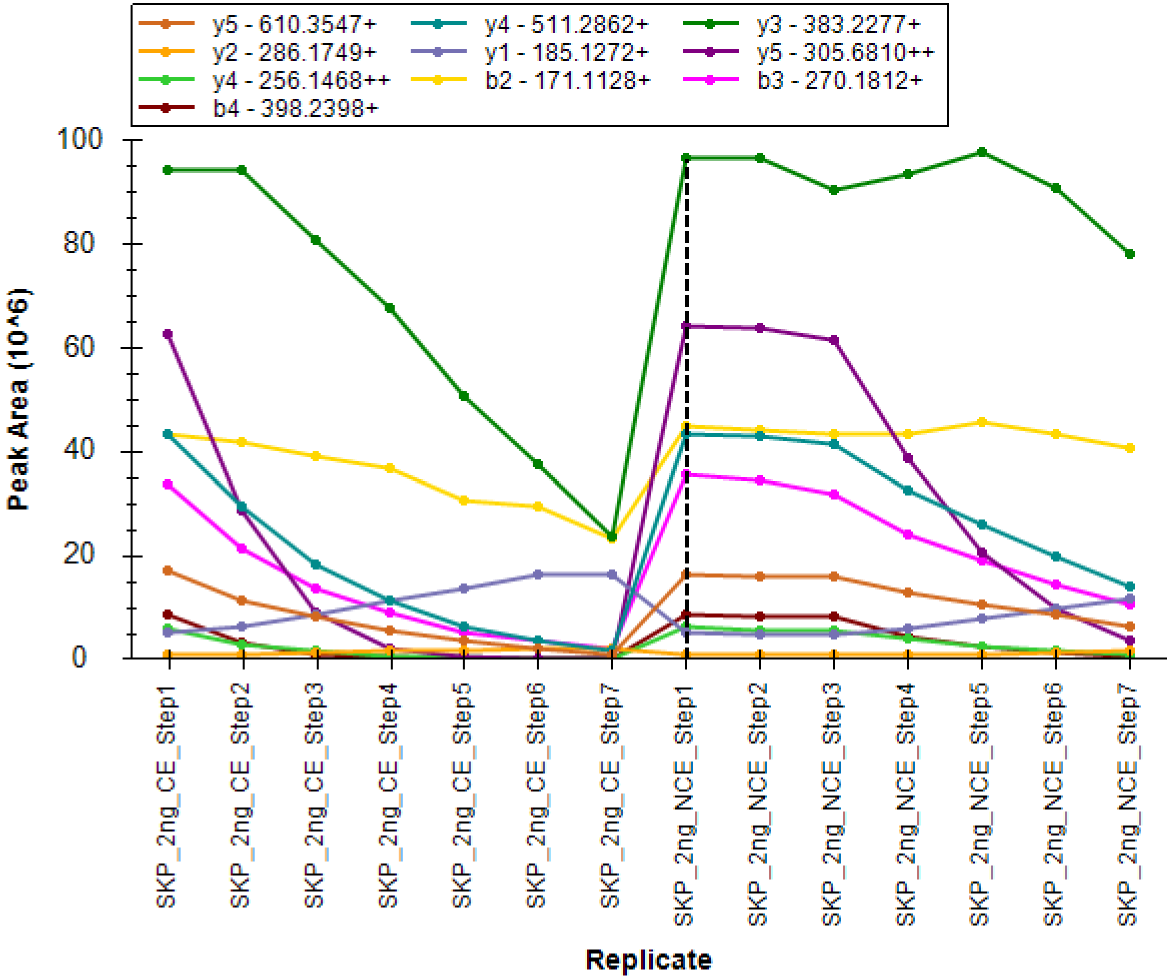
y5 - 610.3547+
y4 - 511.2862+
y3 - 383.2277+
y2 - 286.1749+
y1 - 185.1272+
y5 - 305.6810++
y4 - 256.1468++
b2 - 171.1128+
b3 - 270.1812+
b4 - 398.2398+
Peak Area (10^6)
100
80
60
40
20
0
SKP_2ng_CE_Step1
SKP_2ng_CE_Step2
SKP_2ng_CE_Step3
SKP_2ng_CE_Step4
SKP_2ng_CE_Step5
SKP_2ng_CE_Step6
SKP_2ng_CE_Step7
SKP_2ng_NCE_Step1
SKP_2ng_NCE_Step2
SKP_2ng_NCE_Step3
SKP_2ng_NCE_Step4
SKP_2ng_NCE_Step5
SKP_2ng_NCE_Step6
SKP_2ng_NCE_Step7
Replicate

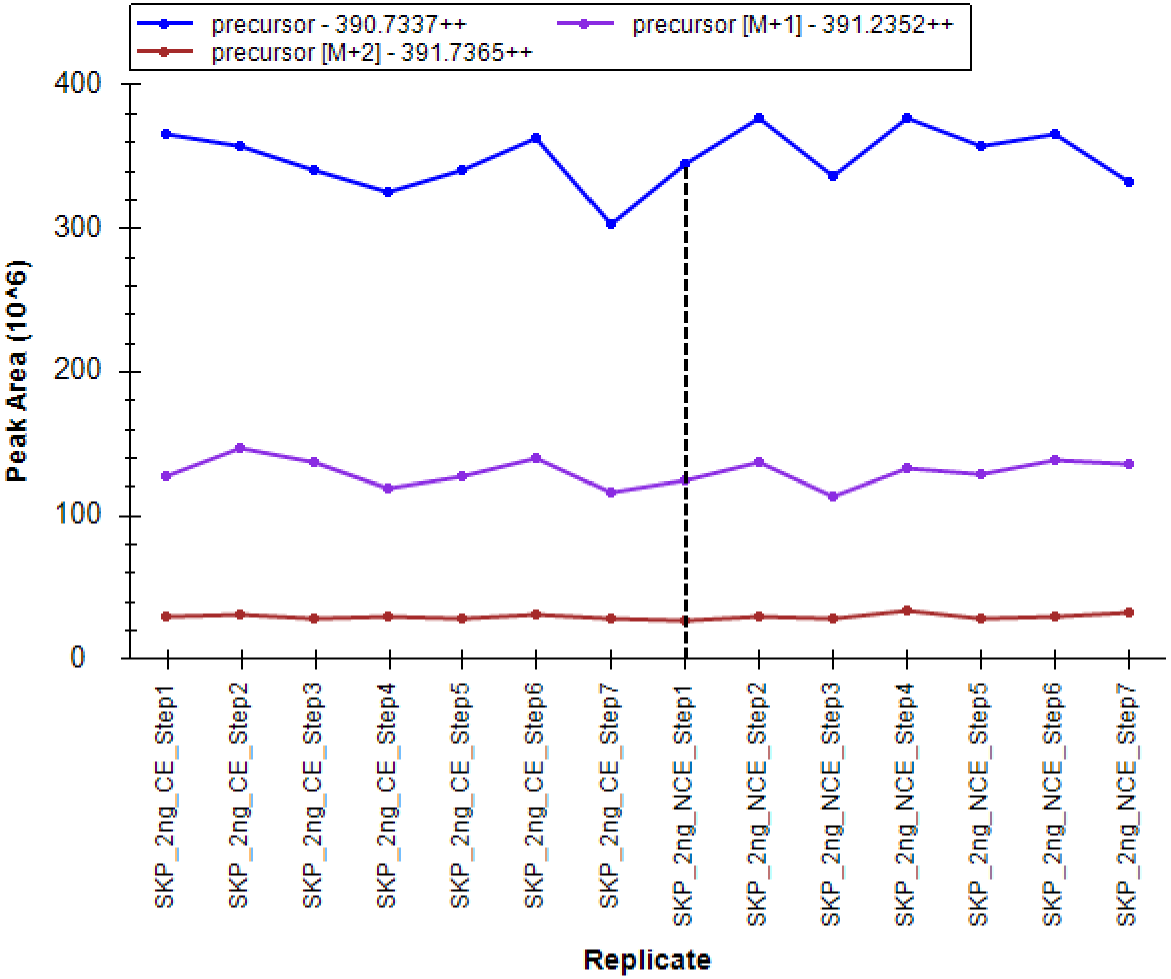
precursor - 390.7337++
precursor [M+1] - 391.2352++
precursor [M+2] - 391.7365++
Peak Area (10^6)
400
300
200
100
0
SKP_2ng_CE_Step1
SKP_2ng_CE_Step2
SKP_2ng_CE_Step3
SKP_2ng_CE_Step4
SKP_2ng_CE_Step5
SKP_2ng_CE_Step6
SKP_2ng_CE_Step7
SKP_2ng_NCE_Step1
SKP_2ng_NCE_Step2
SKP_2ng_NCE_Step3
SKP_2ng_NCE_Step4
SKP_2ng_NCE_Step5
SKP_2ng_NCE_Step6
SKP_2ng_NCE_Step7
Replicate

CBF1: LSTEDEEIHSAR

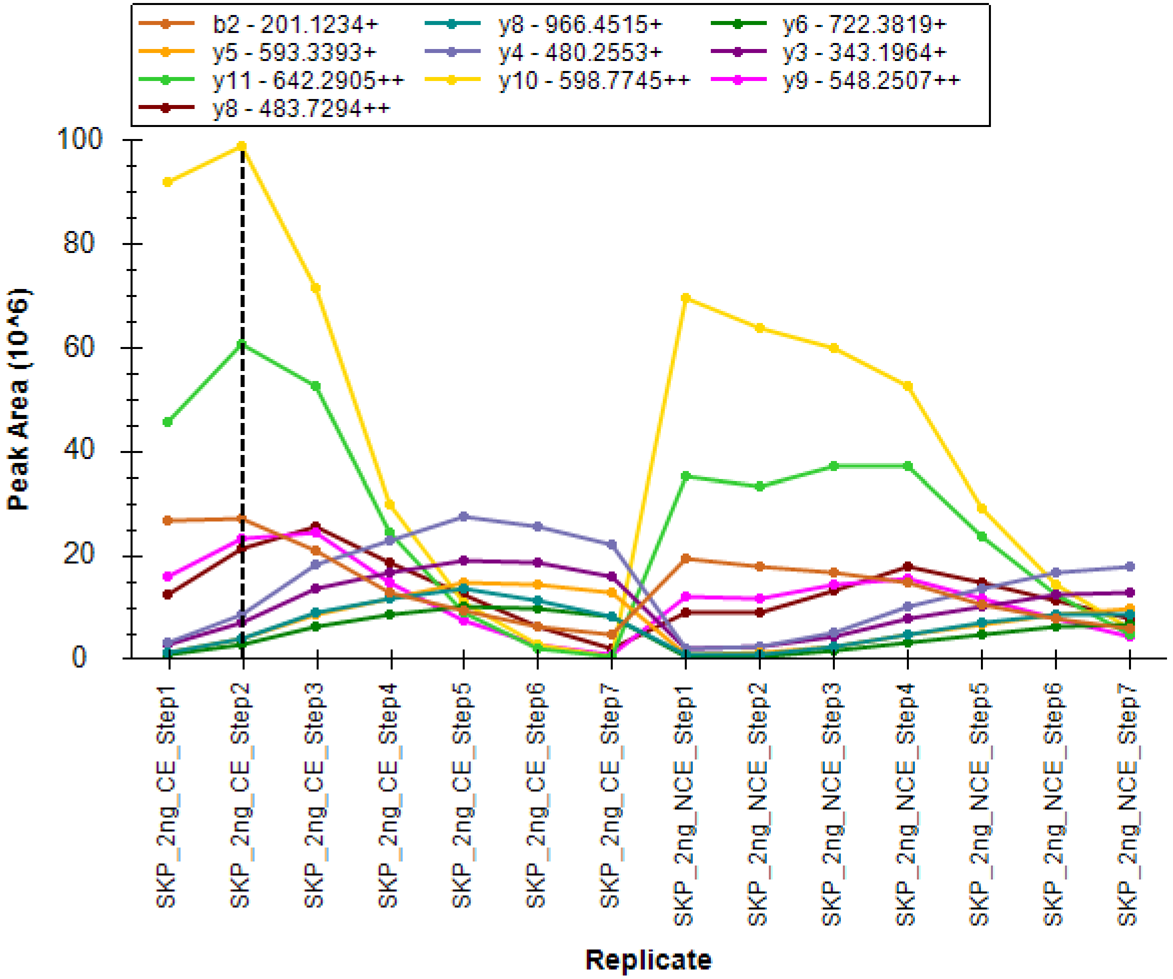

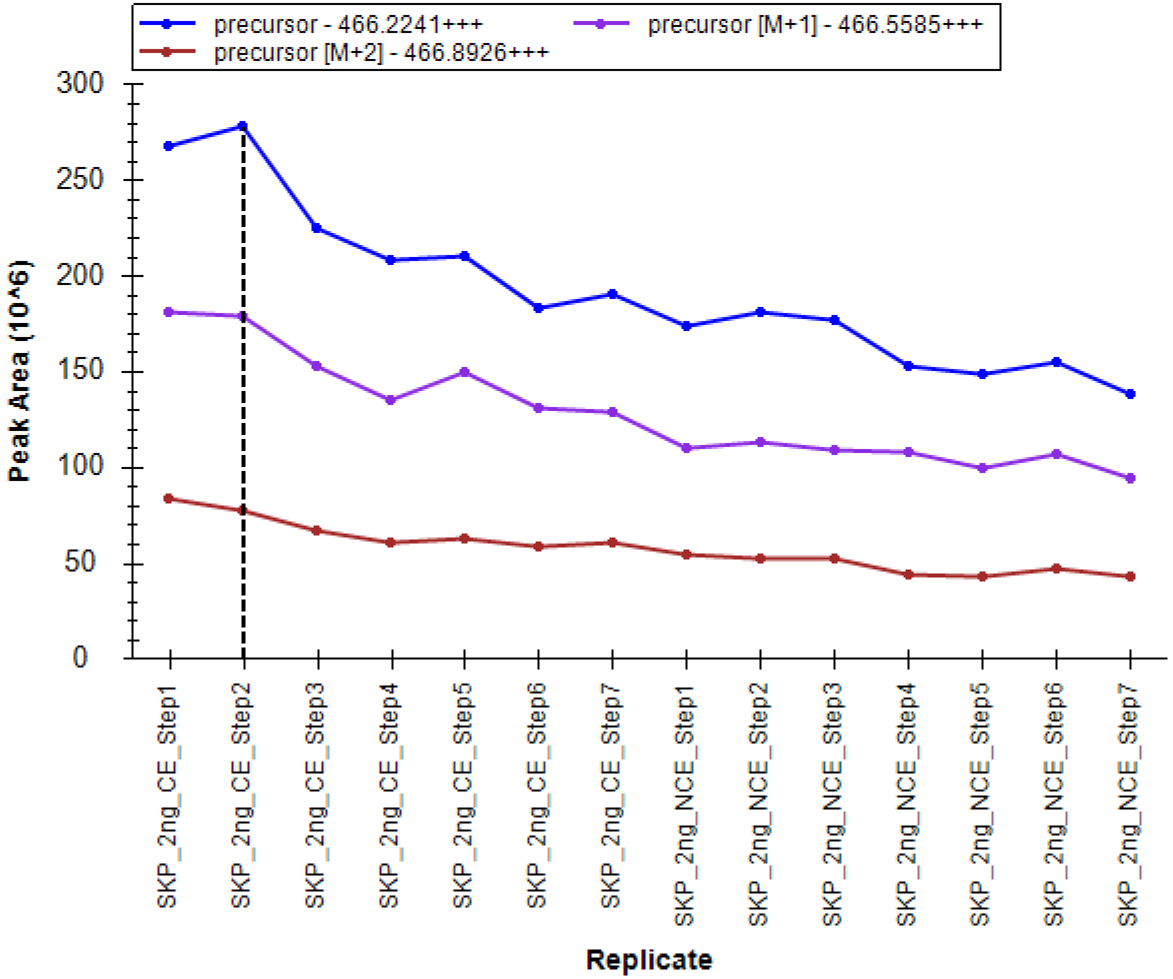

CEP3: LVYLTER

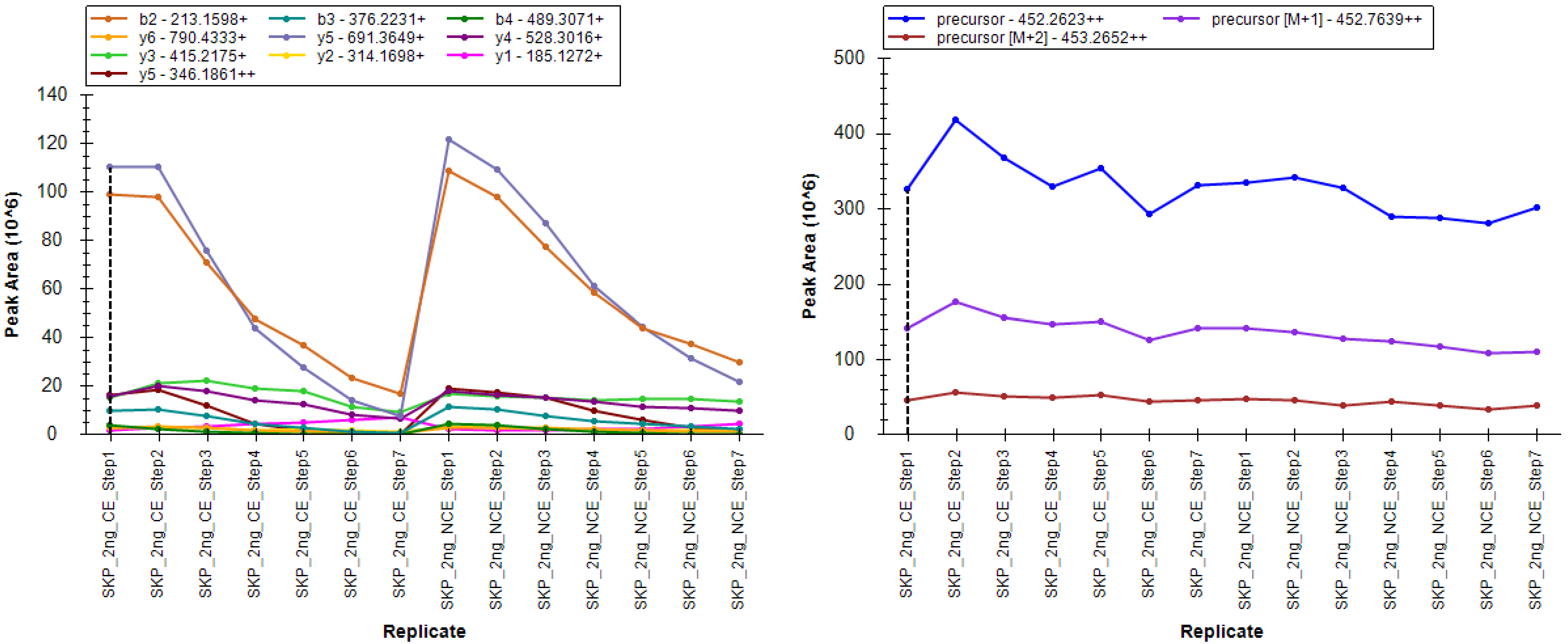

CTF13: TGLADFTR

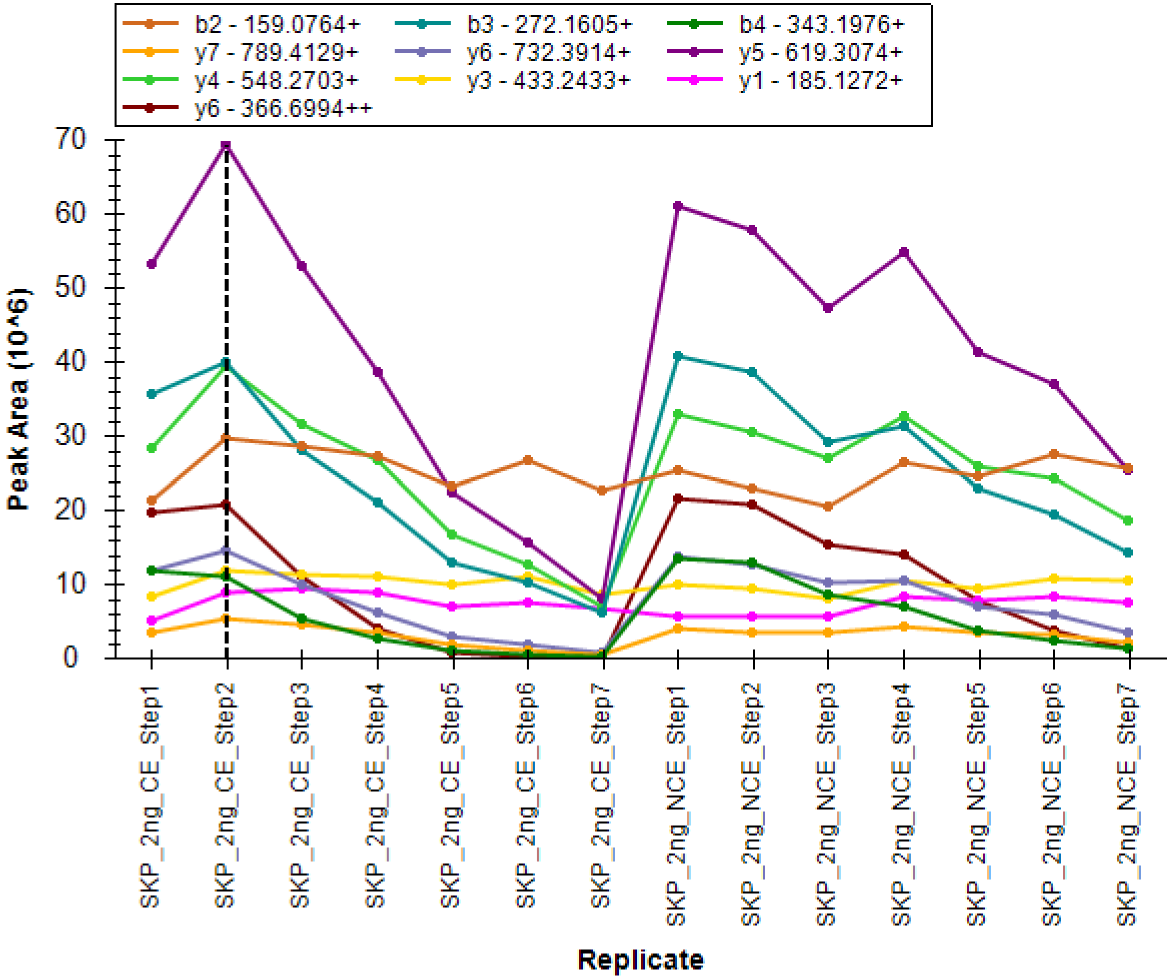

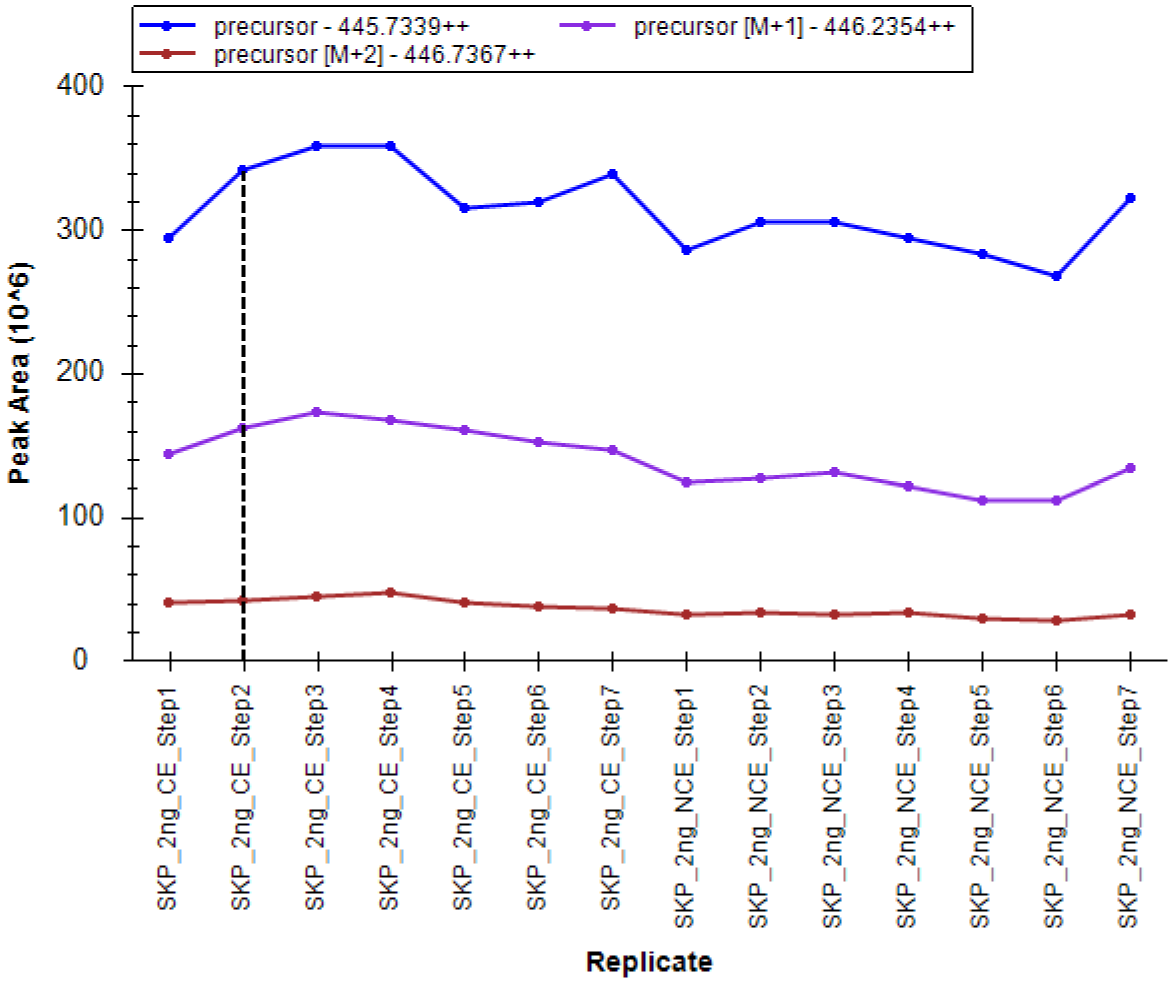

HTA2: AGLTFPVGR

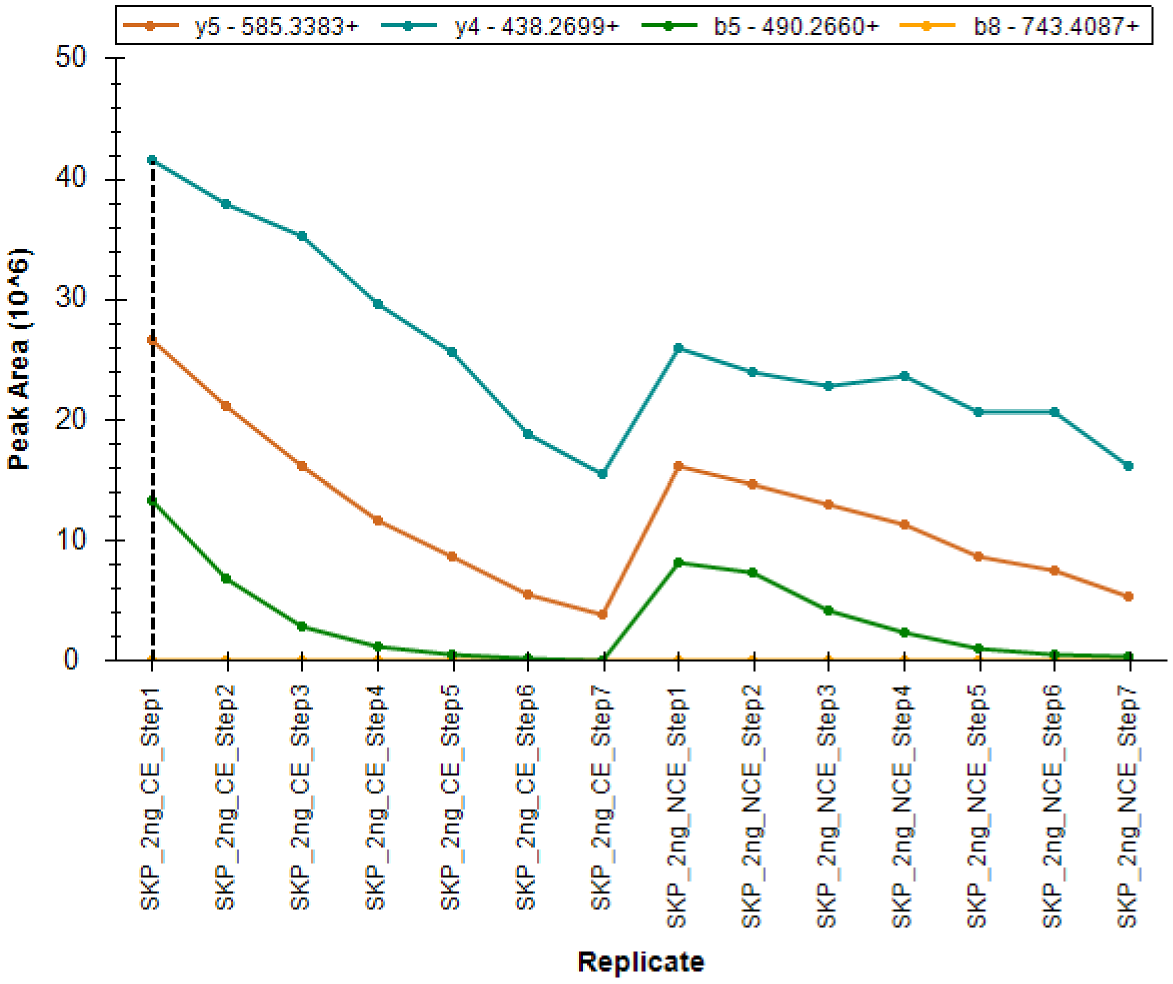

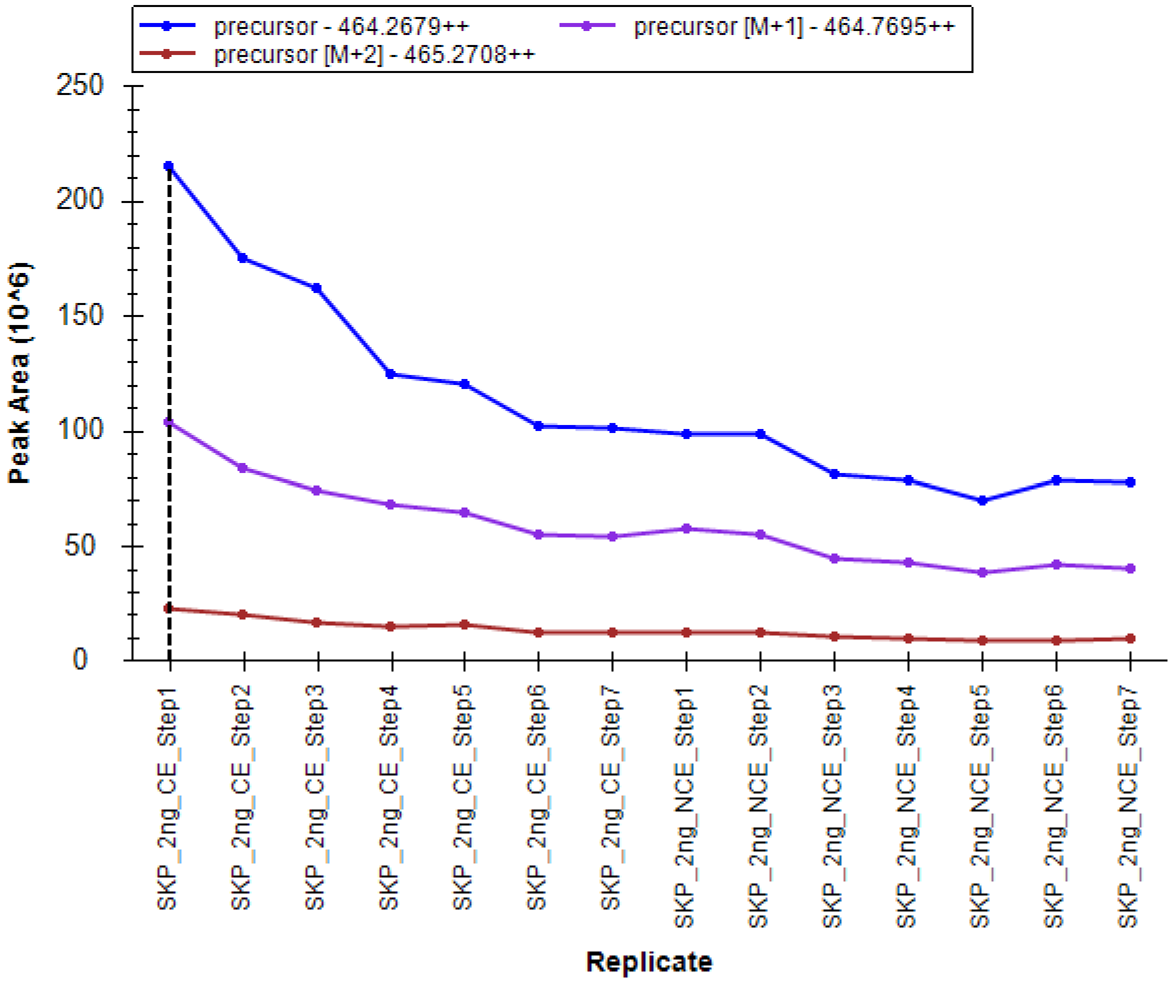

HHF1: ISGLIYEEVR

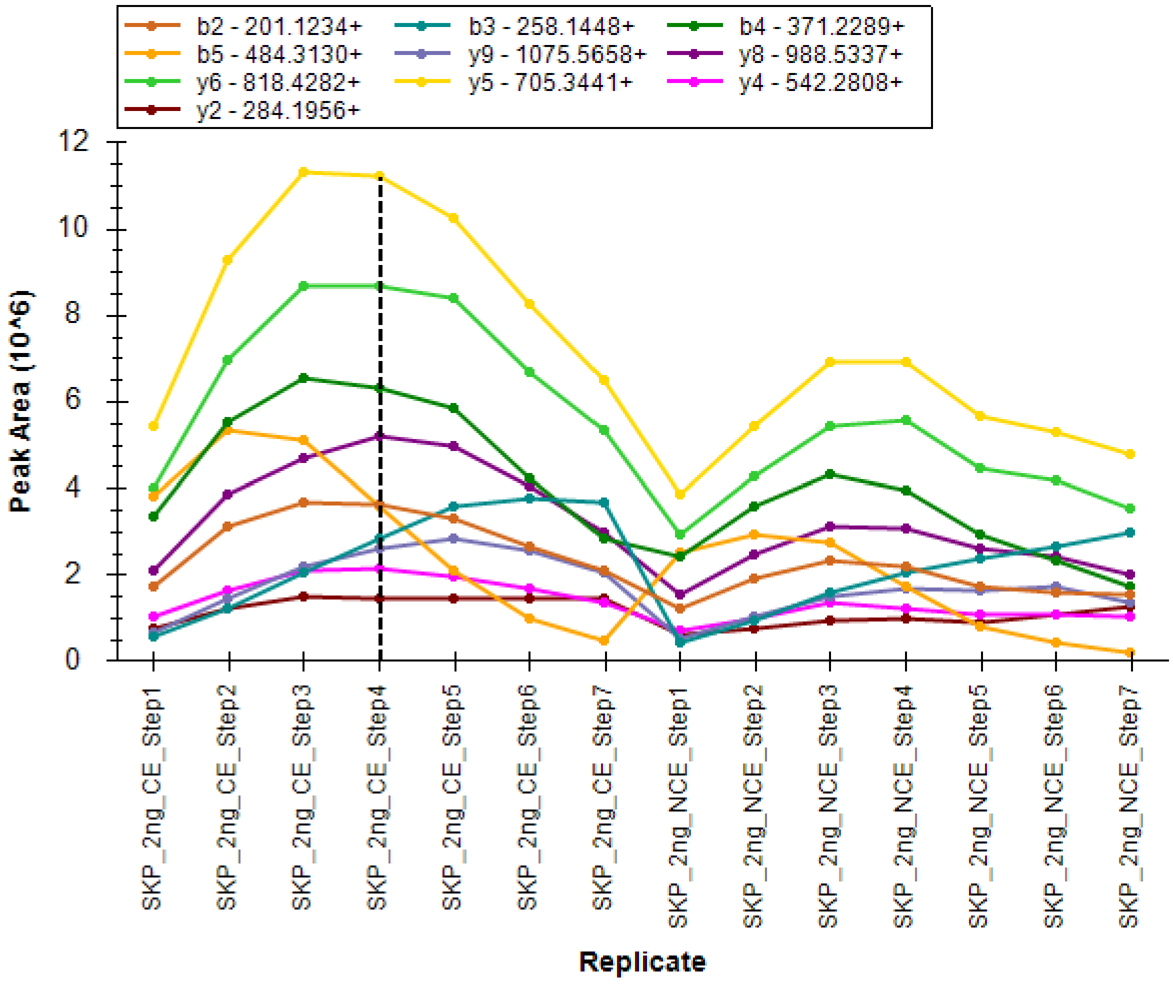

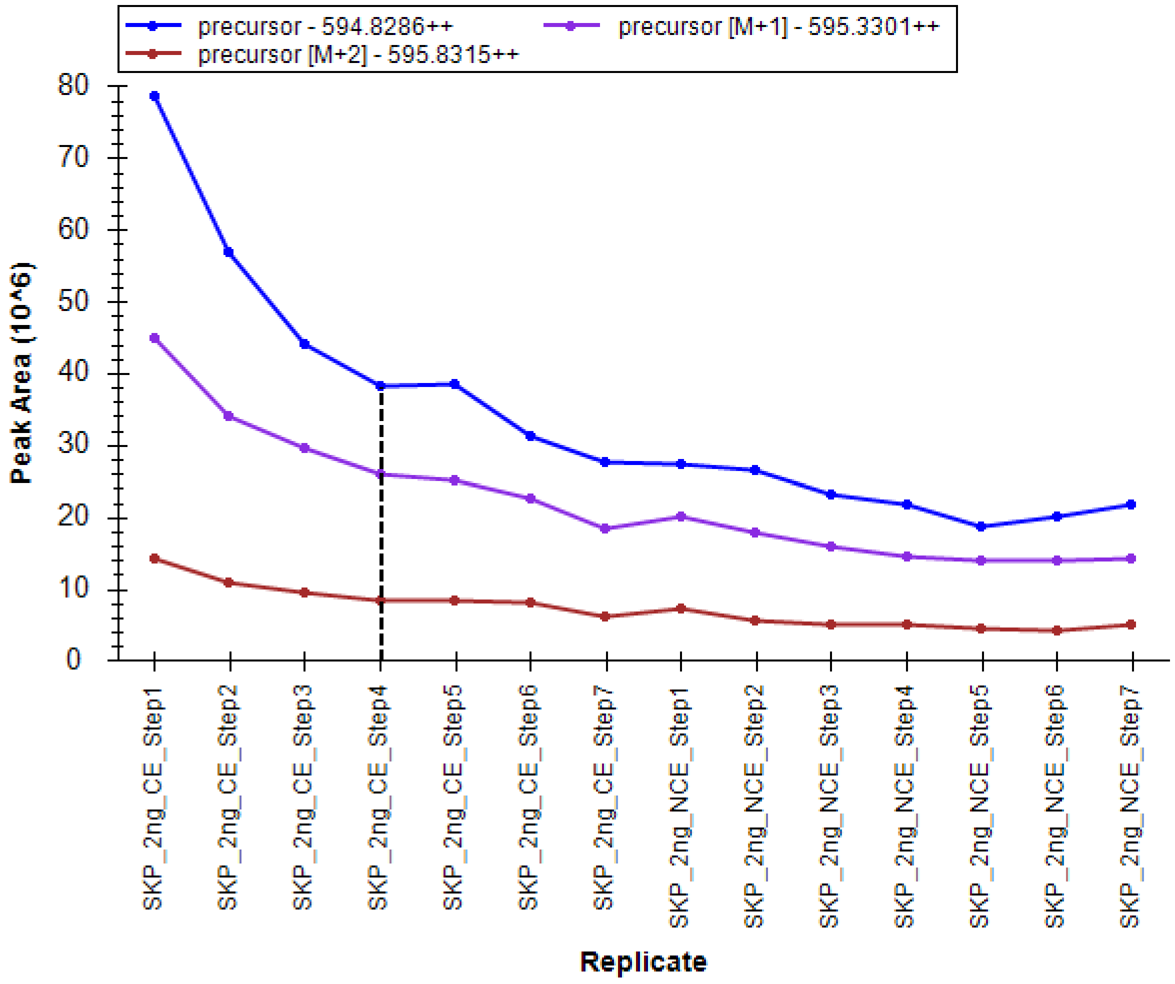

HHT1: STELLIR

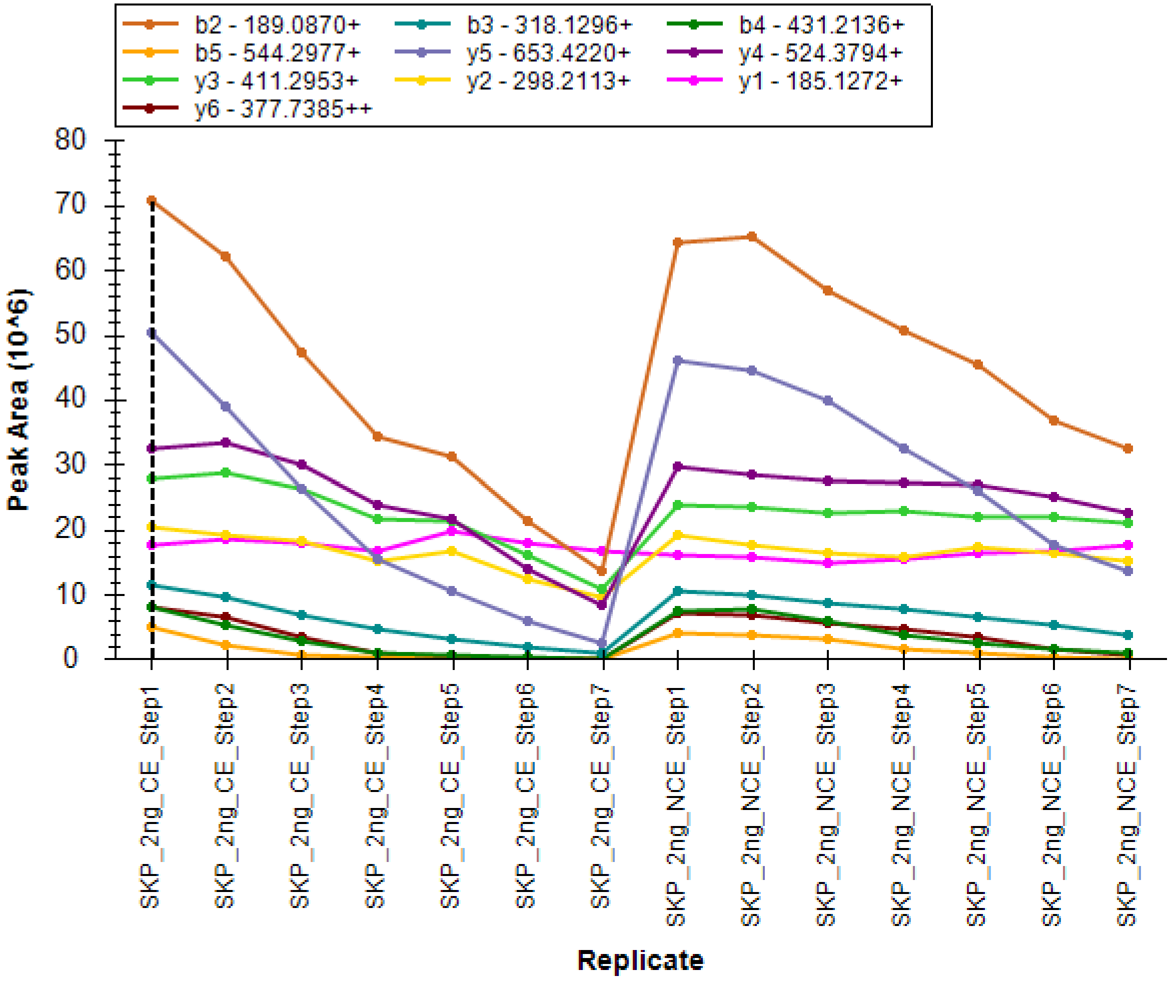

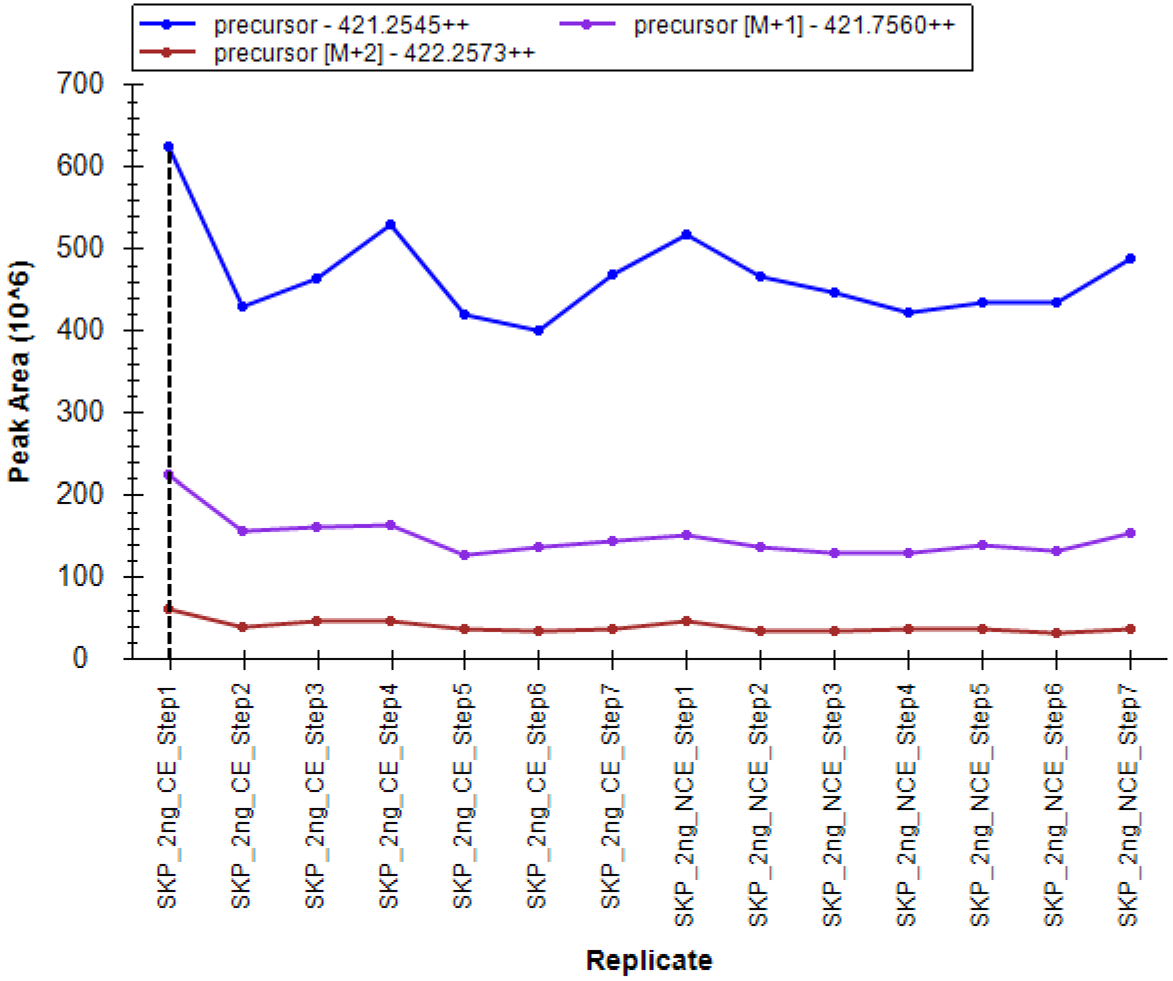

MIF2-1: YSLDTSESPSVR

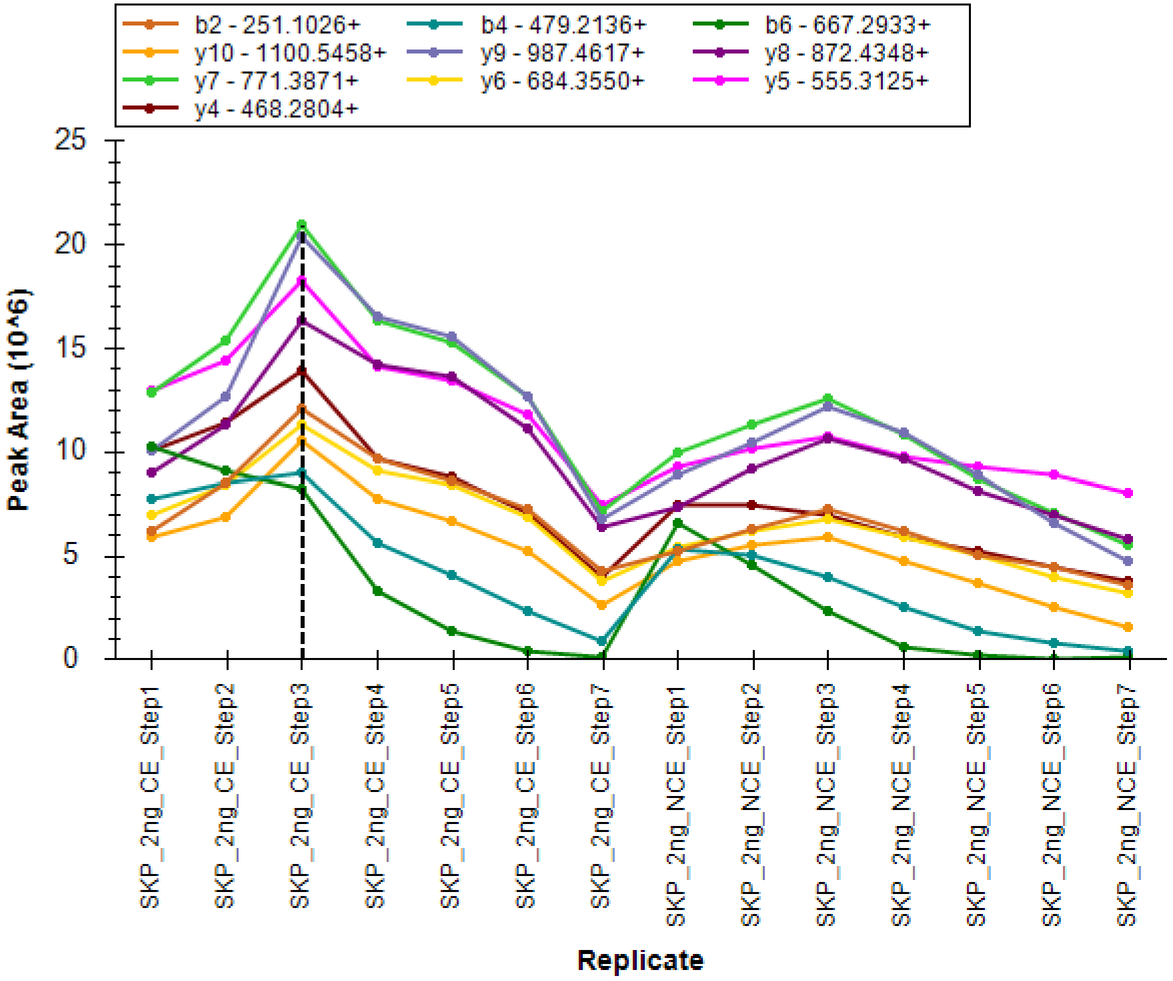

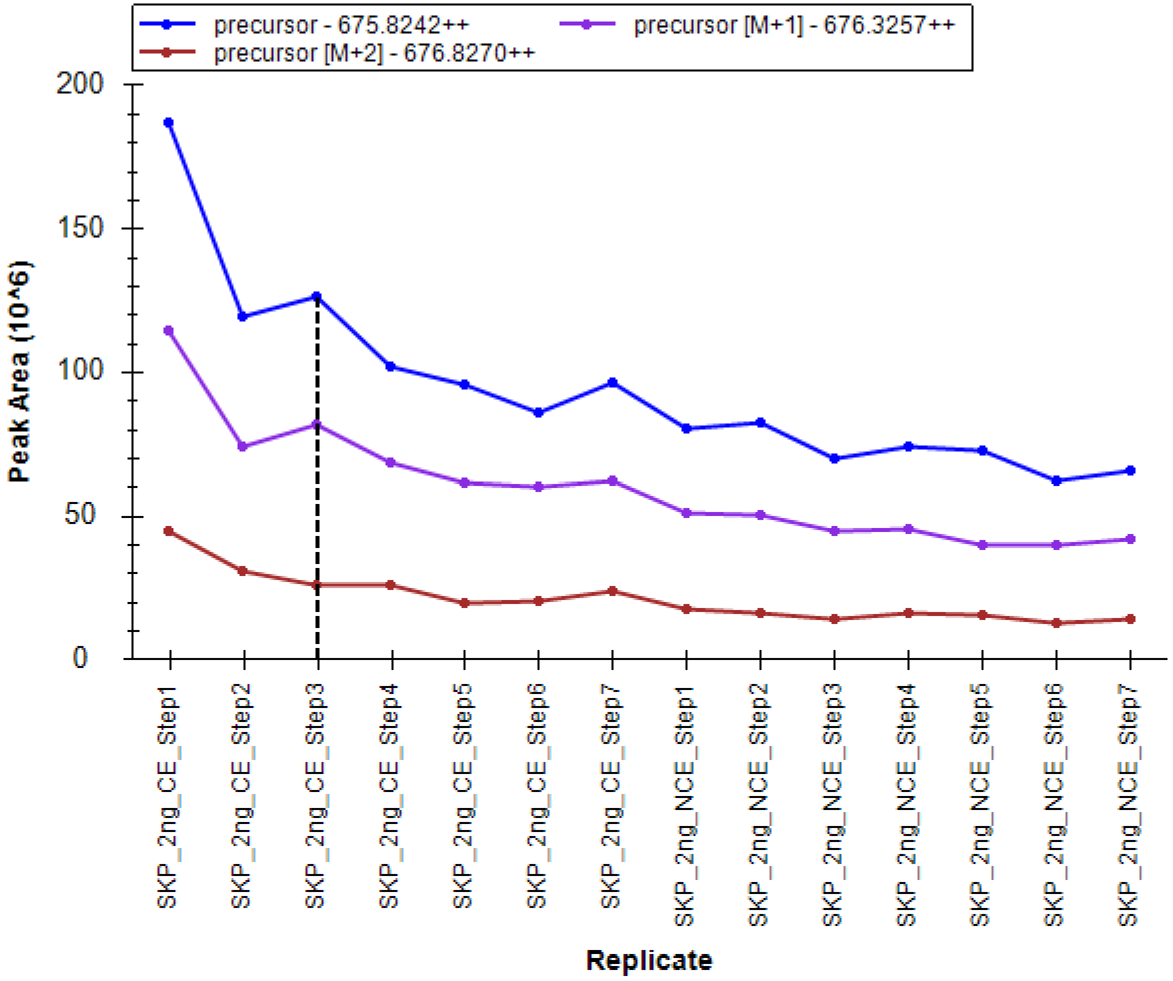

CTF19: QQLSLLDDDQVR

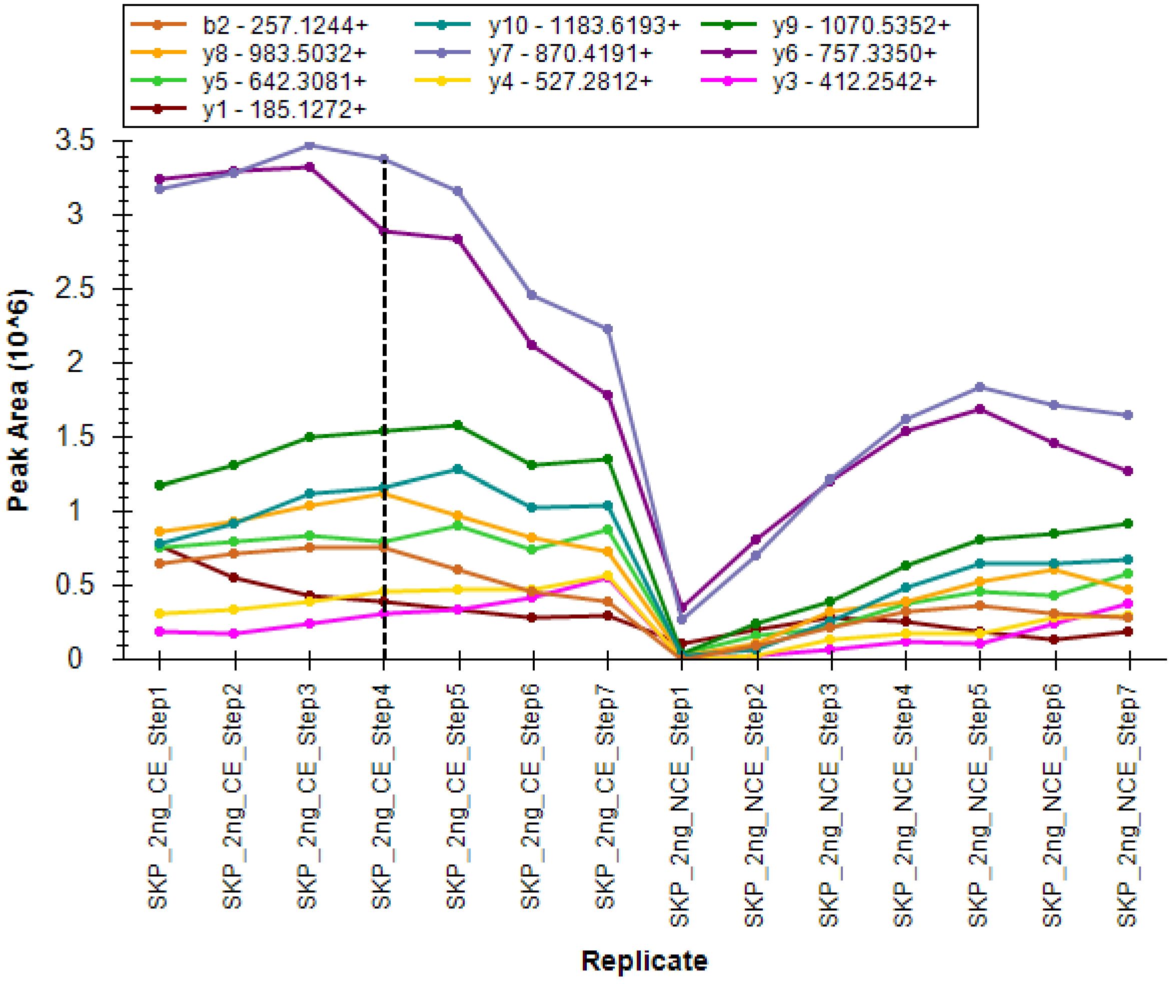

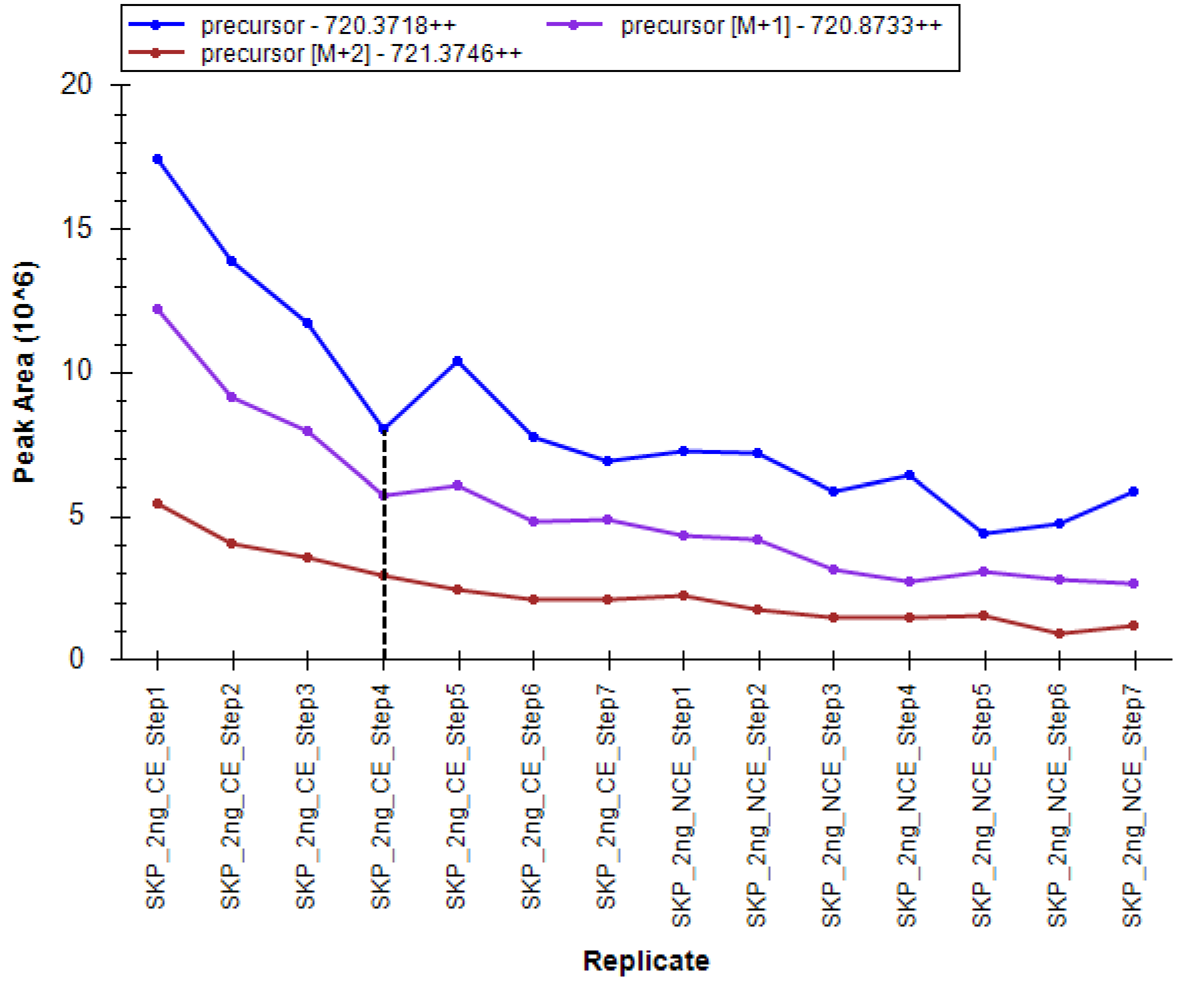

CTF3: DAPGSATLILQR

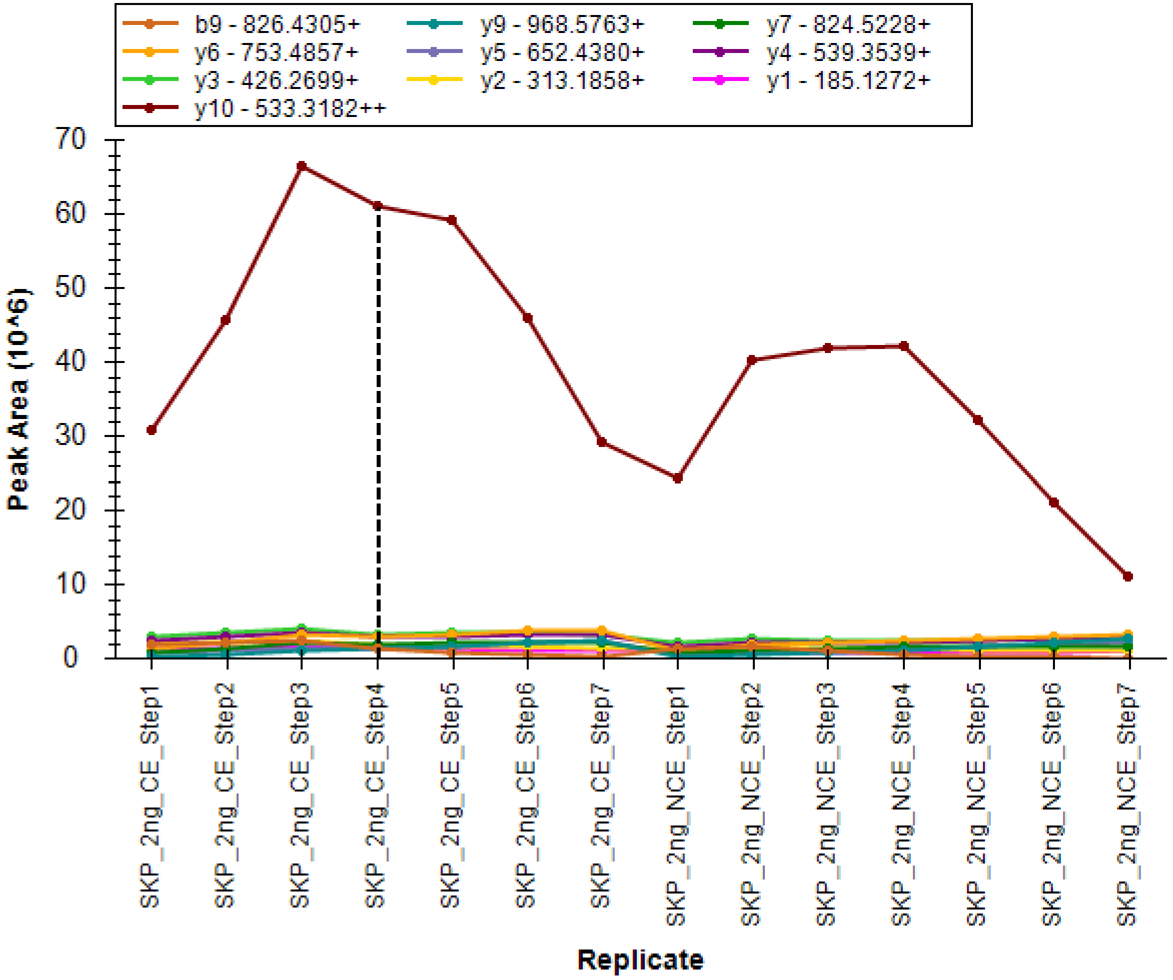

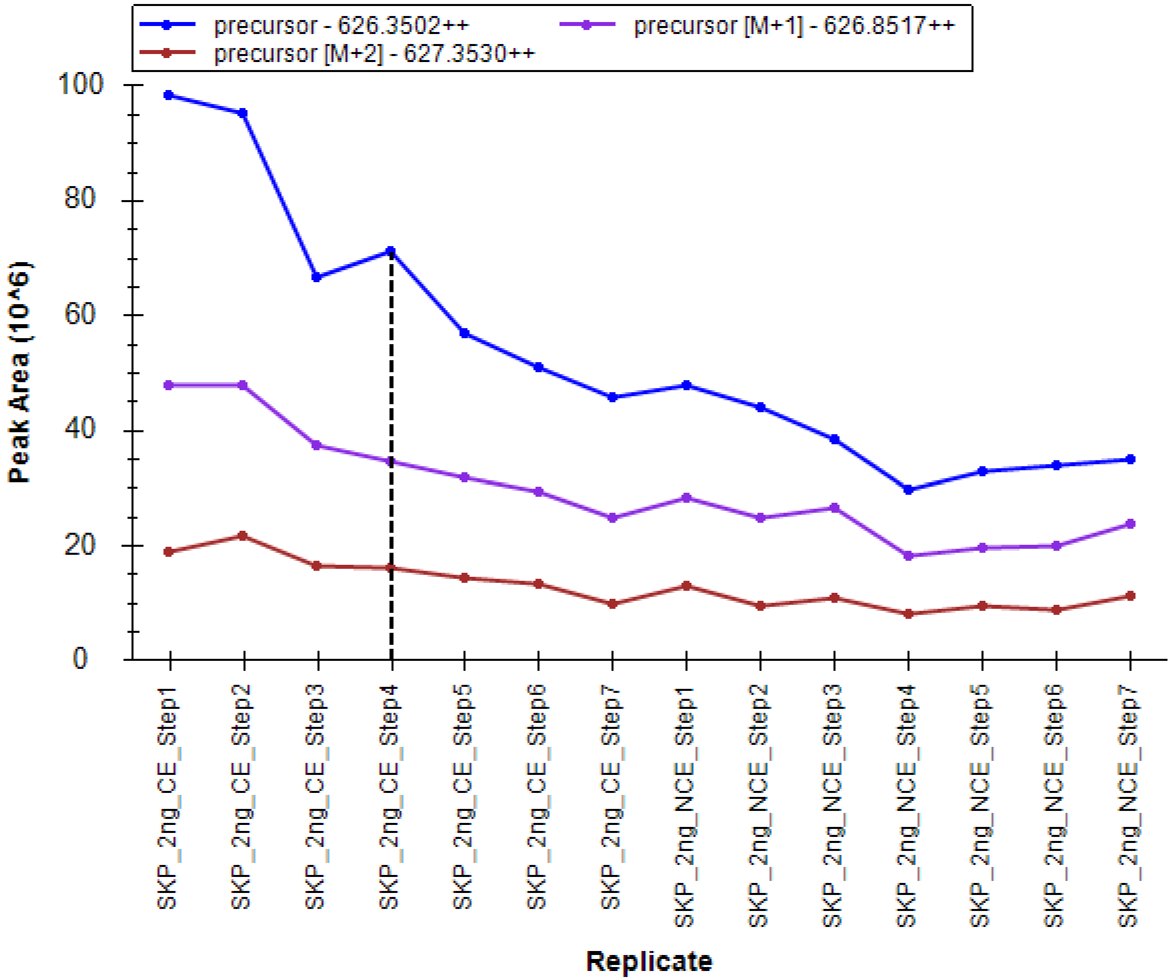

OKP1: VIQAEYR

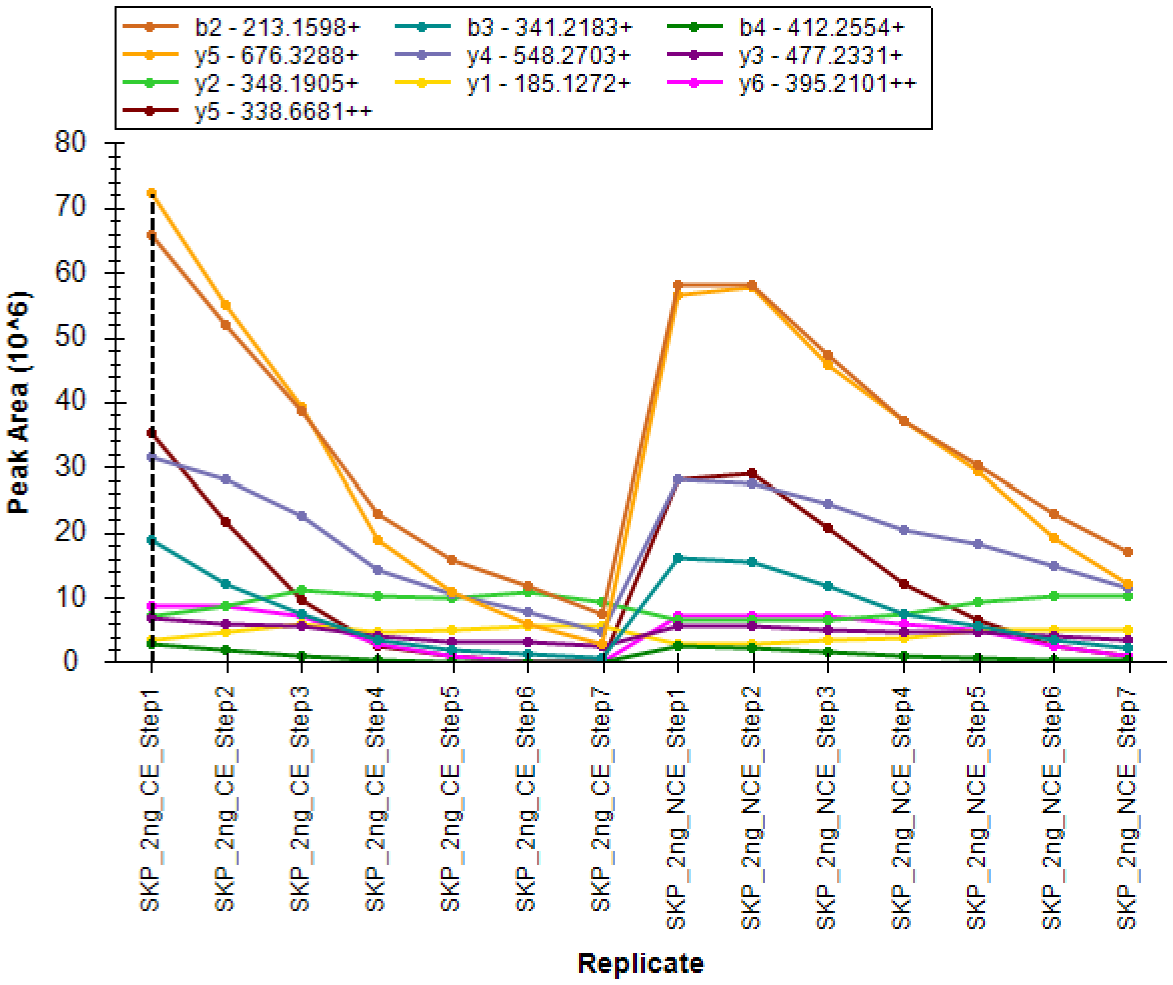

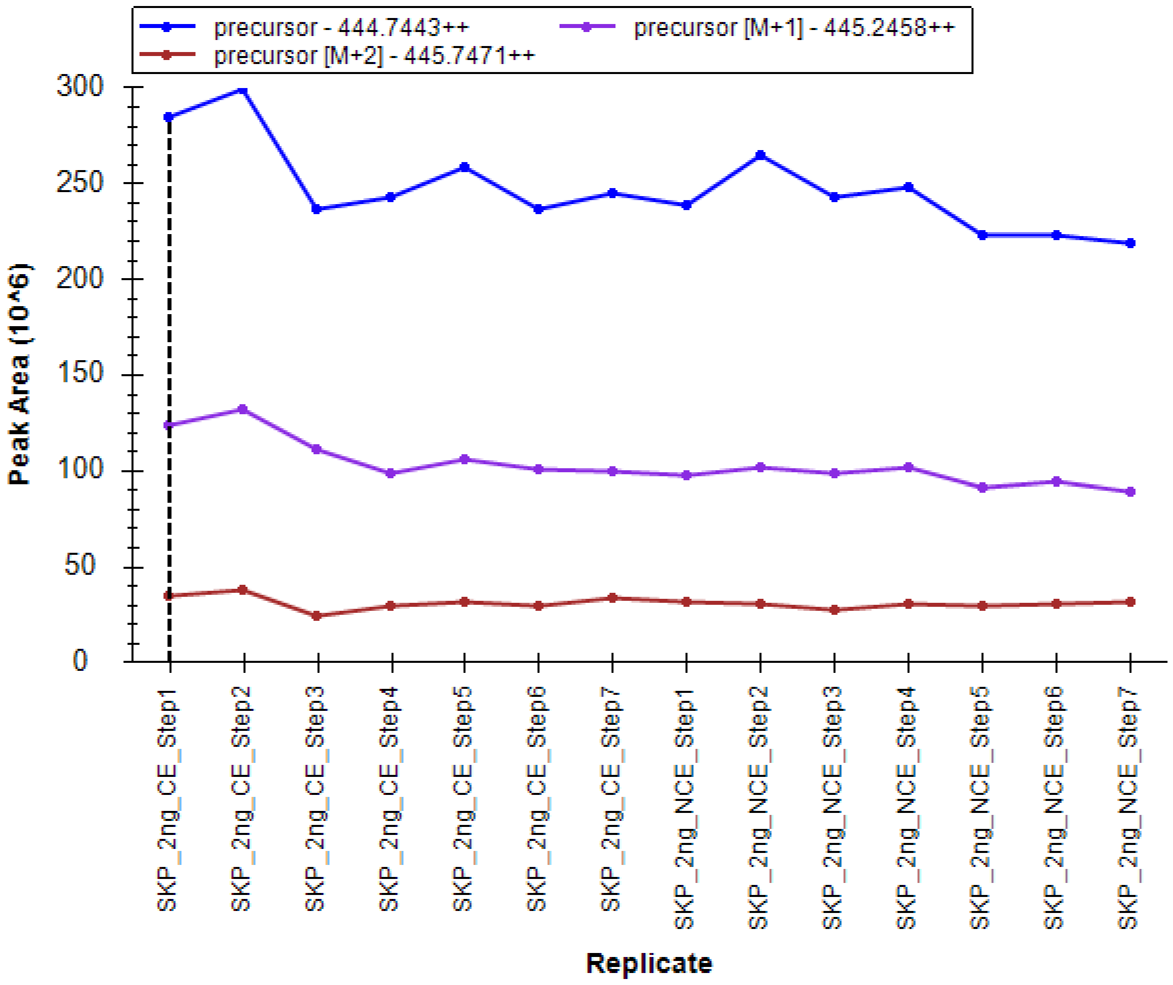

Supplement: Supplementary file 30 [file LSA-2024-03007_SdataFS10.pdf]
