# Supplementary material for: Concatemer-assisted stoichiometry analysis: targeted mass spectrometry for protein quantification
Source: Life Sci Alliance. 2024 Dec 31;8(3):e202403007. doi: 10.26508/lsa.202403007 (PMC11707388; doi:10.26508/lsa.202403007)

CKP Calibration Curves

GST-1: YGVSR

Quantifier ion: y4+, 291.1557++ → 418.2409+

AMR: 78.125 – 60000 pM

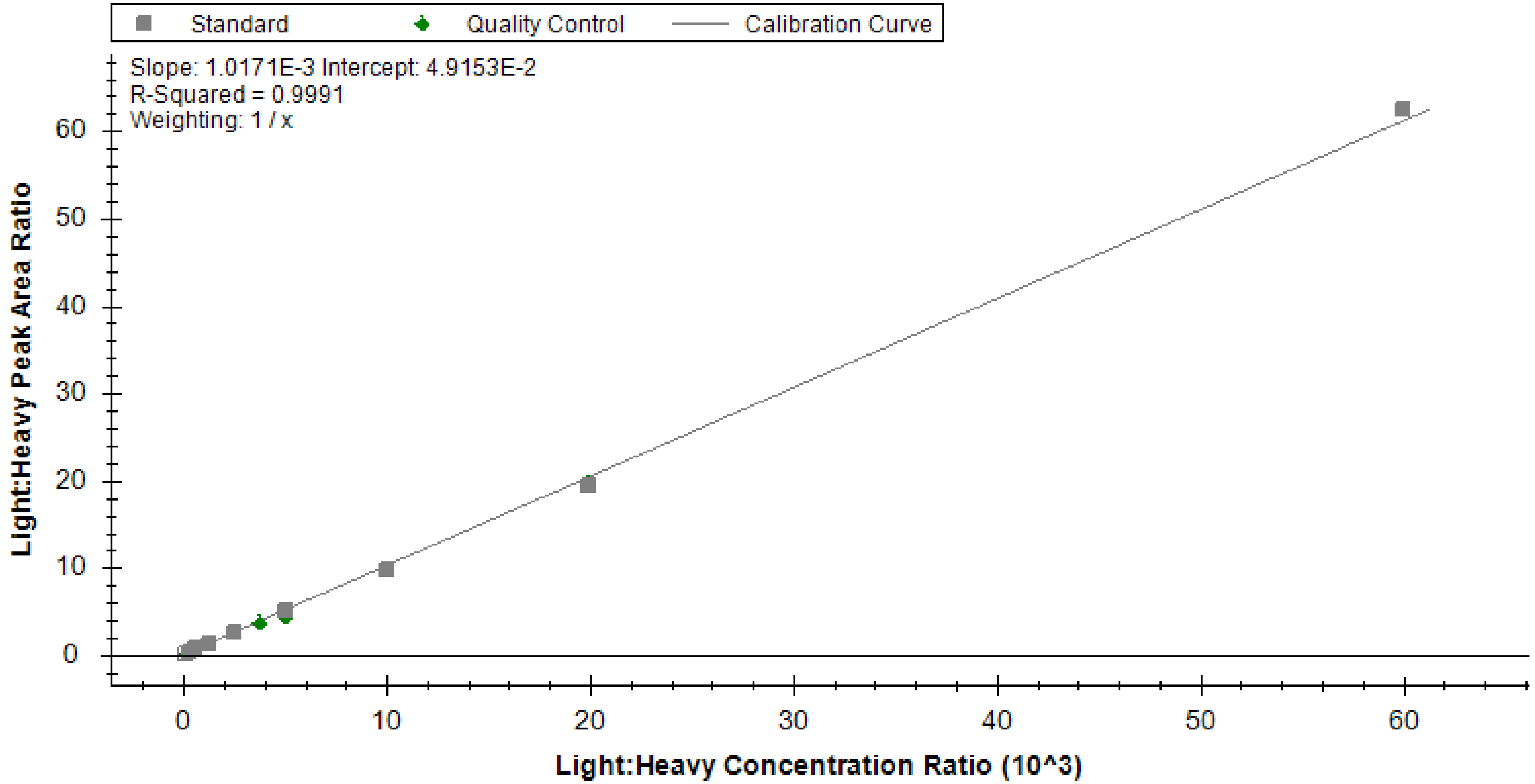

GST-2: GLVQPTR

Quantifier ion: y3+, 385.7296++ → 373.2194+

AMR: 78.125 – 60000 pM

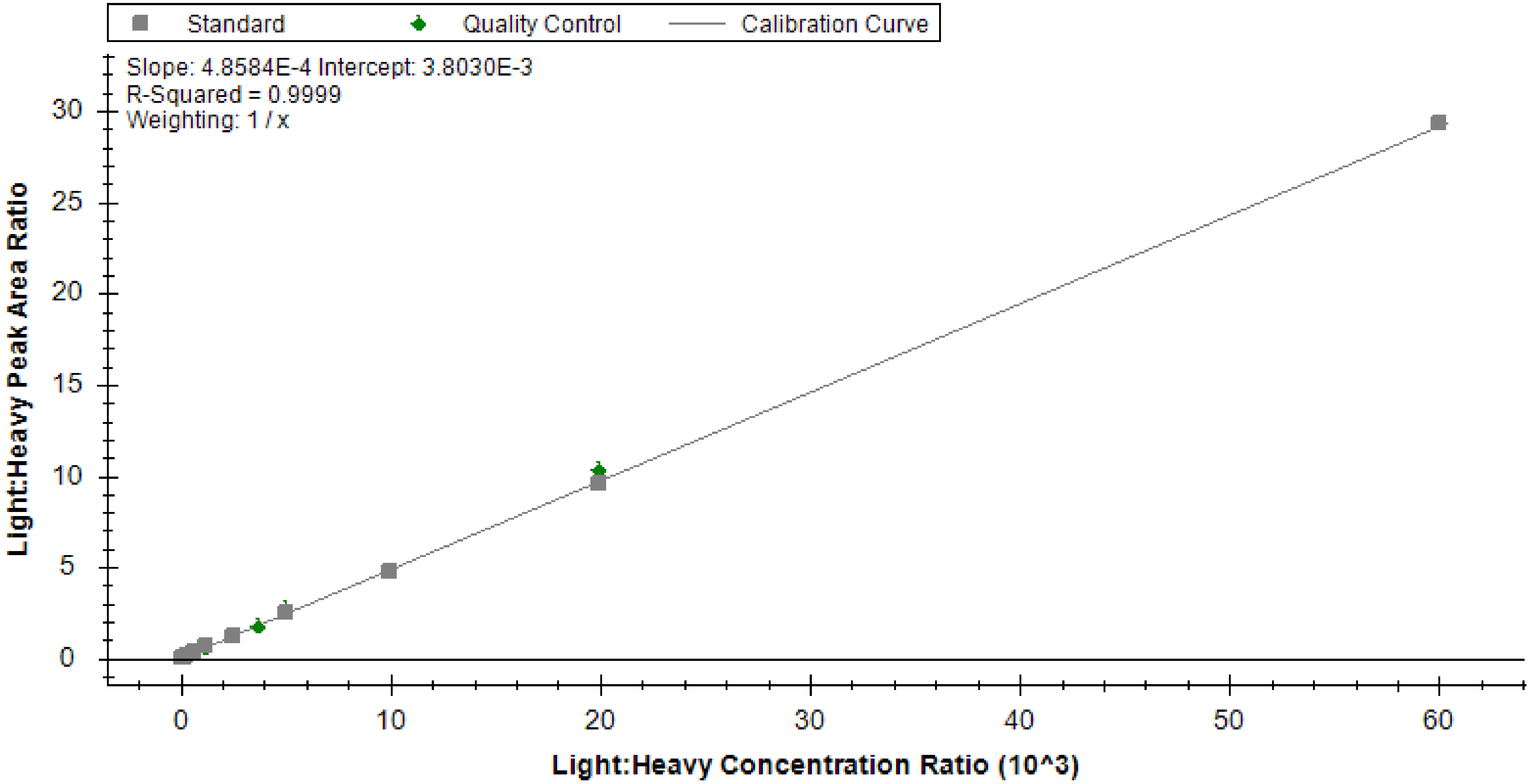

CBF1: LSTEDEEIHSAR

Quantifier ion: y10++, 462.8880+++ → 593.7704++

AMR: 78.125 – 60000 pM

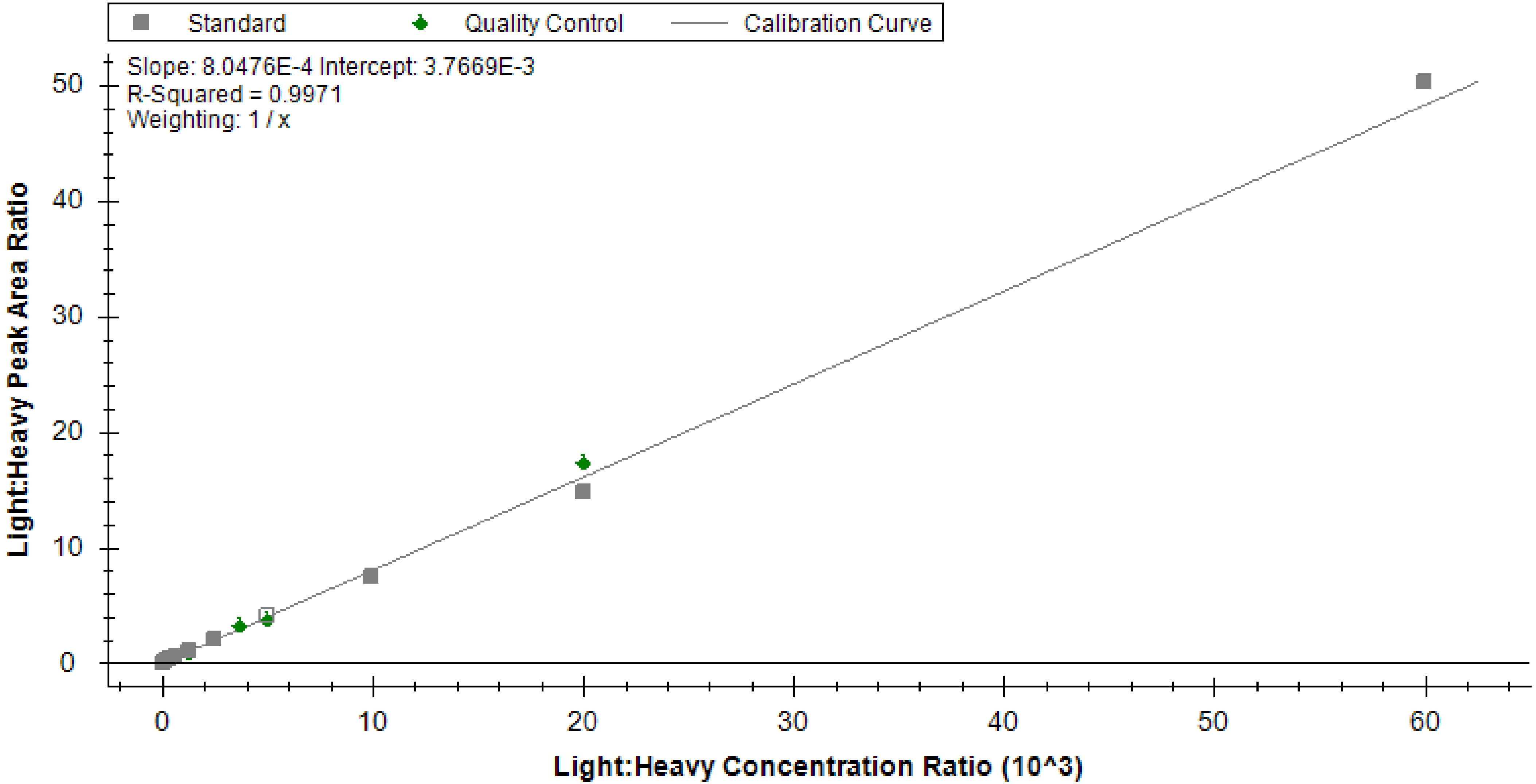

CTF13: TGLADFTR

Quantifier ion: y4+, 440.7298++ → 538.2620+

AMR: 156.25 – 20000 pM

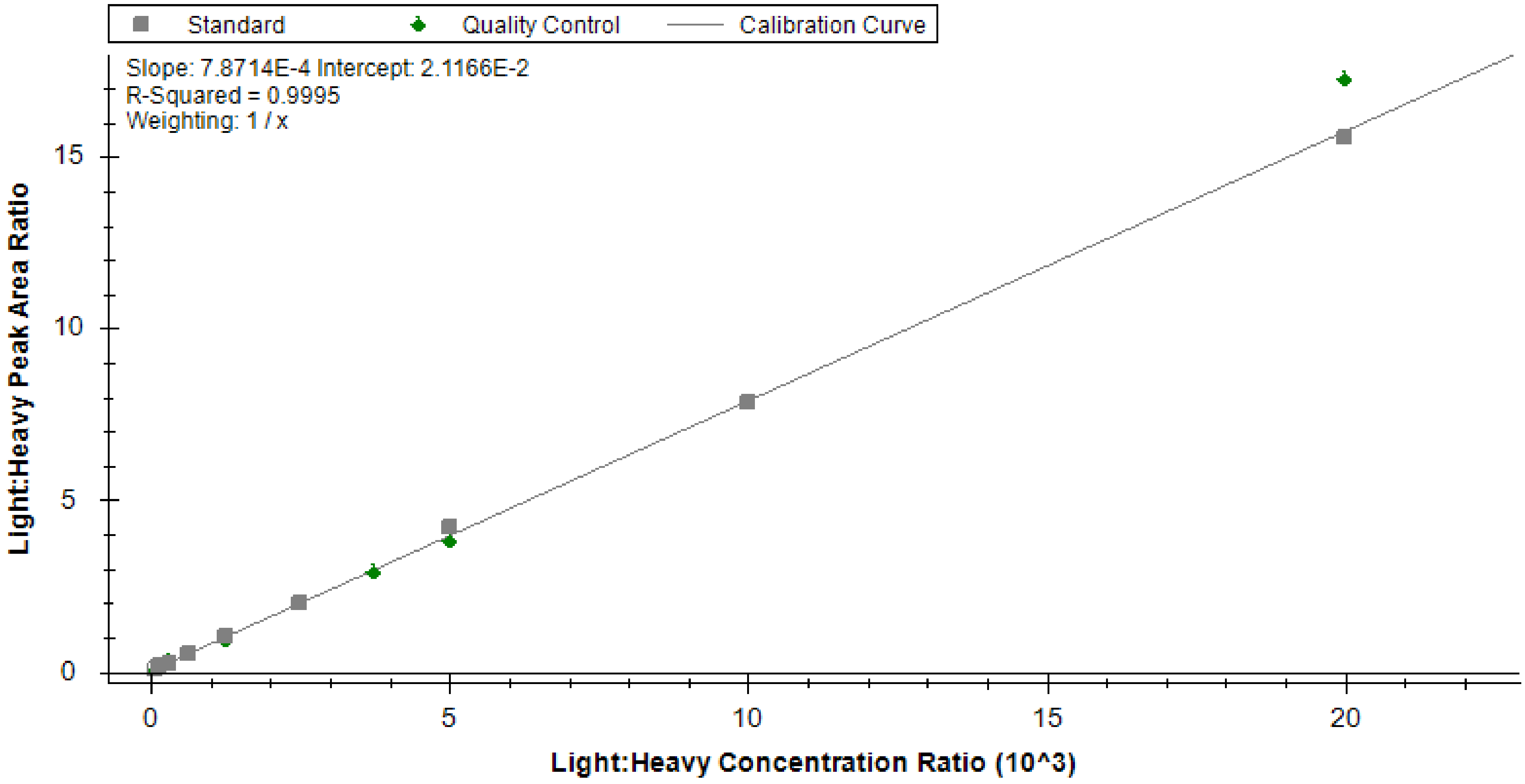

CEP3: LVYLTER

Quantifier ion: y5+, 447.2582++ → 681.3566+

AMR: 78.125 – 60000 pM

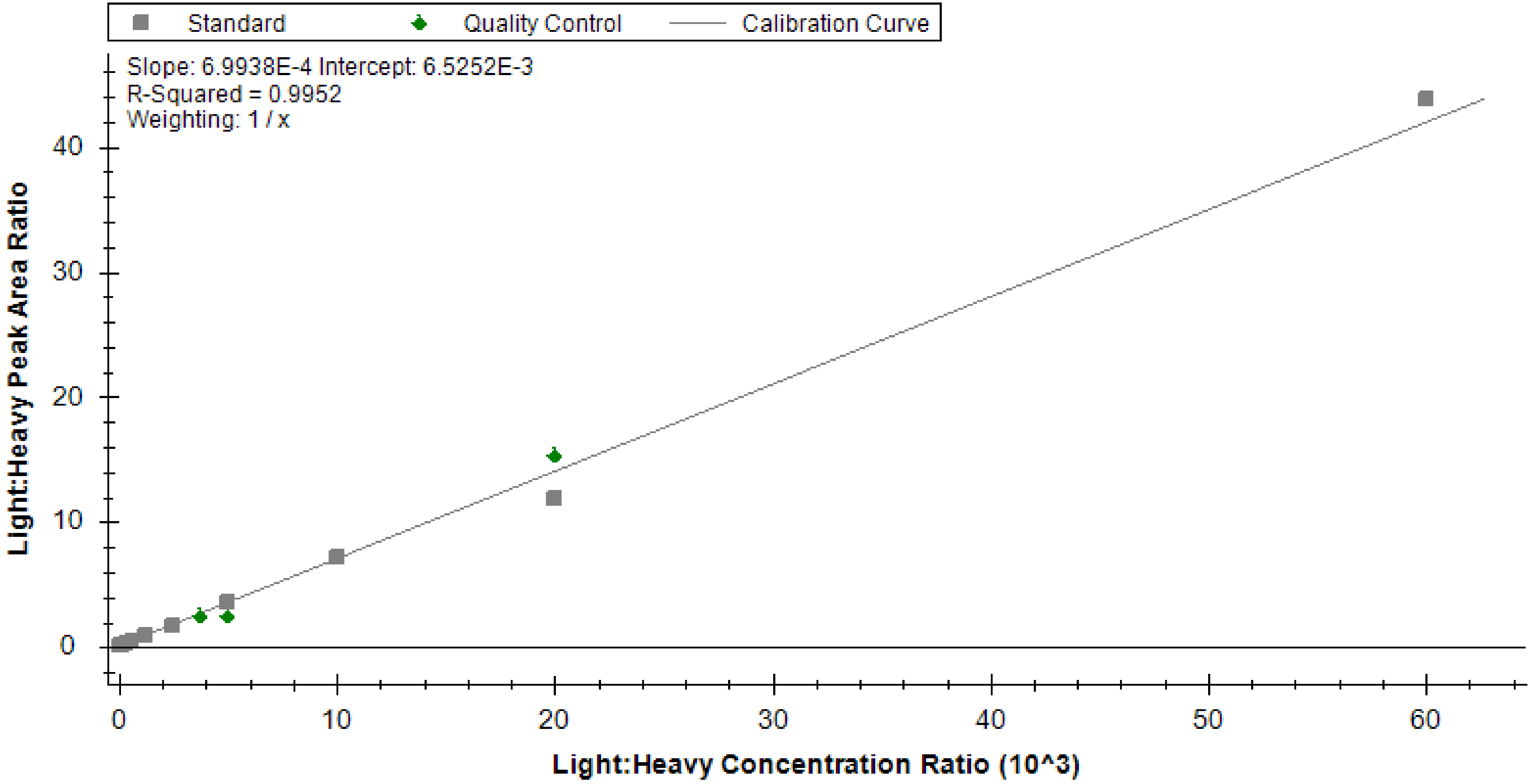

CSE4: YTPSELALYEIR

Quantifier ion: y6+, 727.8799++ → 764.4301+

AMR (Low range): 312.5 – 2500 pM

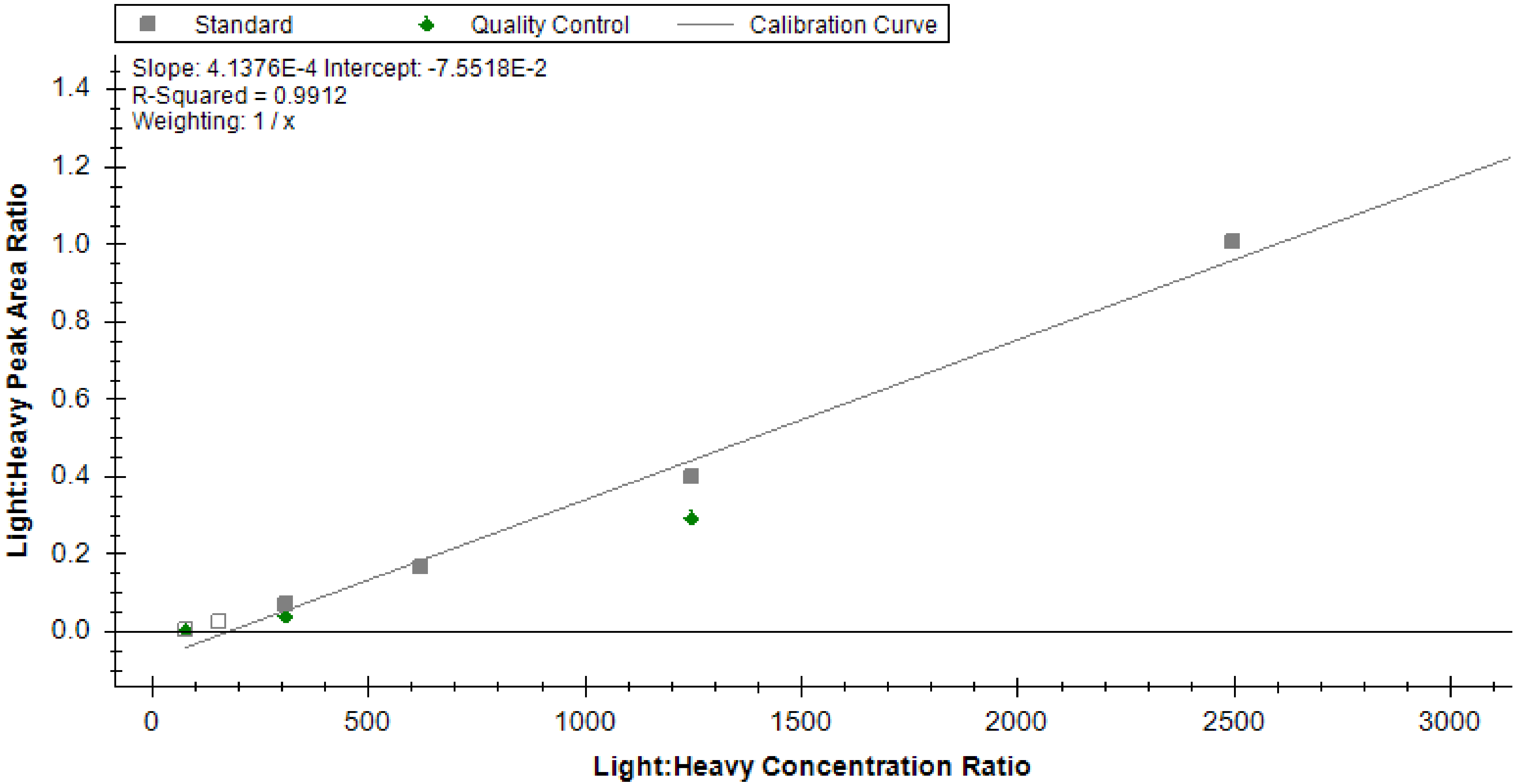

CSE4: YTPSELALYEIR

Quantifier ion: y6+, 727.8799++ → 764.4301+

AMR (High range): 2500 – 60000 pM

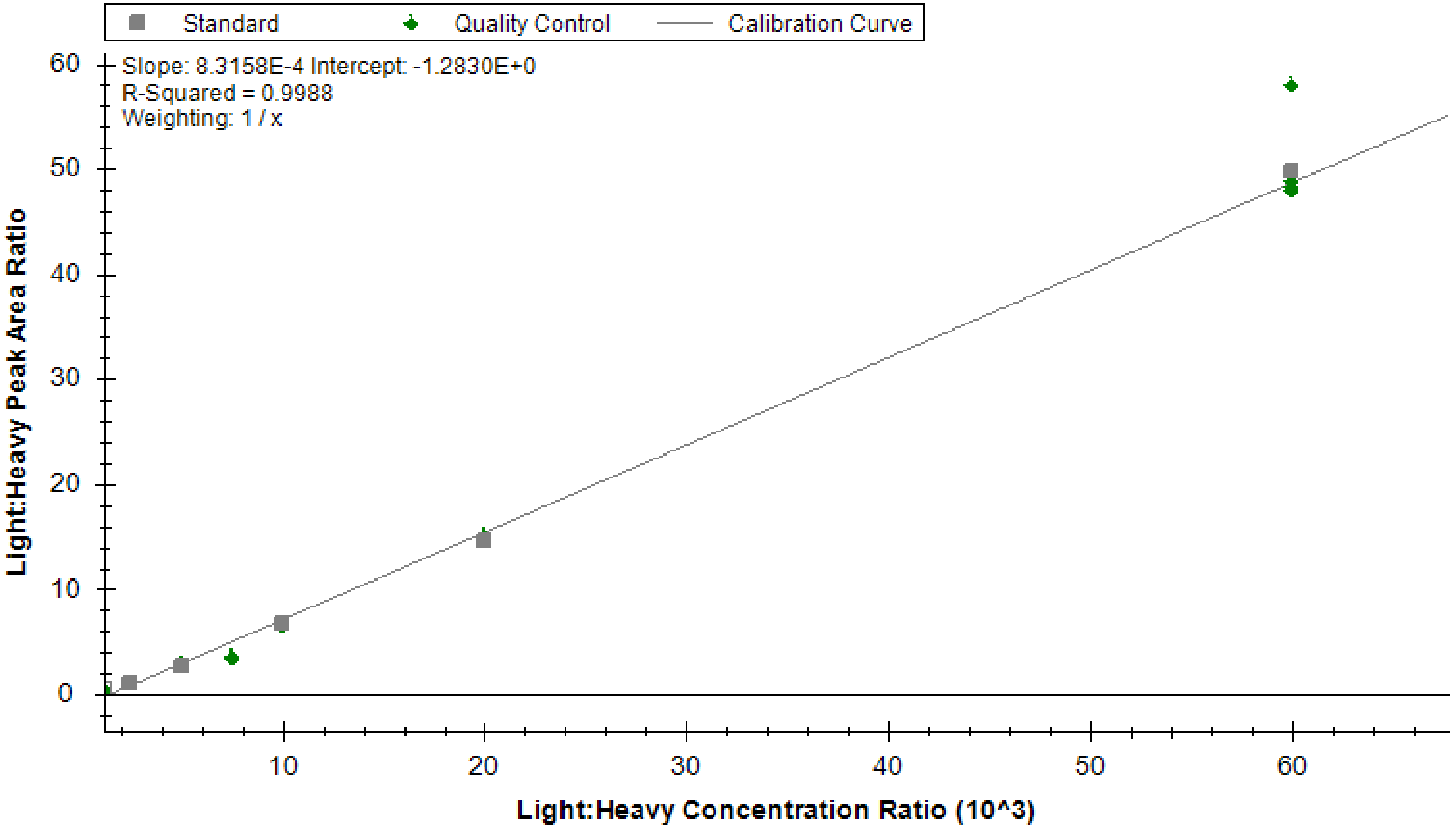

HTB2: HAVSEGTR

Quantifier ion: b2+, 428.7172++ → 209.1033+

AMR: 625 – 60000 pM

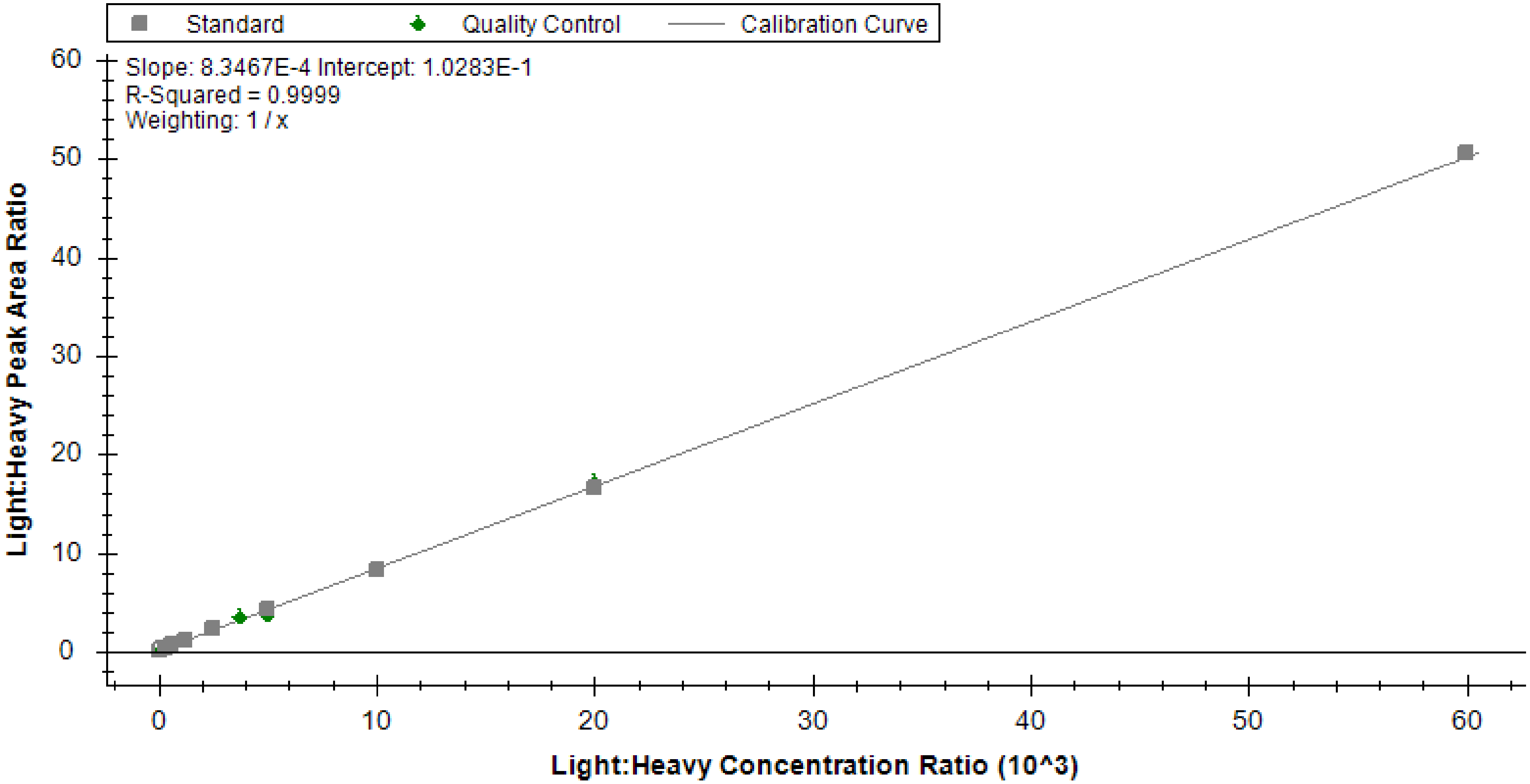

HTA2: AGLTFPVGR

Quantifier ion: y4+, 459.2638++ → 428.2616+

AMR: 78.125 – 60000 pM

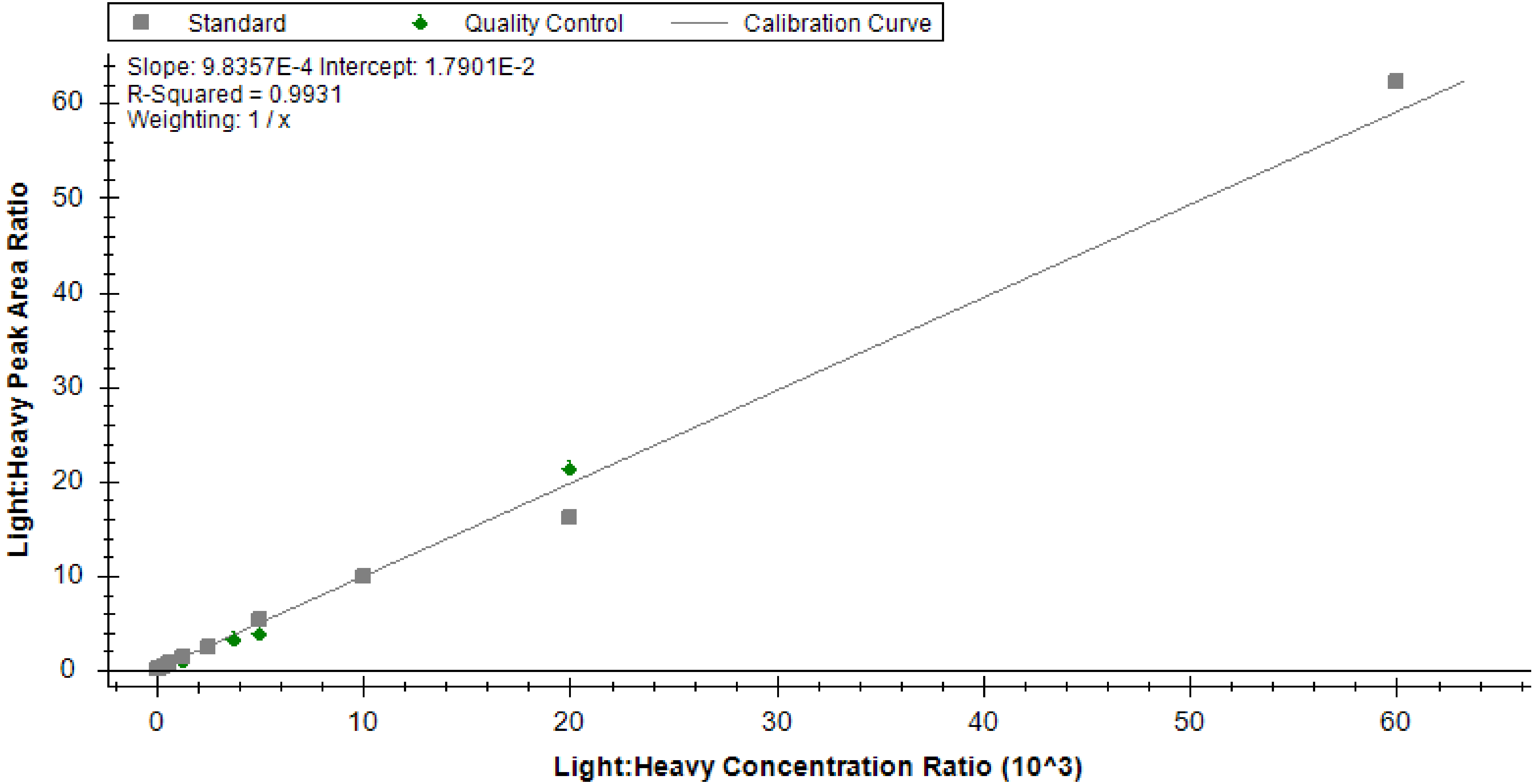

HHF1: ISGLIYEEVR

Quantifier ion: y5+, 589.8244++ → 695.3359+

AMR: 1250 – 60000 pM

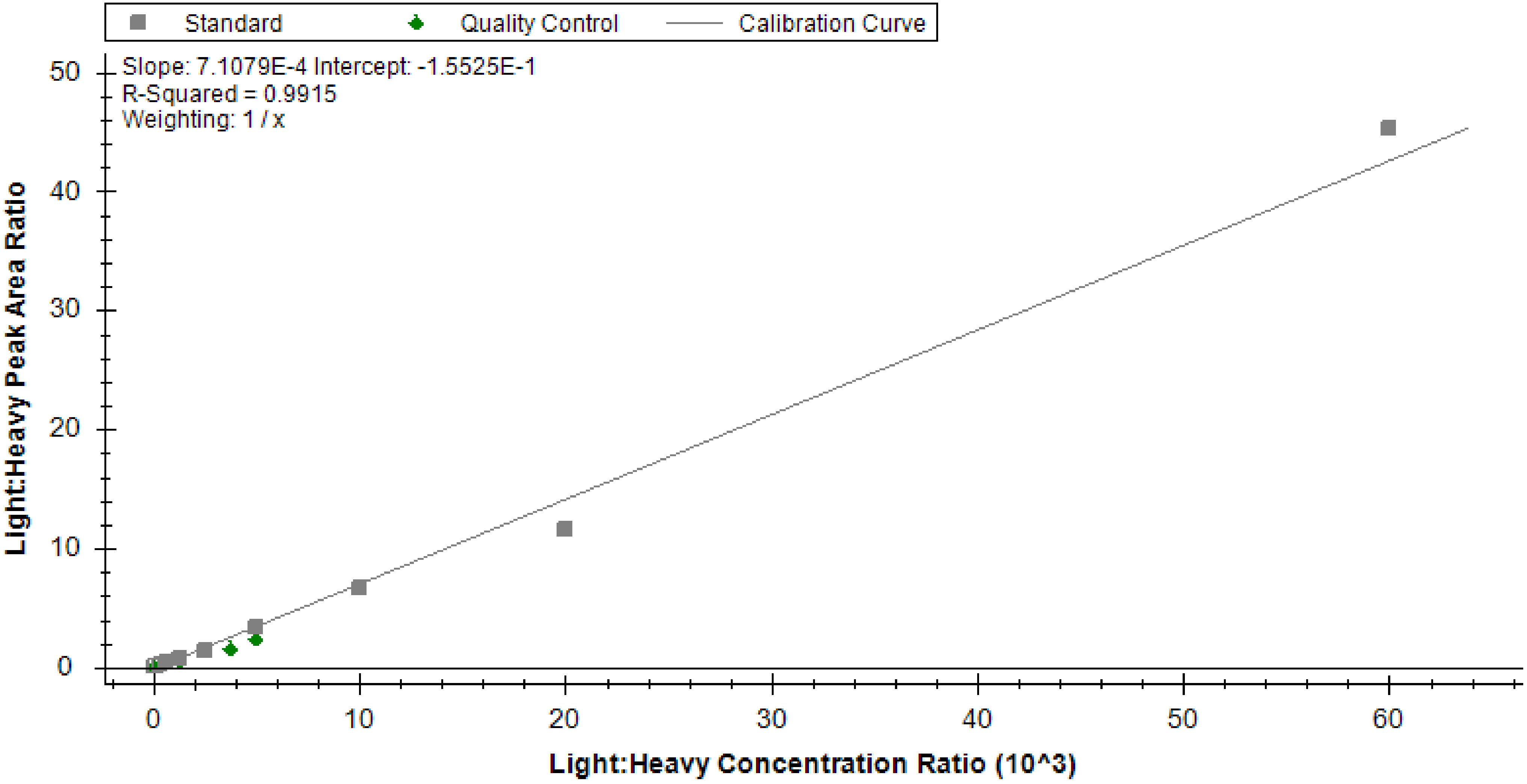

HHT1: STELLIR

Quantifier ion: y5+, 416.2504++ → 643.4137+

AMR: 312.5 – 60000 pM

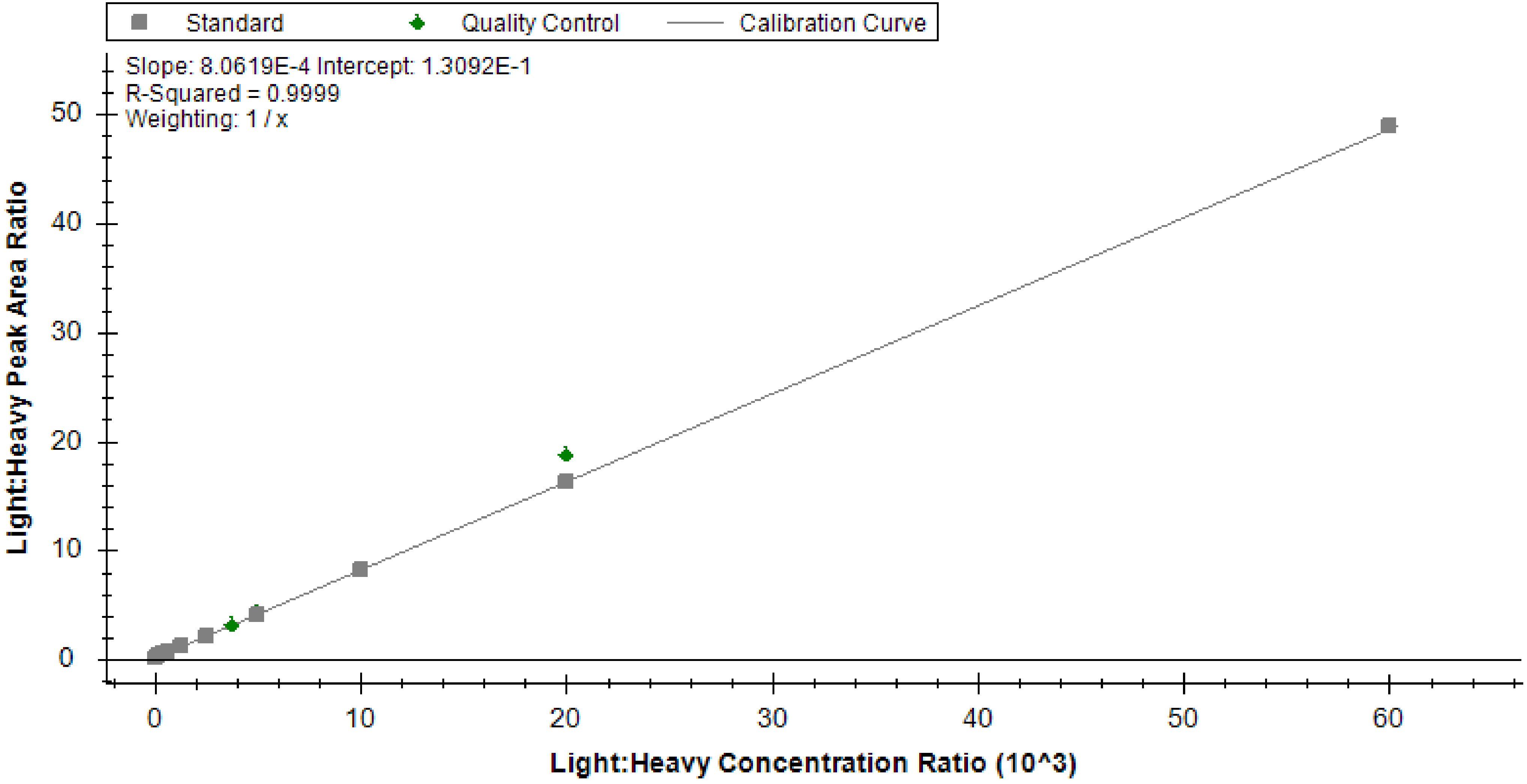

MIF2-1: YSLDTSESPSVR

Quantifier ion: y5+, 670.8201++ → 545.3042+

AMR: 625 – 60000 pM

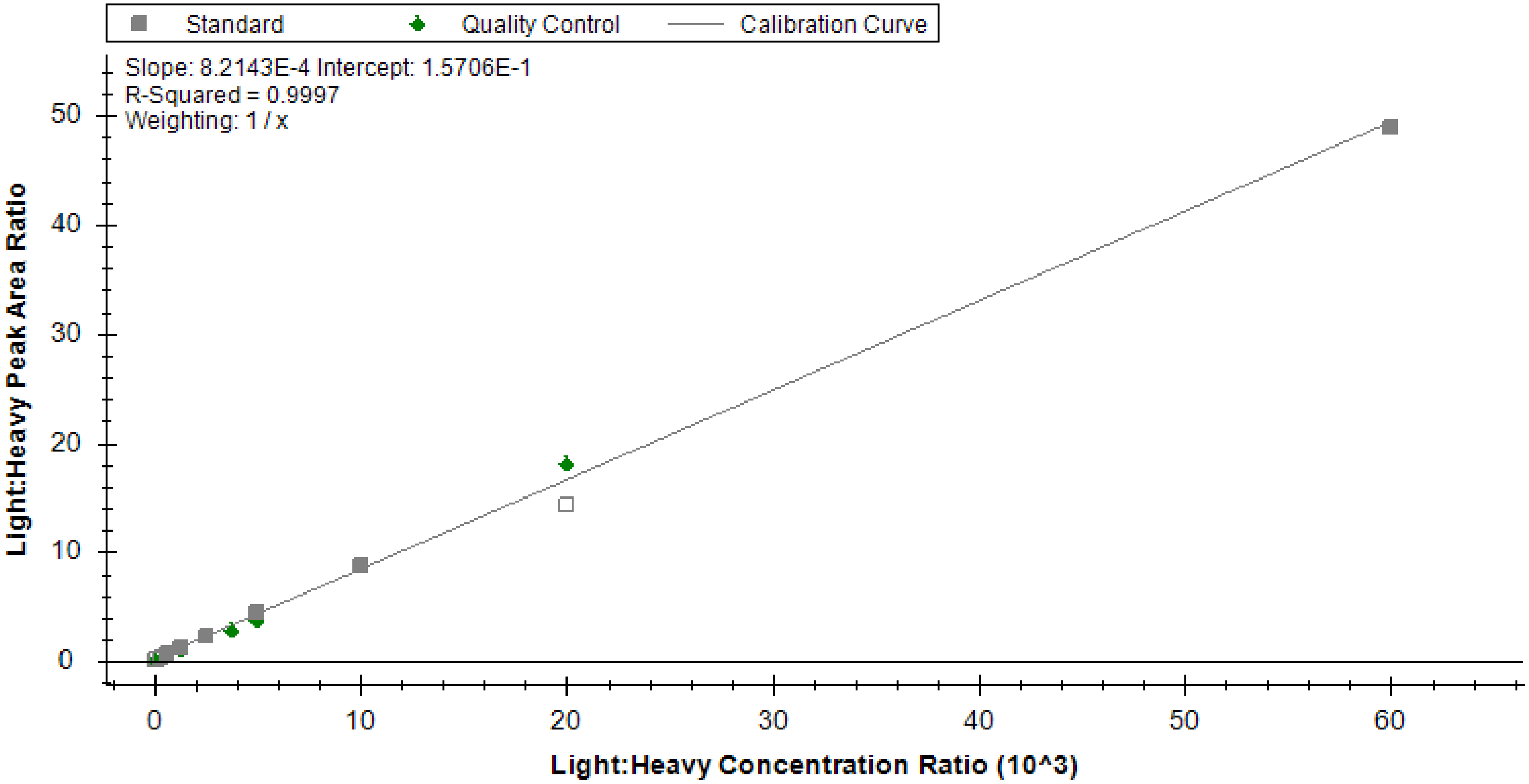

MIF2-2: VAPLQYWR

Quantifier ion: y5+, 516.7849++ → 765.4042+

AMR: 78.125 – 60000 pM

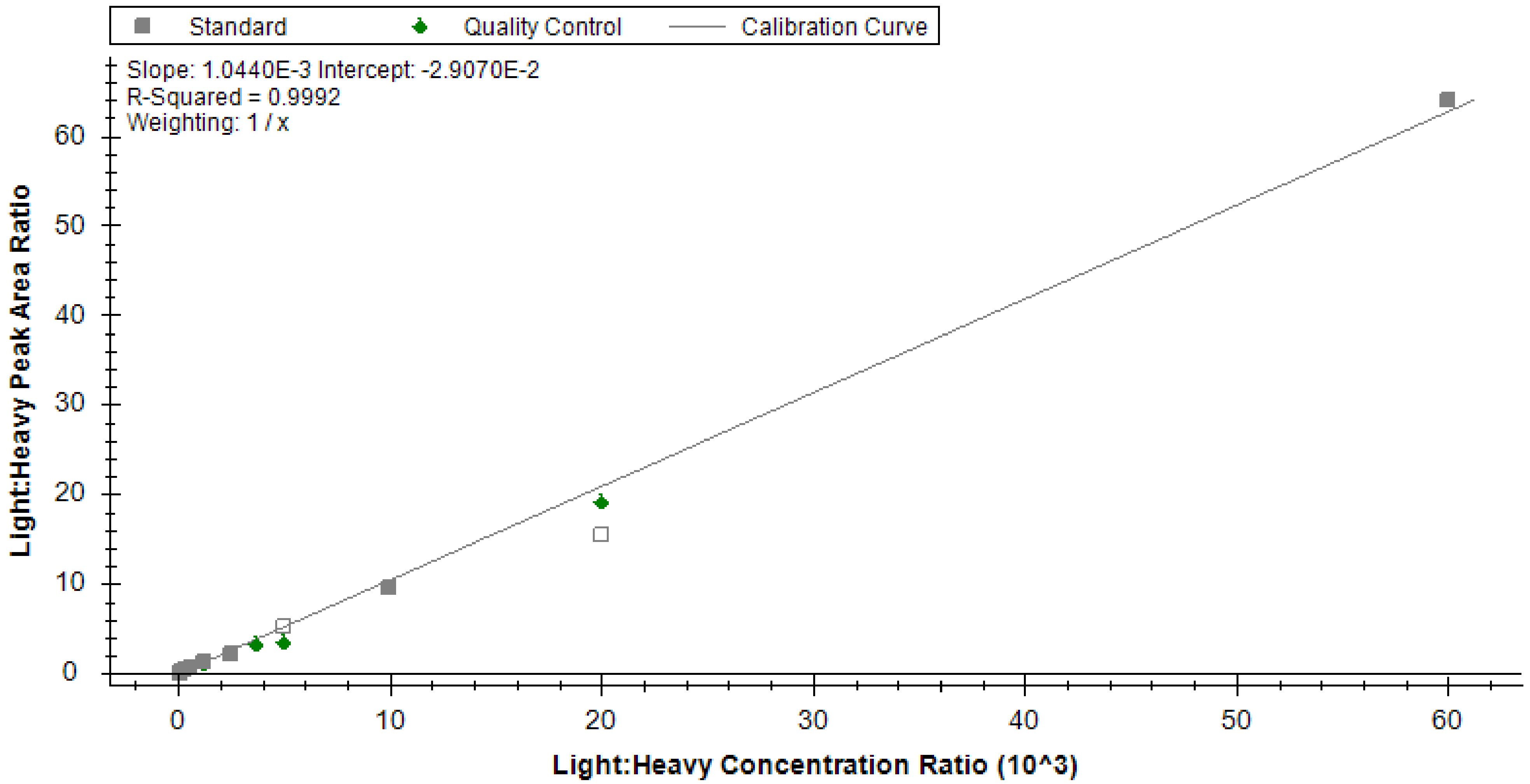

CBF2: EENIVNEDGPNTSR

Quantifier ion: y9+, 787.3581++ → 989.4283+

AMR: 625 – 60000 pM

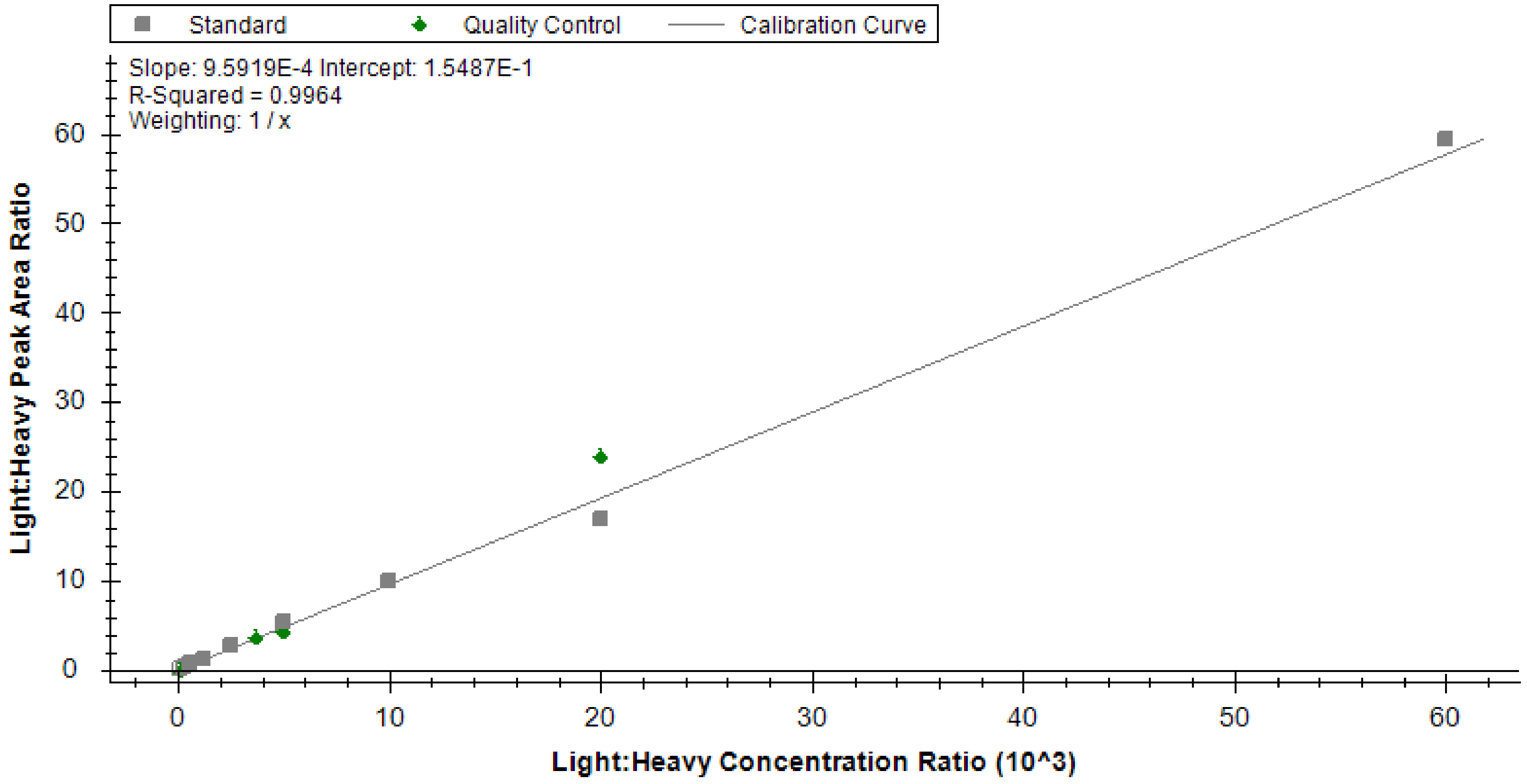

MCM21: IDDISTSDR

Quantifier ion: b3+, 511.2435++ → 344.1452+

AMR: 78.125 – 60000 pM

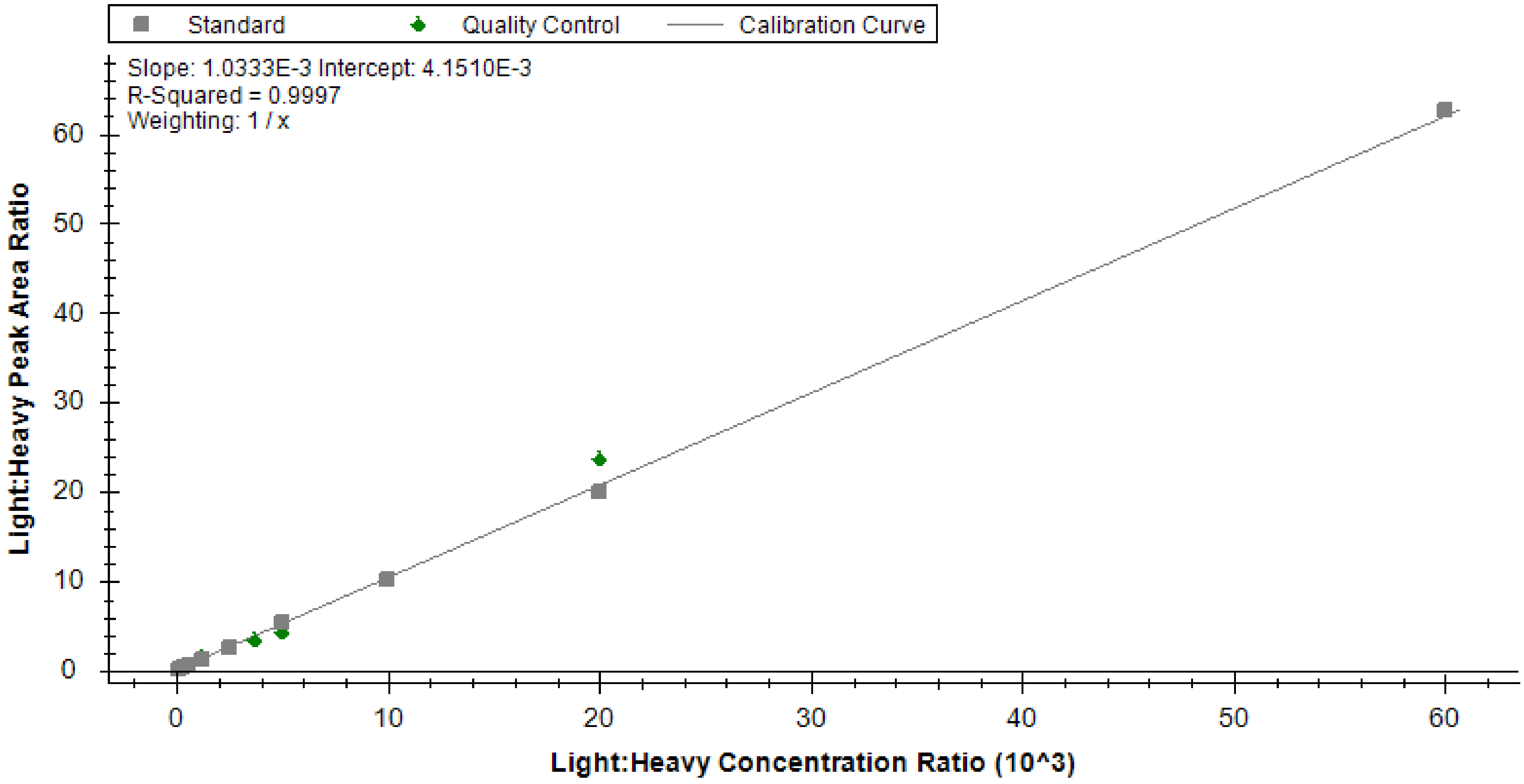

CTF19: QQLSLLDDDQVR

Quantifier ion: y6+, 715.3677++ → 747.3268+

AMR: 78.125 – 60000 pM

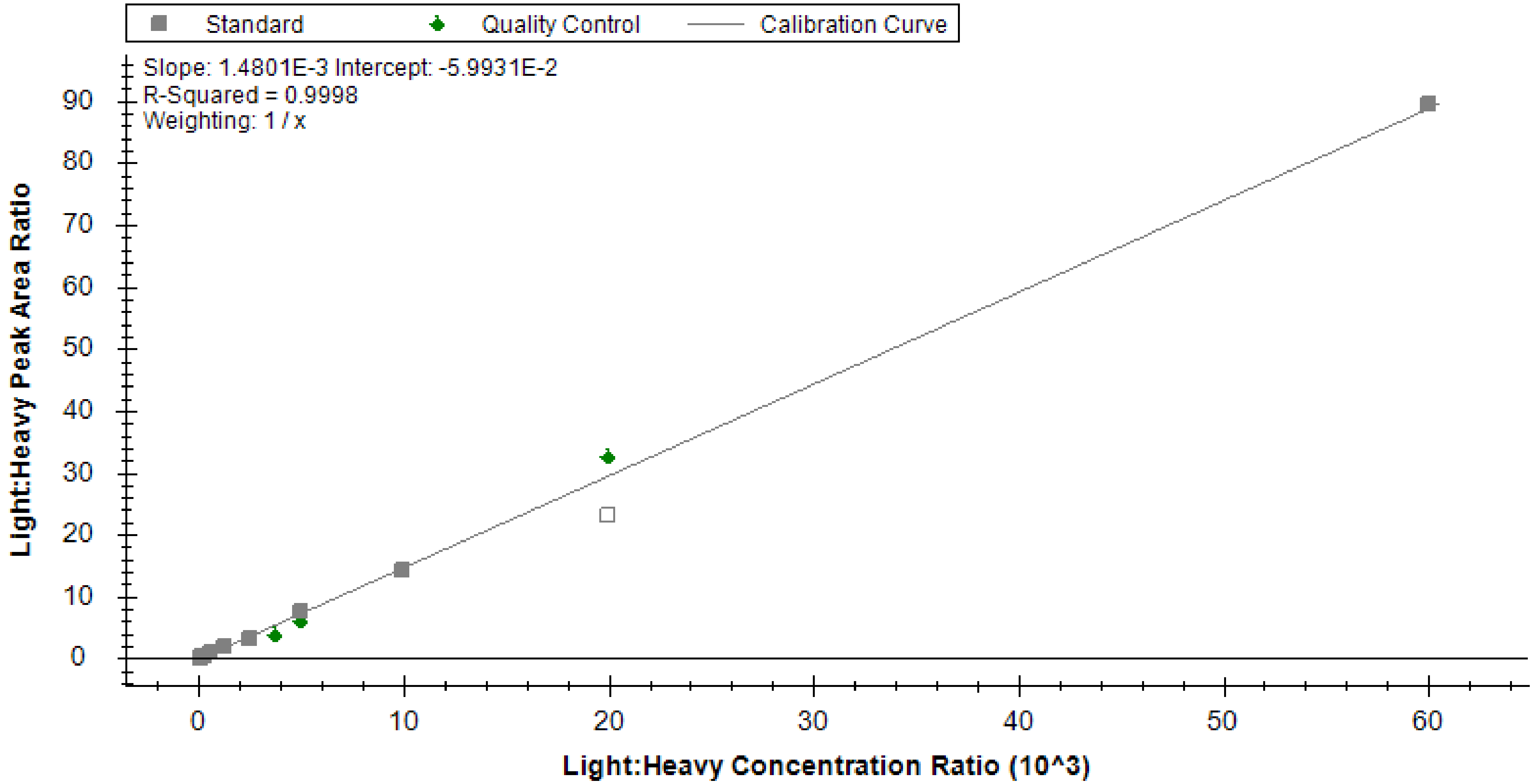

CTF3: DAPGSATLILQR

Quantifier ion: y9+, 621.3461++ → 958.5680+

AMR: 78.125 – 60000 pM

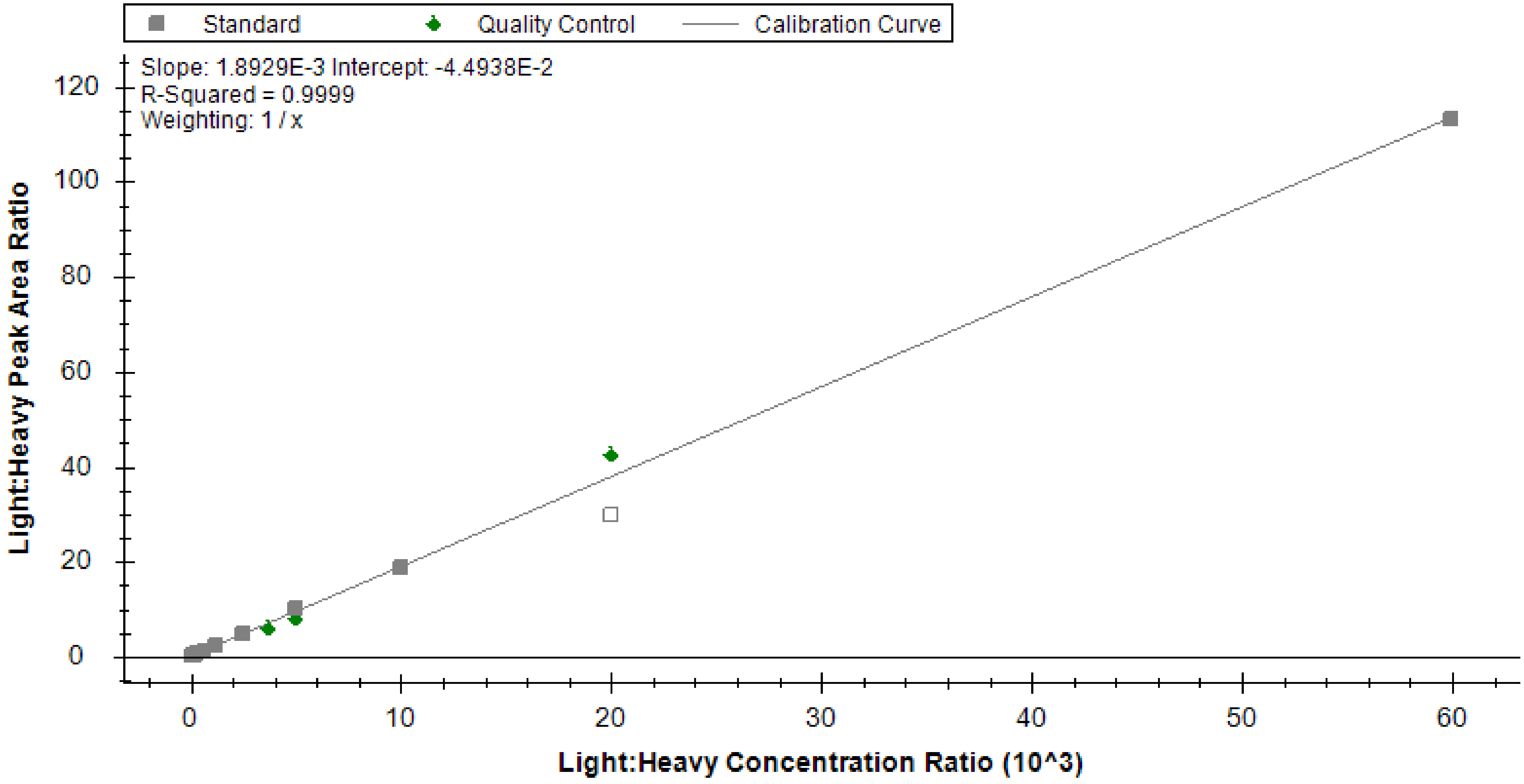

IML3: ESIVTSTR

Quantifier ion: y4+, 446.7404++ → 464.2463+

AMR: 78.125 – 60000 pM

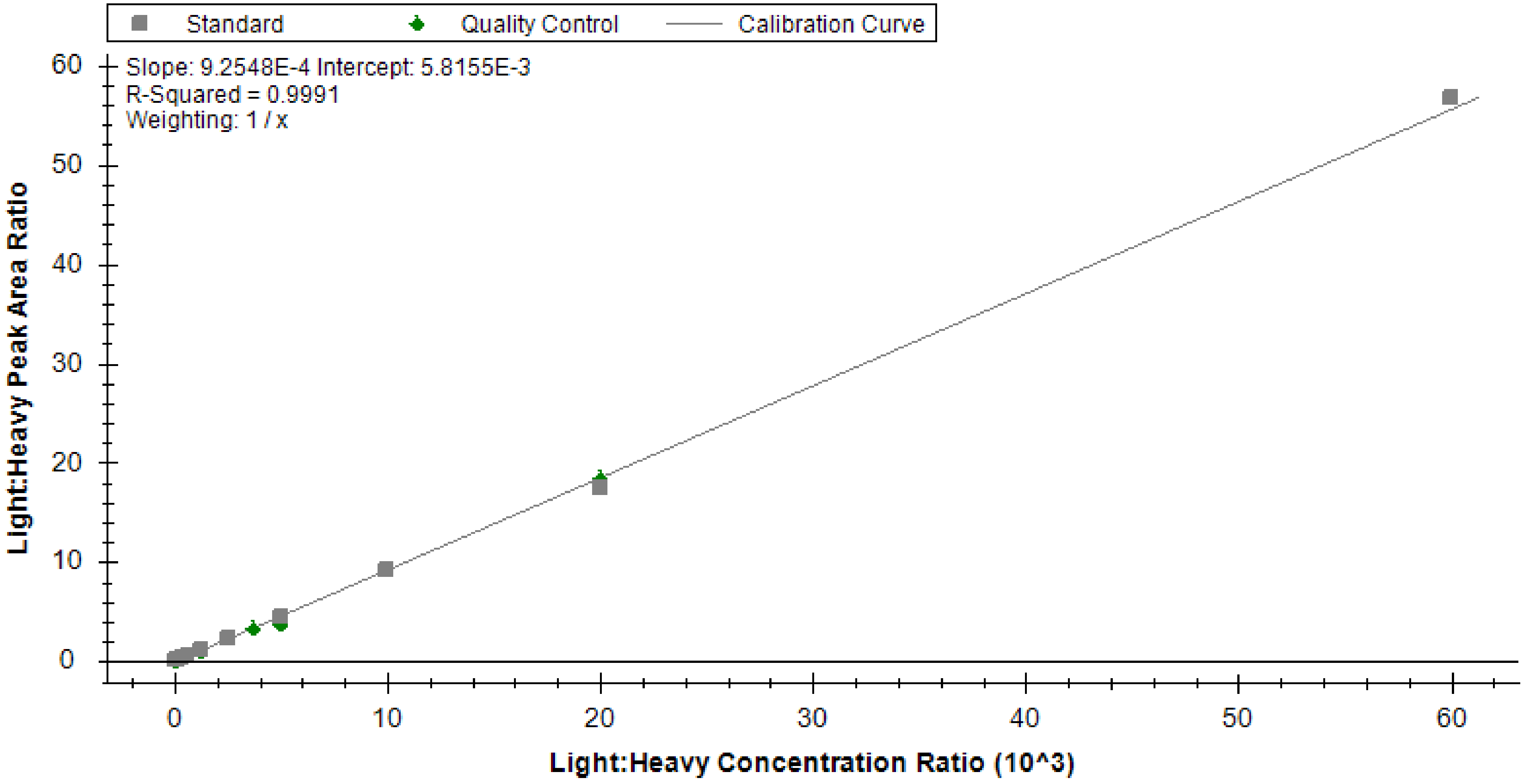

CHL4: NEDSGEPVYISR

Quantifier ion: y6+, 683.3177++ → 734.4196+

AMR: 78.125 – 60000 pM

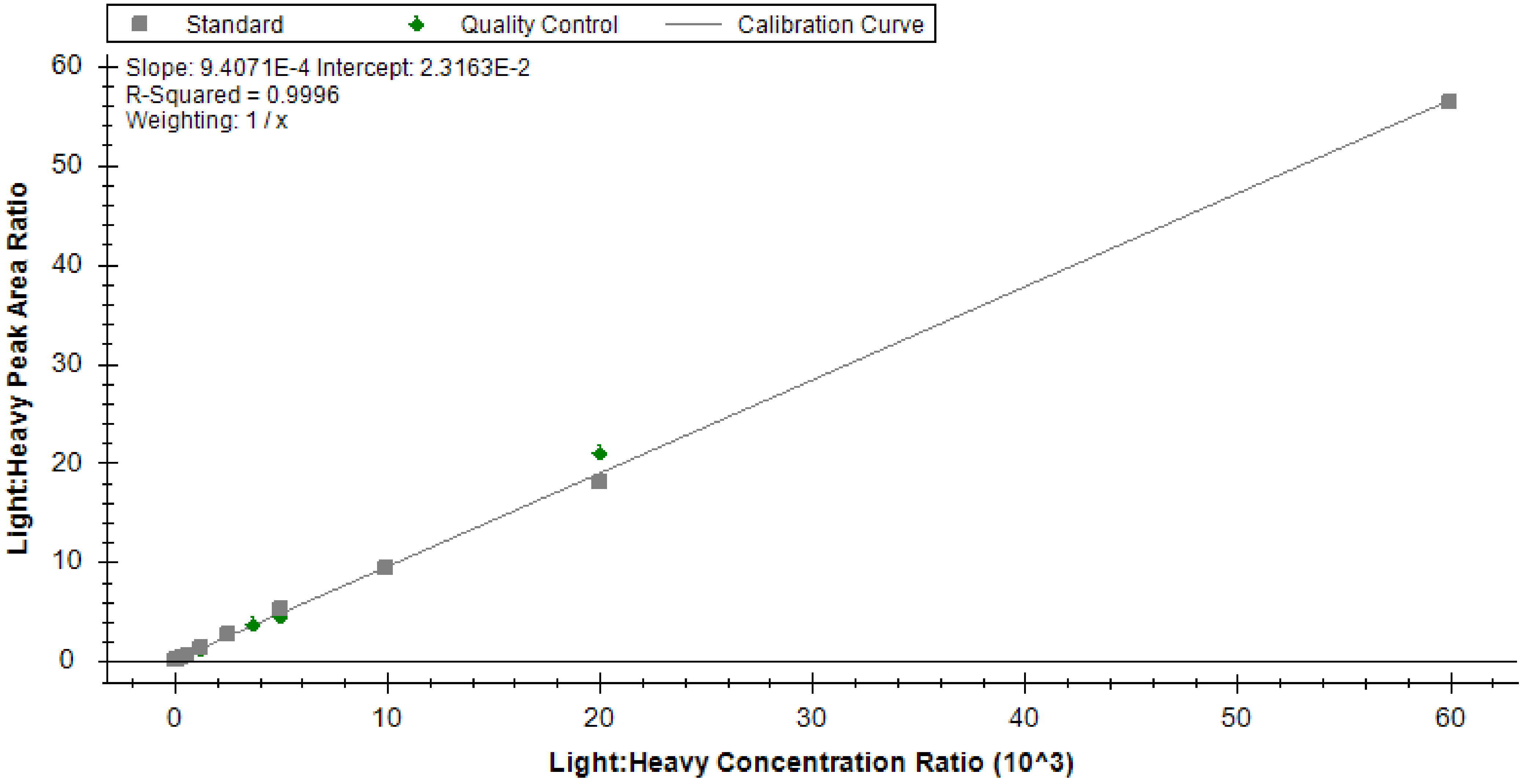

MTW1: IPEEYLDANVFR

Quantifier ion: y11++, 733.3697++ → 676.8277++

AMR (Low range): 312.5 – 2500 pM

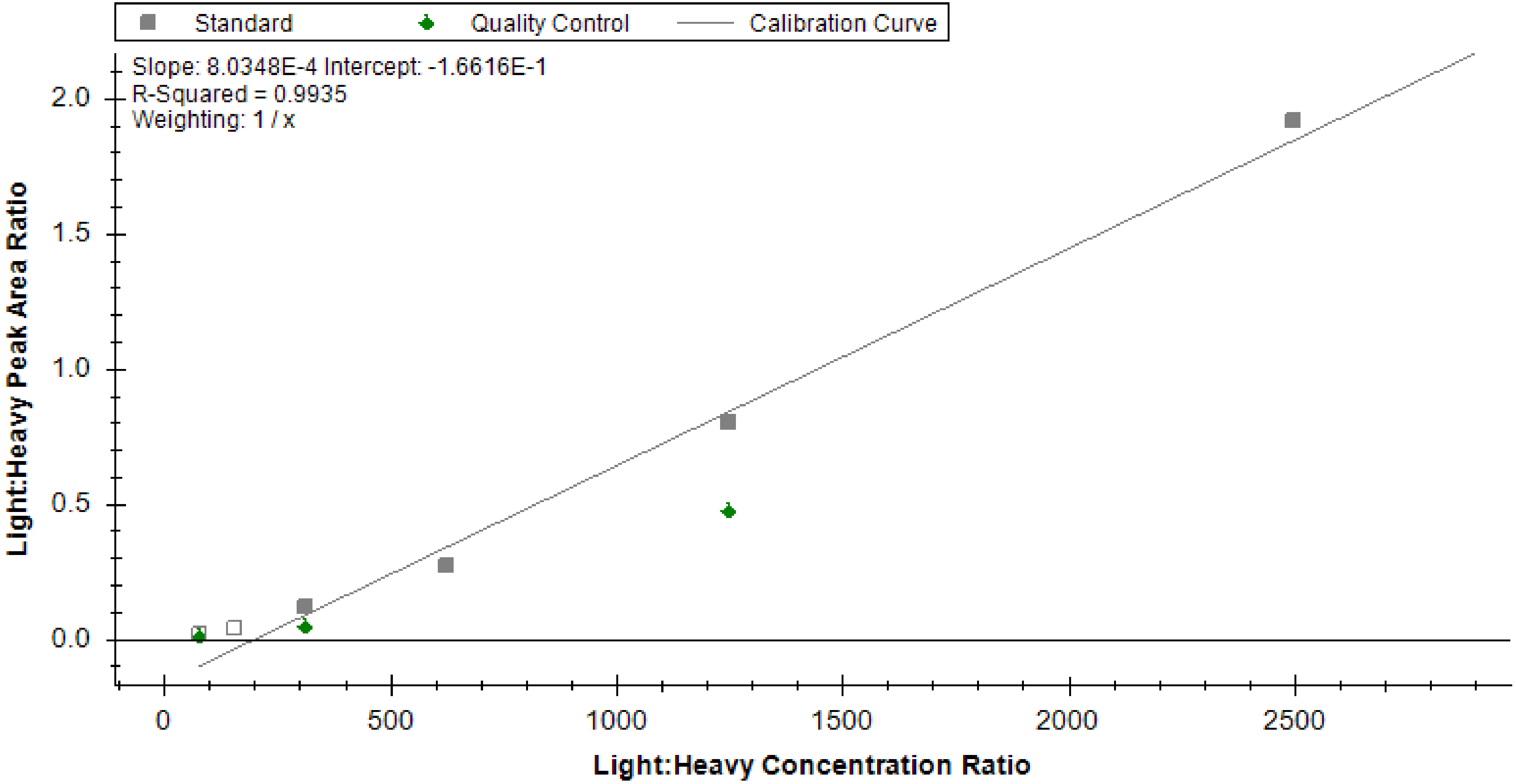

MTW1: IPEEYLDANVFR

Quantifier ion: y11++, 733.3697++ → 676.8277++

AMR (High range): 2500 – 60000 pM

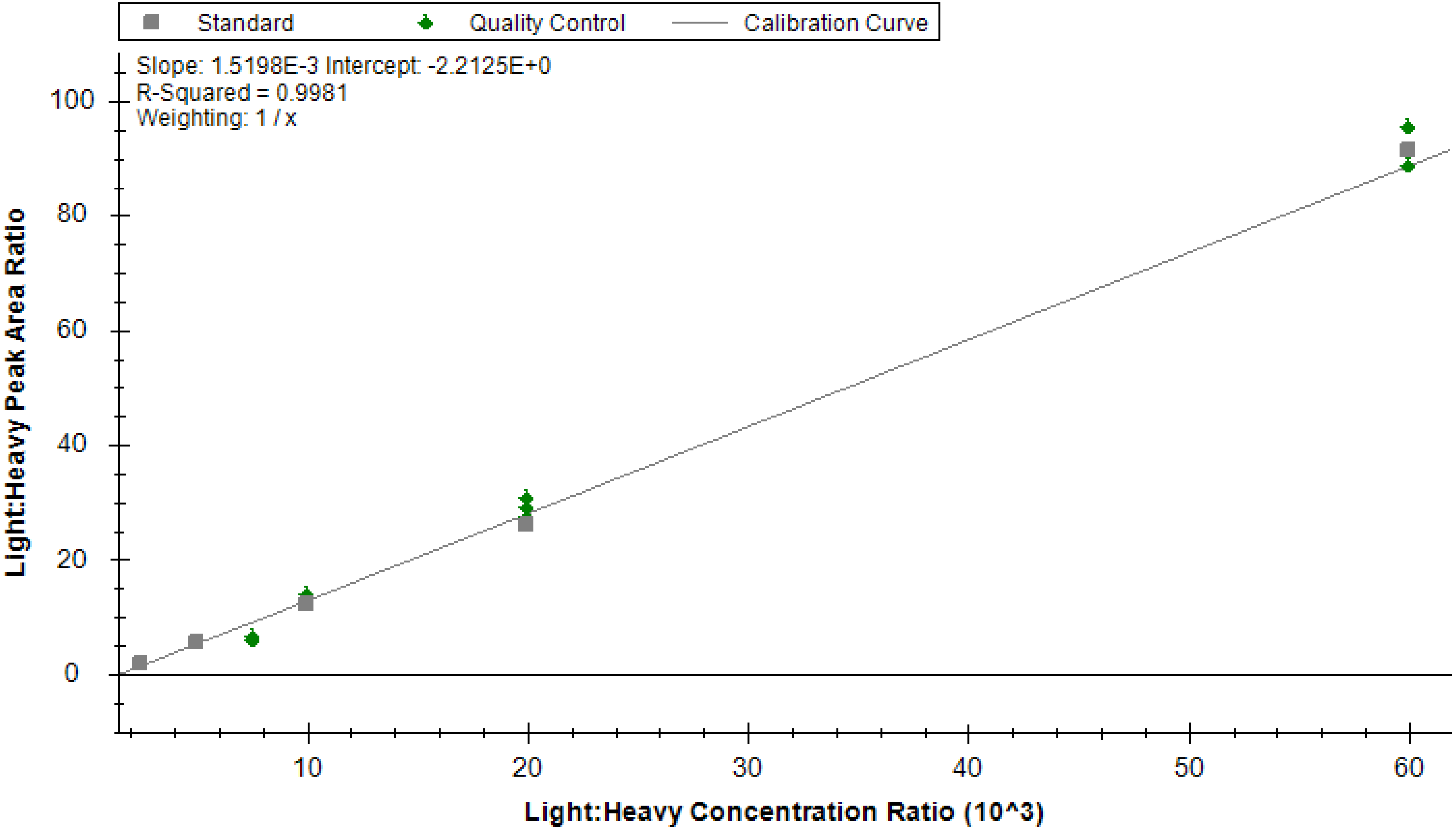

CNN1: SFLQDLSQVLAR

Quantifier ion: y2+, 688.8803++ → 246.1561+

AMR: 2500 – 20000 pM

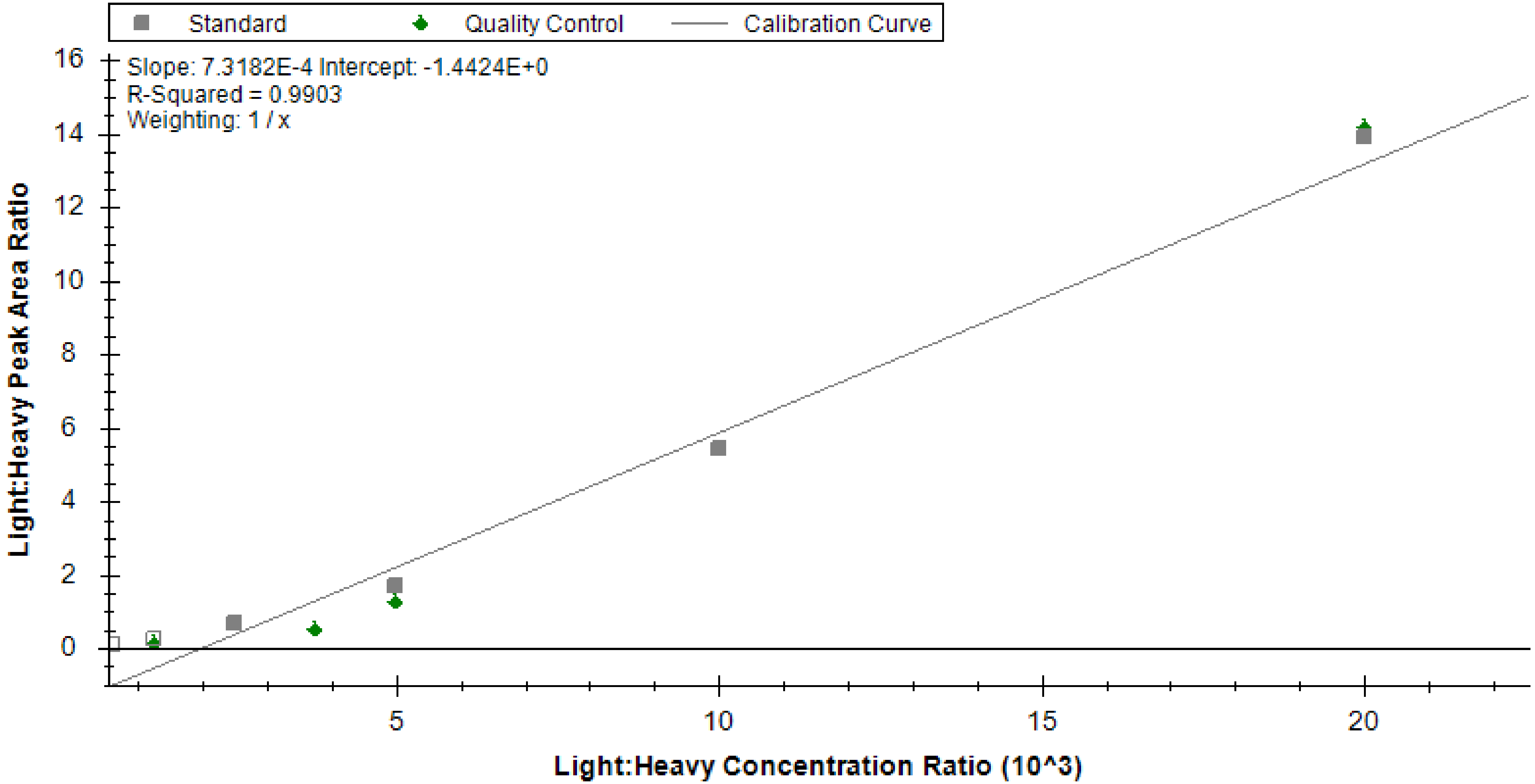

NKP1: EIYDNESELR

Quantifier ion: y3+, 634.2937++ → 417.2456+

AMR: 625 – 60000 pM

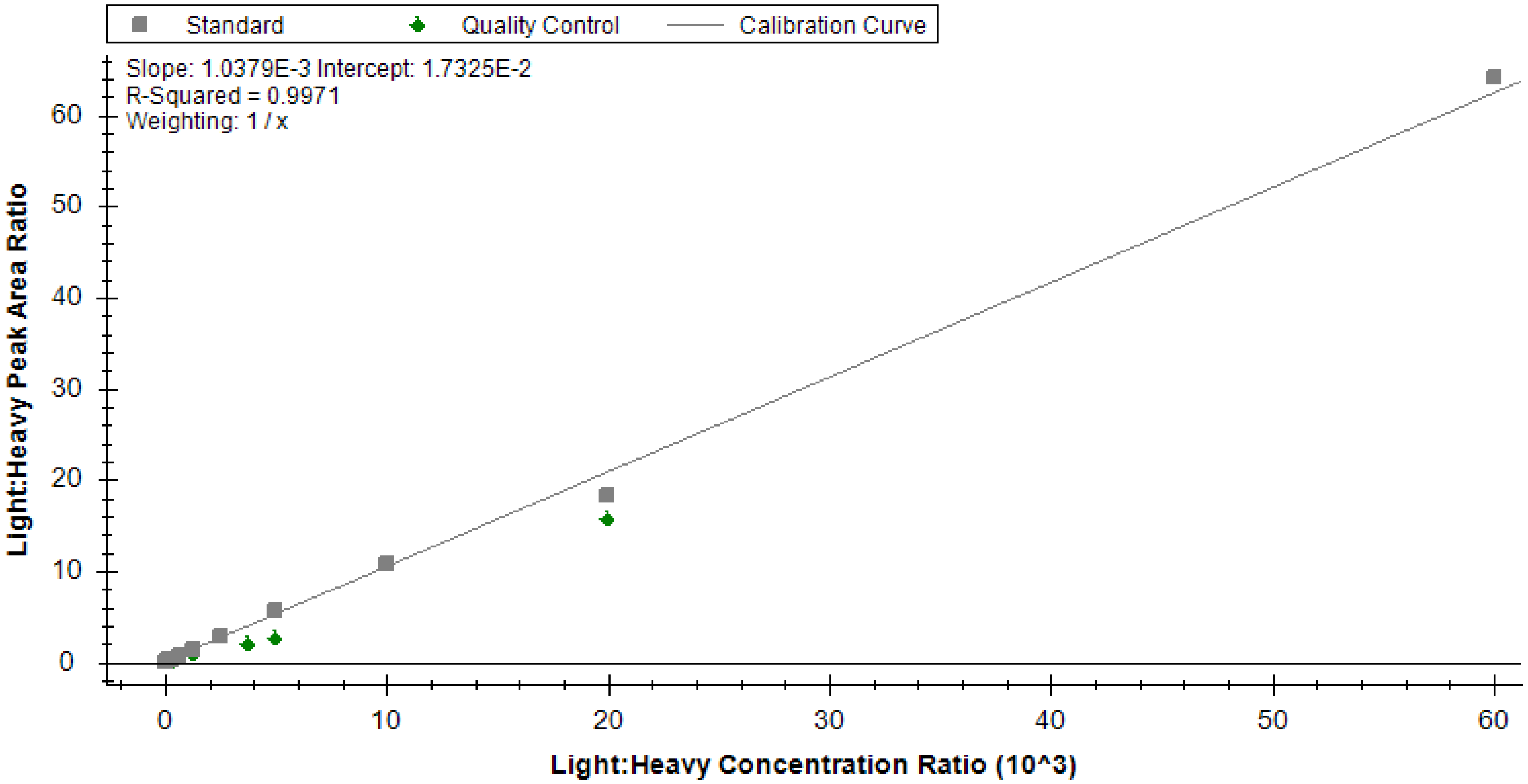

NKP2: VTSELEAR

Quantifier ion: y6+, 452.7404++ → 704.3573+

AMR: 312.5 – 60000 pM

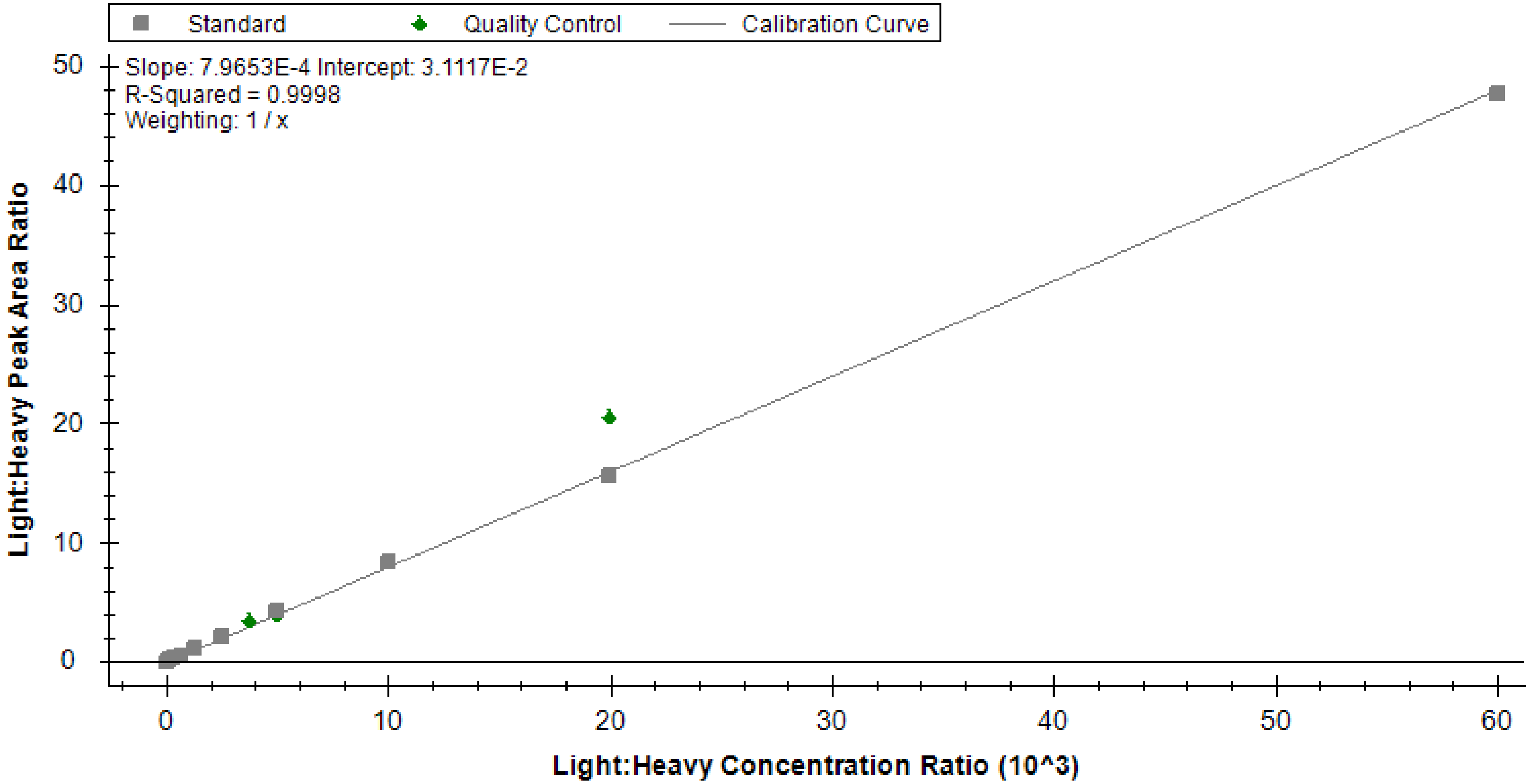

NDC80: QYDSSIQNLTR

Quantifier ion: y9+, 662.8282++ → 1033.5273+

AMR: 78.125 – 60000 pM

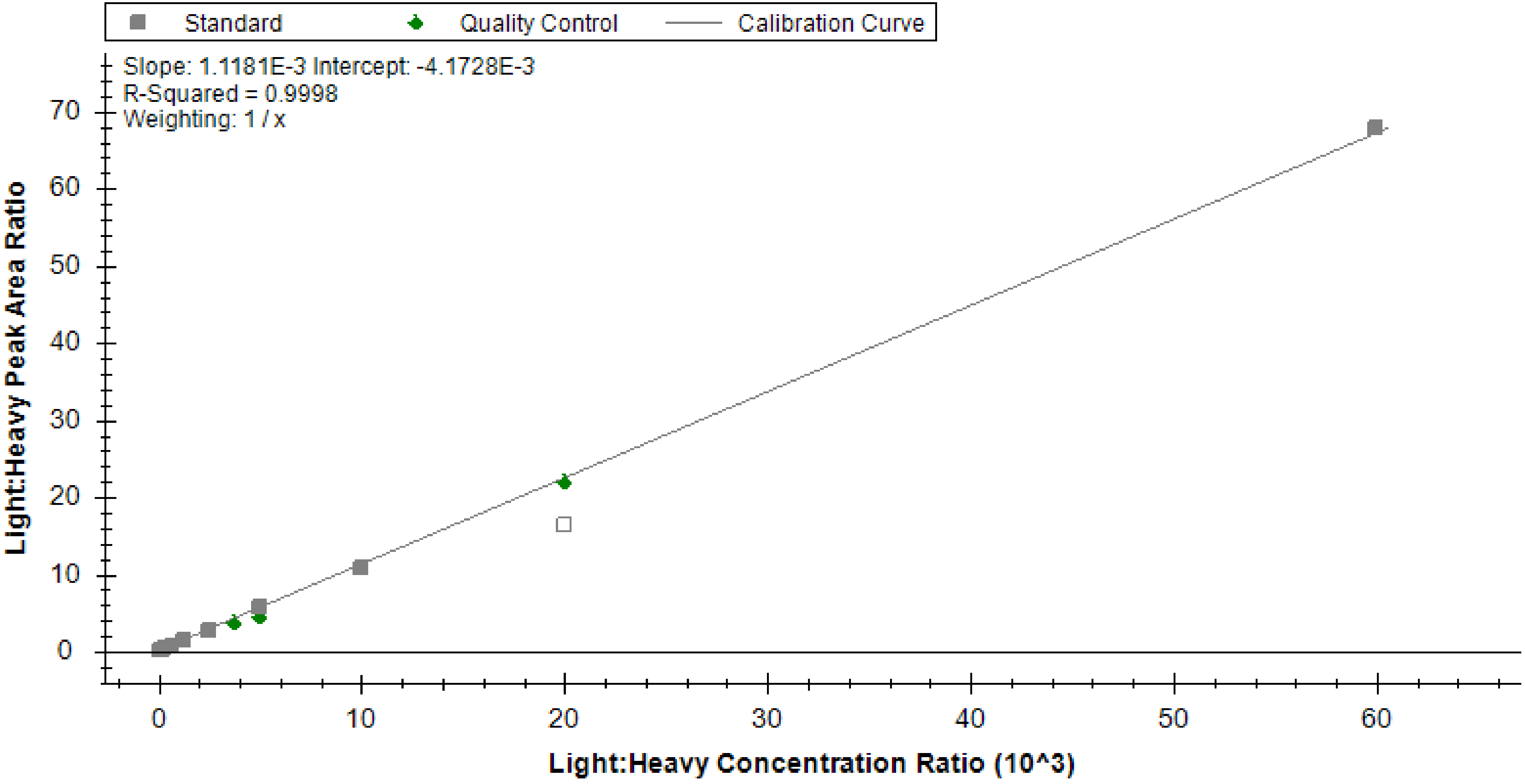

DSN1: ILDNTENYDDTELR

Quantifier ion: y9+, 855.8945++ → 1154.4960+

AMR: 78.125 – 60000 pM

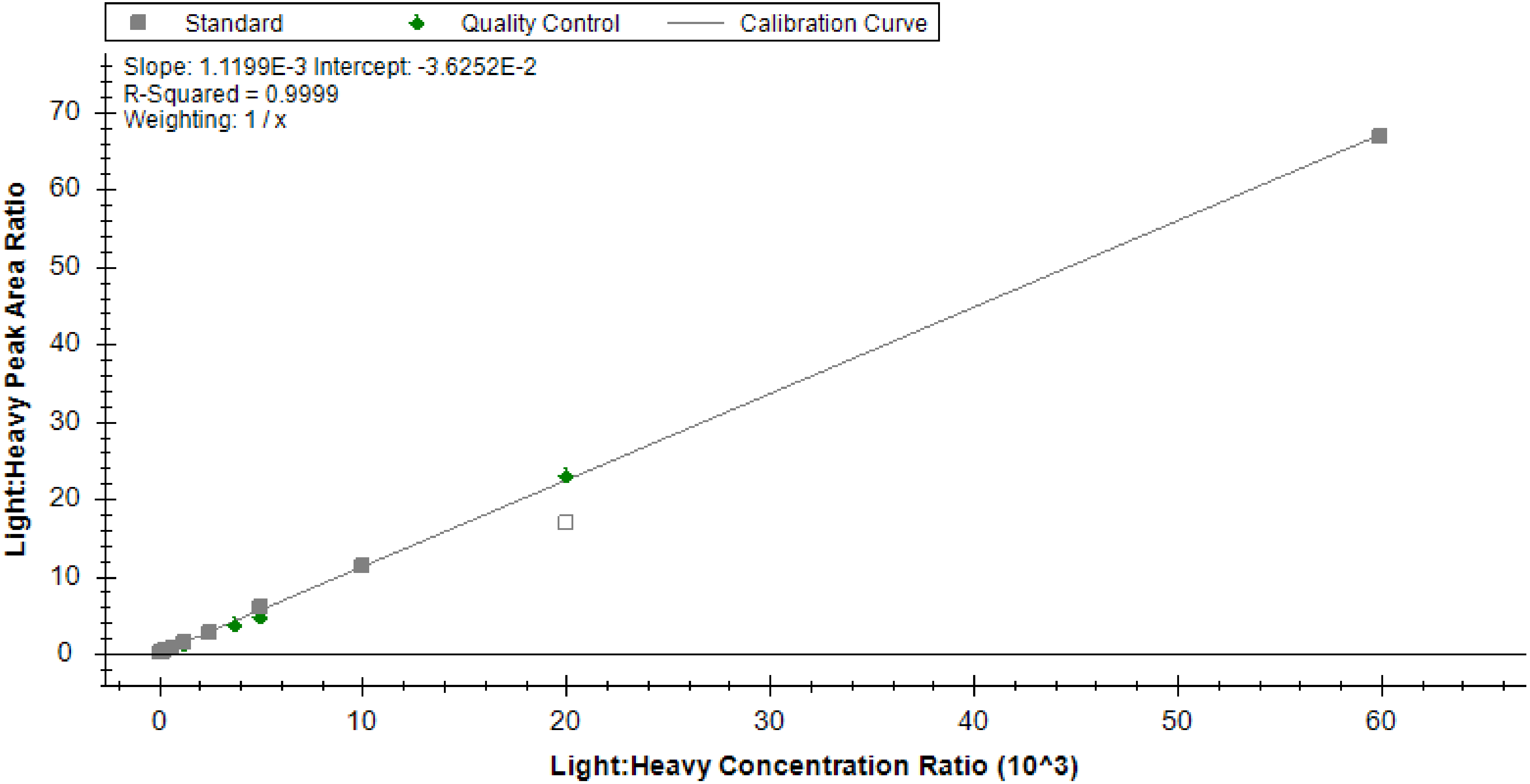

SPC105: VHISTQQDYSPSR

Quantifier ion: y5+, 506.5829++ → 609.2991+

AMR: 156.25 – 60000 pM

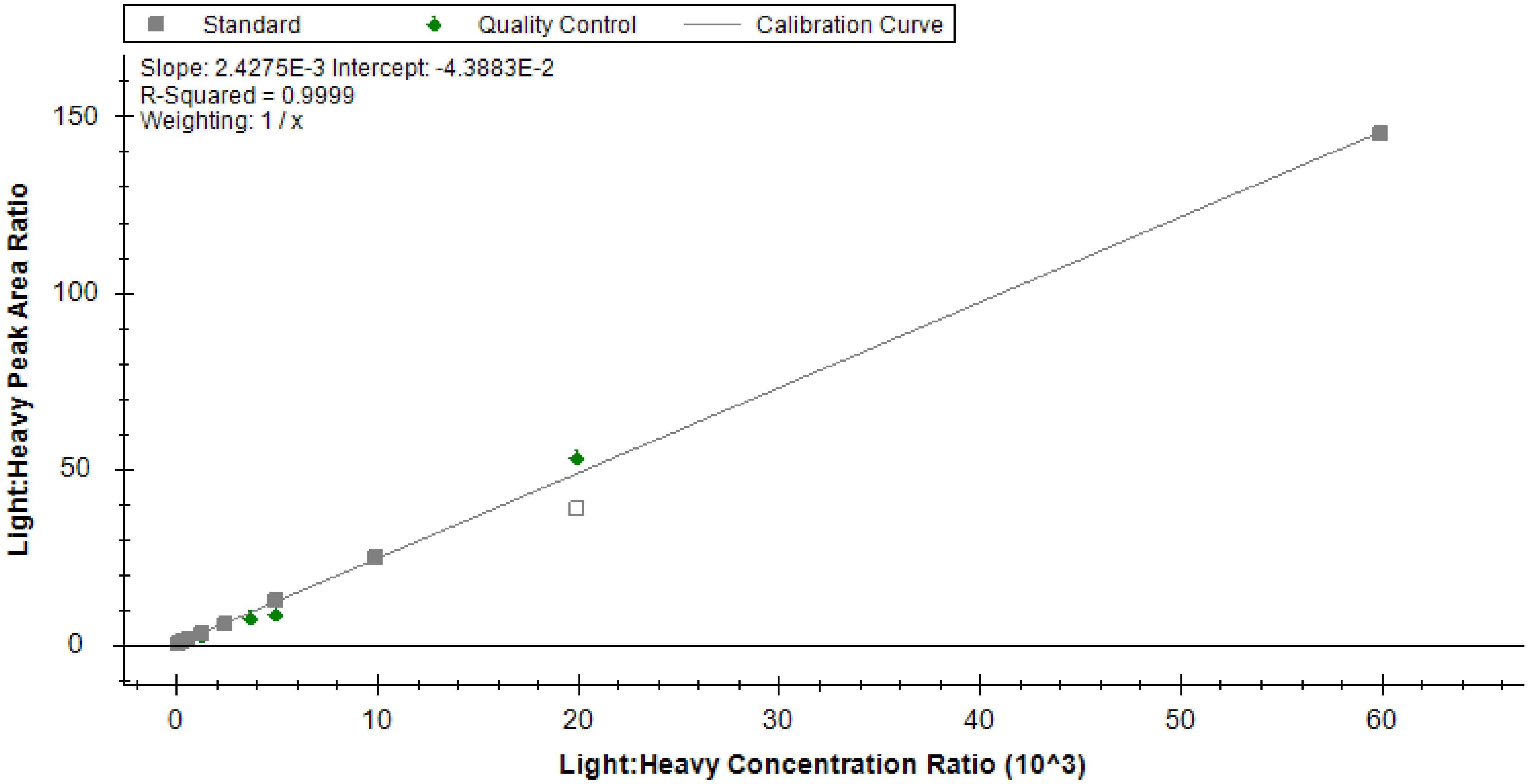

OKP1: VIQAEYR

Quantifier ion: y5+, 439.7402++ → 666.3206+

AMR: 156.25 – 60000 pM

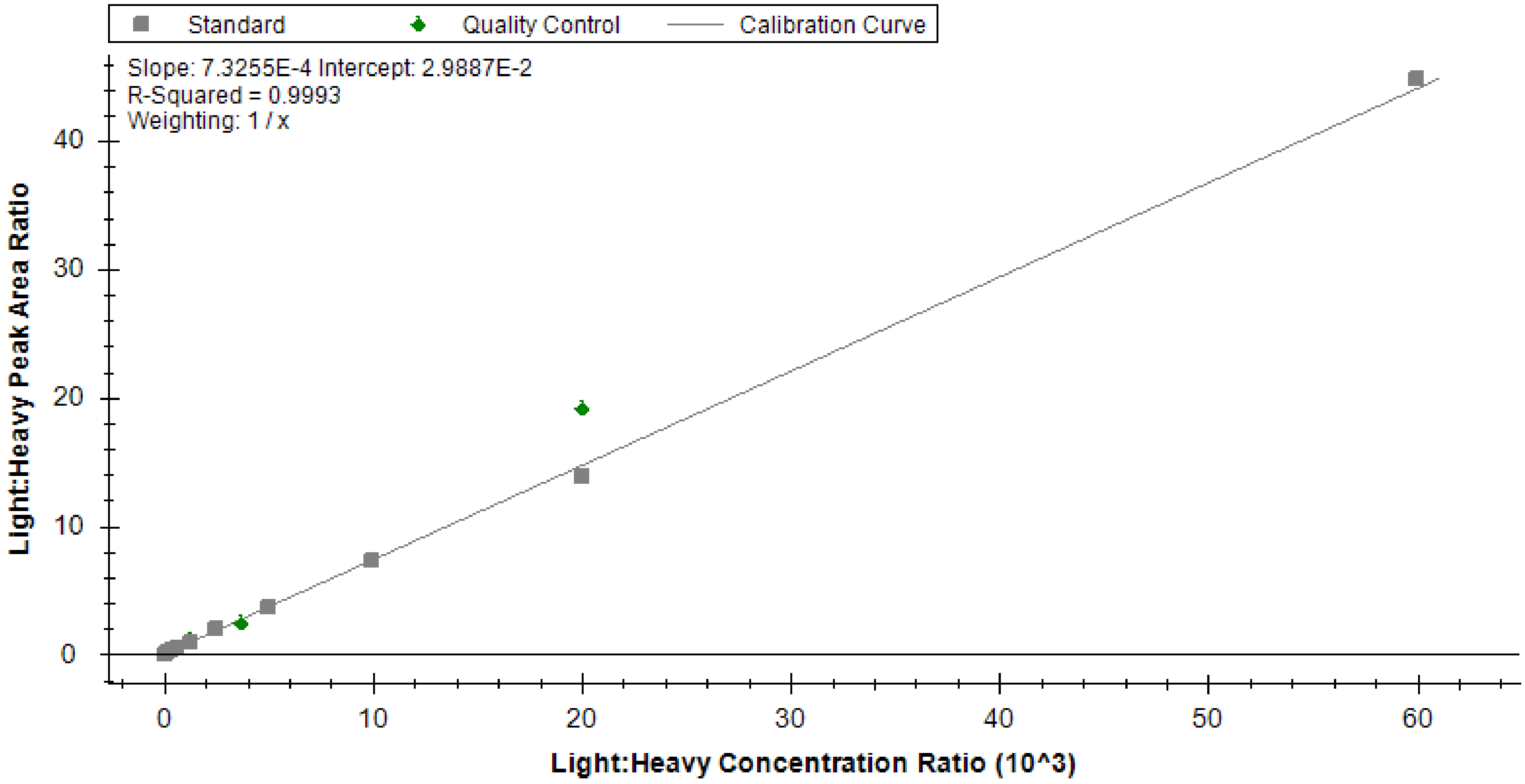

AME1: NDEDLTTR

Quantifier ion: y5+, 482.2225++ → 605.3253+

AMR: 78.125 – 60000 pM

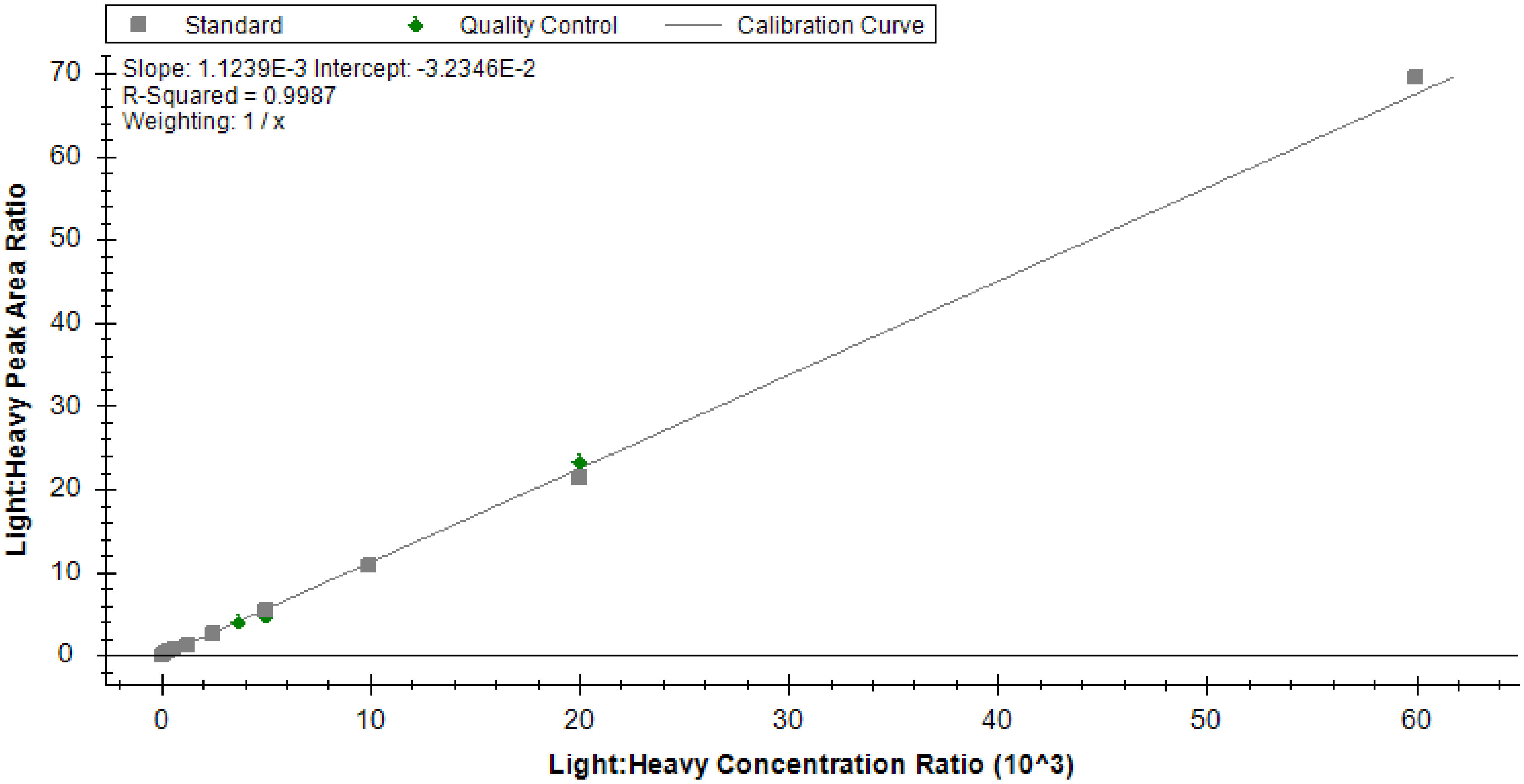

Supplement: Supplementary file 31 [file LSA-2024-03007_SdataFS11.pdf]
